# RNA splicing analysis deciphers developmental hierarchies and reveals therapeutic targets in adult glioma

**DOI:** 10.1172/JCI173789

**Published:** 2024-04-25

**Authors:** Xiao Song, Deanna Tiek, Shunichiro Miki, Tianzhi Huang, Minghui Lu, Anshika Goenka, Rebeca Iglesia, Xiaozhou Yu, Runxin Wu, Maya Walker, Chang Zeng, Hardik Shah, Shao Huan Samuel Weng, Allen Huff, Wei Zhang, Tomoyuki Koga, Christopher Hubert, Craig M. Horbinski, Frank B. Furnari, Bo Hu, Shi-Yuan Cheng

**Affiliations:** 1 The Ken & Ruth Davee Department of Neurology, The Lou and Jean Malnati Brain Tumor Institute, The Robert H. Lurie Comprehensive Cancer Center, Simpson Querrey Institute for Epigenetics, Northwestern University Feinberg School of Medicine, Chicago, Illinois, USA.; 2 Department of Medicine, Division of Regenerative Medicine, Sanford Stem Cell Institute, UCSD, La Jolla, California, USA.; 3 Department of Preventive Medicine, The Robert H. Lurie Comprehensive Cancer Center, Simpson Querrey Institute for Epigenetics, Northwestern University Feinberg School of Medicine, Chicago, Illinois, USA.; 4 Metabolomics Platform, Comprehensive Cancer Center, and; 5 Proteomics Platform, Office of Shared Research Facilities, Biological Sciences Division, The University of Chicago, Chicago, Illinois, USA.; 6 Department of Neurosurgery, The University of Minnesota, Minneapolis, Minnesota, USA.; 7 Department of Biochemistry, School of Medicine, Case Western Reserved University, Cleveland, Ohio, USA.; 8 Departments of Pathology and Neurological Surgery, The Lou and Jean Malnati Brain Tumor Institute, The Robert H. Lurie Comprehensive Cancer Center, Northwestern University Feinberg School of Medicine, Chicago, Illinois, USA.

**Keywords:** Cell biology, Oncology, Brain cancer, Molecular biology, RNA processing

## Abstract

Widespread alterations in RNA alternative splicing (AS) have been identified in adult gliomas. However, their regulatory mechanism, biological significance, and therapeutic potential remain largely elusive. Here, using a computational approach with both bulk and single-cell RNA-Seq, we uncover a prognostic AS signature linked with neural developmental hierarchies. Using advanced iPSC glioma models driven by glioma driver mutations, we show that this AS signature could be enhanced by EGFRvIII and inhibited by in situ *IDH1* mutation. Functional validations of 2 isoform switching events in *CERS5* and *MPZL1* show regulations of sphingolipid metabolism and SHP2 signaling, respectively. Analysis of upstream RNA binding proteins reveals PTBP1 as a key regulator of the AS signature where targeting of PTBP1 suppresses tumor growth and promotes the expression of a neuron marker TUJ1 in glioma stem-like cells. Overall, our data highlights the role of AS in affecting glioma malignancy and heterogeneity and its potential as a therapeutic vulnerability for treating adult gliomas.

## Abstract 2

Mutation-driven RNA alternative splicing affects glioma heterogeneity and malignancy and upstream RNA binding proteins provide targets for suppressing tumor growth.

## Graphical abstract

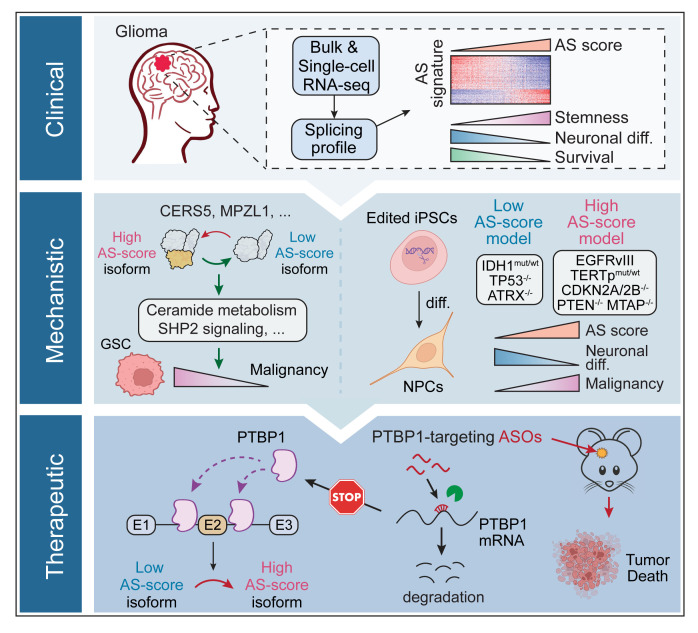

## Introduction

Tumor heterogeneity is a hallmark of glioma and represents one of the major challenges underlying therapeutic failure (1). The genetic heterogeneity of adult gliomas have been incorporated into a refined classification system, as per the 2021 WHO classification of tumors of the central nervous system, which delineates gliomas into 3 subtypes: isocitrate dehydrogenase-WT (IDH-WT) glioblastoma (GBM), astrocytoma with IDH mutation (mut), and oligodendroglioma with IDH mut and a 1p/19q codeletion (2). In addition to the diverse genetic and epigenetic alterations that drive heterogeneous oncogenic programs in gliomas (3), glioma cells also recapitulate multiple neurodevelopmental and lineage differentiation programs, namely “cellular hierarchies”, driving another layer of heterogeneity (4). It has been proposed that IDH-mut gliomas comprise 3 main subpopulations: stem/progenitor-like cells, oligodendrocyte-like (OC-like) and astrocyte-like (AC-like) cells (5, 6). In IDH-WT GBM, the intratumoral heterogeneity is represented by 4 interconvertible cellular states including neural-progenitor-like (NPC-like), oligodendrocyte-progenitor-like (OPC-like), AC-like, and mesenchymal-like (MES-like) states (7). Although there has been significant progress in understanding inter- and intratumoral heterogeneity in gliomas, there are still challenges in leveraging this knowledge to develop effective therapies.

RNA alternative splicing (AS) is a critical mechanism that generates multiple transcripts from a single gene, thereby expanding diversities of the transcriptome (8). With a cell-, tissue-, or developmental-specific regulation, AS is particularly common, conserved in the mammalian nervous system, and contributes to the functional complexity during brain development (9). Several AS-based signatures have been identified in gliomas or GBM that showed association with patient prognosis, tumor recurrence, or immune microenvironment remodeling (10–12). A multi-omics study of GBM by the Clinical Proteomic Tumor Analysis Consortium (CPTAC) reported that the RNA transcript, protein, and phosphorylated protein abundances of genes related to mRNA splicing were upregulated in the classical subtype of GBM, indicating increased RNA splicing activities in a subset of GBM (13). However, our understanding of the relationship between dysregulated AS, tumor heterogeneity, and cellular hierarchies in gliomas remains limited. The biological functions of most protein isoforms generated from glioma-associated AS alterations remain unknown, and there are significant gaps regarding how to target dysregulated AS to treat gliomas.

Here, we use bulk and single-cell RNA-Seq data to determine the influence of dysregulated AS on tumor heterogeneity of adult gliomas and identify a prognostic AS signature associated with the neural developmental hierarchies in GBM and IDH-mut gliomas. We further show that this AS signature can be regulated by mutant EGFR or IDH1 and elucidate the functional mechanisms of AS events in genes *CERS5* and *MPZL1* in promoting glioma malignancy. Lastly, we investigate the involved upstream RNA-binding proteins (RBPs) and identify PTBP1 as a promising therapeutic target to dampen the malignant AS signature while promoting neuronal-like differentiation in glioma cells.

## Results

### Unsupervised splicing analysis in bulk gliomas reveals a prognostic AS signature linked to neural lineage differentiation.

**Figure 1:**
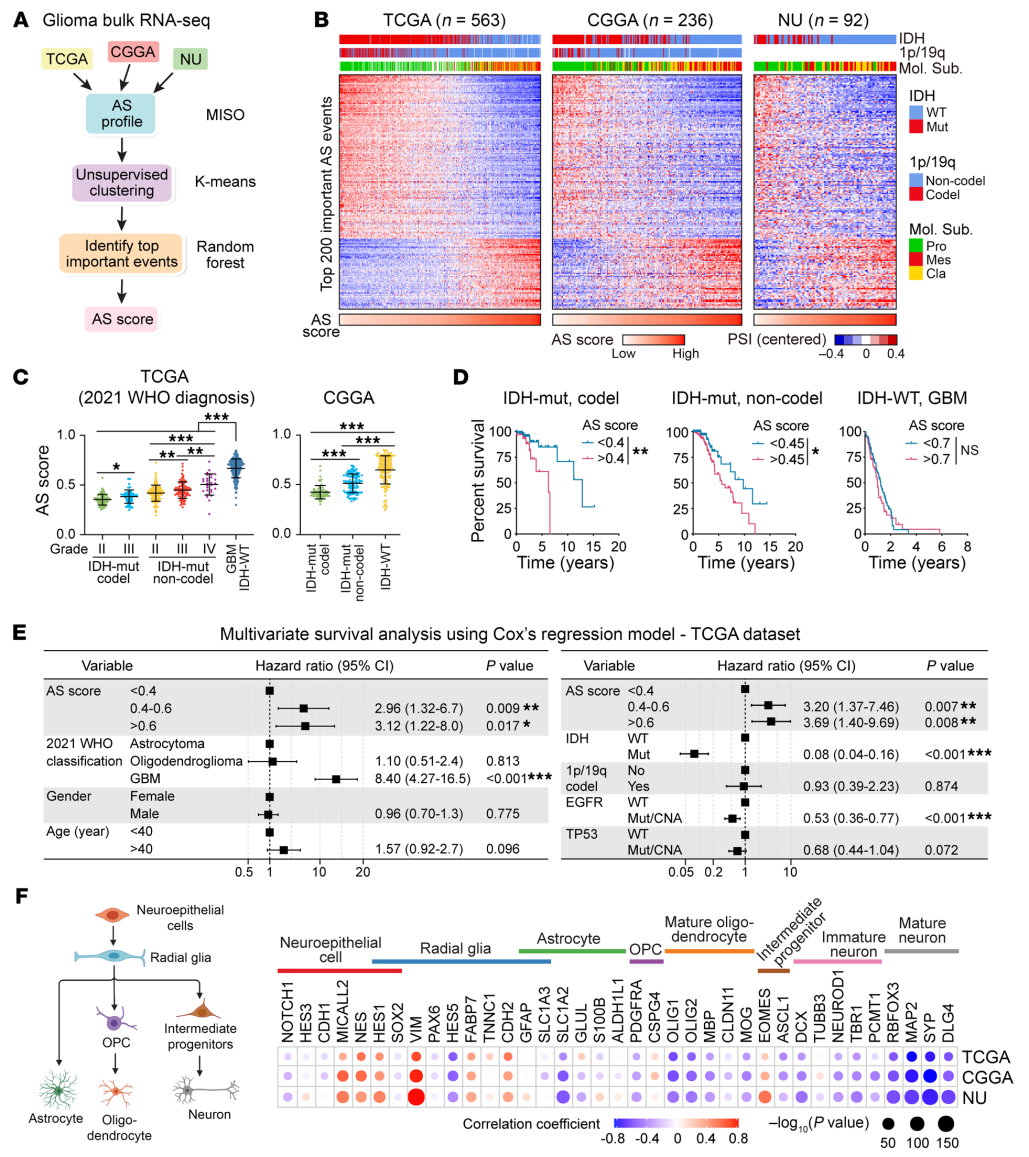
Unsupervised splicing analysis in bulk gliomas reveals a prognostic AS signature linked to neural lineage differentiation. (**A**) Computational pipeline of AS analysis in gliomas. (**B**) Heatmaps showing the PSI values of the 200 AS events across 3 glioma samples. Samples were ordered based on their AS scores. Annotations on top show the association of AS landscape with *IDH* mutation, 1p/19q codeletion and predefined molecular subtyping. Pro, proneural; Mes, mesenchymal; Cla, classical; Mut, mutant; WT, wildtype. (**C**) AS scores of glioma samples in indicated groups from TCGA and CGGA data sets, analyzed using 1-way ANOVA multiple comparisons with correction by controlling the FDR. (**D**) Kaplan-Meier analyses in TCGA gliomas grouped by AS score. Log-rank test was used to compare between groups. (**E**) Multivariate cox regression analysis for overall survival in TCGA glioma samples. HR, hazard ratio; CI, confidence interval. (**F**) The correlation between AS score and the expression of neural lineage markers. Dot sizes indicate the *P* value from spearman correlation analysis, and colors indicate correlation coefficient value. The cartoon on left shows the neural differentiation trajectory. *P < 0.05; **P < 0.01; ***P < 0.001.

To decipher dysregulated AS in glioma heterogeneity, we compiled 3 bulk glioma RNA-Seq data sets, TCGA, CGGA (14), and our previously deposited Northwestern University (NU) glioma data set (15) and quantified Percent Spliced In (PSI) value for each annotated event using MISO software (16) (Figure 1A and Supplemental Tables 1–4; supplemental material available online with this article; https://doi.org/10.1172/JCI173789DS1). We removed samples with poor sequence quality, or that were originally assigned as the “neural” subtype, for potential normal brain contamination (17) and filtered the data to 1,300 AS events with consistent PSI variability across the 3 platforms (Supplemental Figure 1A and 1B). Then, we performed unsupervised k-means consensus clustering in filtered TCGA samples and identified 2 clusters that significantly correlated with patient prognosis (Supplemental Figure 1, C–E). To identify the most important AS events affecting the clustering, we built a random forest model (18) with the 400 most representative samples, identified based on their positive silhouette width, which is a measure of how well a sample is clustered (Supplemental Figure 1F). We selected the top 200 AS events (affecting 170 genes) based on the Mean Decrease Gini (MDG) value, a metric to quantify the importance of each feature in the random forest model, and visualized the splicing pattern of these 200 events across the 3 data sets, which revealed a continuum rather than a bimodal distribution (Figure 1B, Supplemental Figure 1, G and H, and Supplemental Table 5). We further developed an AS score based on the PSI values of the top 40 events (affecting 36 genes) with highest MDG values among these 200 events (Supplemental Figure 1I). In all 3 data sets, the splicing pattern of the 200 events as well as the AS scores were significantly associated with updated 2021 WHO tumor grades, *IDH1* mutation status, and the predefined molecular subtyping (Figure 1, B and C). Moreover, a higher AS score was associated with worse overall survival in patients in both TCGA and CGGA data sets (Figure 1D and Supplemental Figure 1J). Multivariable survival analyses using a Cox regression model showed that our AS score system is an independent prognostic factor for glioma patient survival after controlling for the 2021 WHO classification, gender, age, *IDH* mutation, 1p/19q codeletion, and genetic alterations in *EGFR* and *TP53* (Figure 1E and Supplemental Figure 1K). Intriguingly, the AS score is significantly associated with the expression of specific neural lineage markers, showing a positive correlation with markers of neuroepithelial cells and radial glia, but a negative correlation with markers of differentiated lineages, like neuronal lineages (Figure 1F), suggesting a connection between AS and the developmental hierarchy of gliomas.

As an orthogonal validation of splicing estimation, we performed AS analysis in TCGA samples using rMATS (19). A high concordance in PSI prediction among the 200 AS events was observed between the results from MISO and rMATS algorithms (Supplemental Figure 1, L and M), supporting the rigor of our AS analysis.

**Figure 2:**
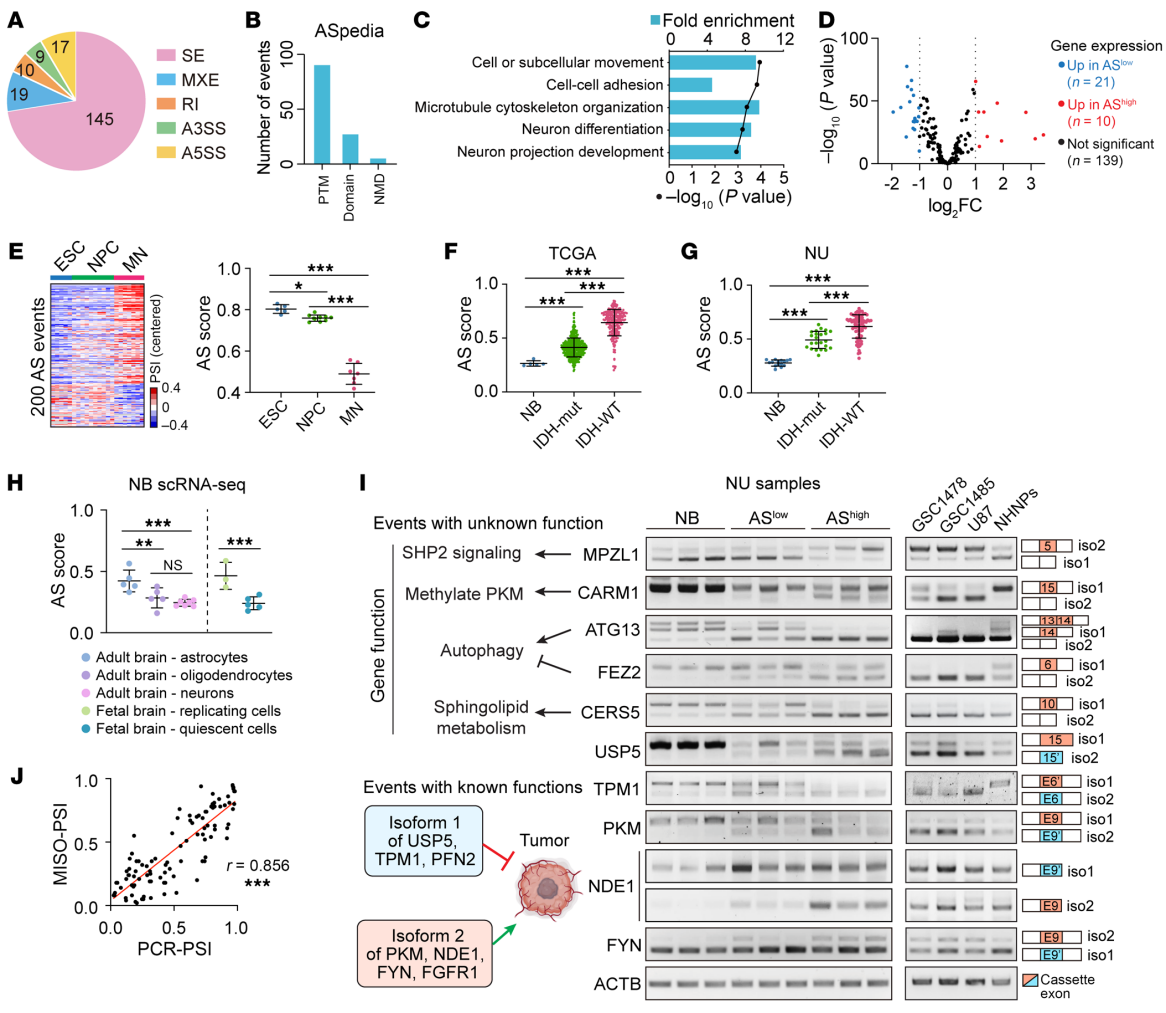
An overview of the 200 events and validation of their AS pattern. (**A**) Distribution of the 200 AS events in each category: SE, skipped exons; MXE, mutually exclusive exons; A5SS/A3SS, alternative 5′/3′ splice sites; RI, retained introns. (**B**) Functional impact of 200 AS events annotated by the ASpedia database. PTM, posttranslational modification; NMD, nonsense-mediated decay. (**C**) Top 5 significantly enriched GO biological processes of the 170 genes. (**D**) Volcano plot for the differential expression of 170 AS-affected genes between samples with high and low AS scores from TCGA data set. (**E**) AS profiling in human ESC-derived neuronal differentiation model. Left, Heatmaps show the AS landscape of 200 events in ESCs, differentiated NPCs, and motor neurons (MNs). Right, AS scores in indicated groups. (**F** and **G**) AS scores in normal brains (NB), IDH-mut, and IDH-WT gliomas from TCGA (**F**) and NU (**G**) data sets. (**H**) AS scores in indicated cell types from scRNA-Seq data of adult and fetal brains. (**I**) RT-PCR analysis with isoform-specific primers for indicated genes in normal brains (NB), NU glioma tissues (AS^lo^ and AS^hi^), GSC/GBM cell lines, and normal human neural progenitors (NHNPs). (**J**) Pearson correlation analysis between MISO-estimated PSI and RT-PCR quantified PSI. Data were analyzed using 1-way ANOVA multiple comparisons with correction by controlling the FDR in **E**–**H**. *P < 0.05; **P < 0.01; ***P < 0.001.

Among the 200 events, skipped exons (SE) and mutually exclusive exons (MXE) were the predominant AS types (Figure 2A). 123 events were annotated in a functional impact database for human AS events (20) that could potentially affect isoform function/expression, including alterations in posttranslational modification, protein domain, or induction of nonsense-mediated decay (Figure 2B). The 200 events affect 170 genes enriched for biological processes related to neuron differentiation and function (Figure 2C), and 139 genes showed no significant change in their total transcript levels between tumors with high or low AS scores (Figure 2D), suggesting that their functions are regulated at the AS level. Moreover, the differences in AS landscapes between gliomas with high or low AS scores is comparable to the AS switch observed in a neuronal differentiation model from human embryonic stem cells (ESCs) (21), further supporting the linkage of this AS signature with neuronal lineage differentiation (Figure 2E and Figure 1B). As expected, the AS score of normal adult brain was found to be markedly lower than those of gliomas (Figure 2, F and G and Supplemental Figure 2A). Although no significant difference in AS score between bulk fetal and adult brain tissues (22) were observed (Supplemental Figure 2B), single-cell (sc) RNA-Seq profiles (23) revealed a lower AS score in neurons and OC compared with ACs from adult brains, while the quiescent neurons exhibited a lower AS score compared with the replicating neuronal progenitors from fetal brains (Figure 2H).

For each event, we designated the isoform associated with a low AS score as isoform 1 (iso1), and the isoform associated with a high AS score as isoform 2 (iso2). Interestingly, in 7 events whose biological impact has been previously reported (24–30), either the iso1 inhibits tumor growth or iso2 promotes tumorigenicity (Figure 2I and Supplemental Figure 2C). To validate our AS analysis, we selected ten events, including 5 events with known isoform-specific functions (USP5, TPM1, PKM, NED1, and FYN) and 5 events (MPZL1, CARM1, ATG13, FEZ2, and CERS5) whose isoform-specific functions in cancer are less studied but occur in genes implicated in critical cancer-related processes (Figure 2I). We observed AS changes in these 10 genes across the normal brain tissue, low-AS score gliomas, and high-AS score GBMs, validating our bioinformatic analyses with significant correlations between MISO-estimated PSI and PCR-quantified PSI (Figure 2J). We also detected these events in GBM stem-like cells (GSC) 1478, GSC1485, the GBM line U87, and normal human neural progenitors (NHNPs; Figure 2I). NHNPs express more iso1 and less iso2 than GSC/GBM lines in most detected events. In addition, those 10 events were alternatively spliced during ESC-neuronal differentiation and all of them were significantly associated with patient outcomes (Supplemental Figure 2D).

### Intra-tumoral AS heterogeneity is associated with developmental hierarchy in gliomas.

**Figure 3:**
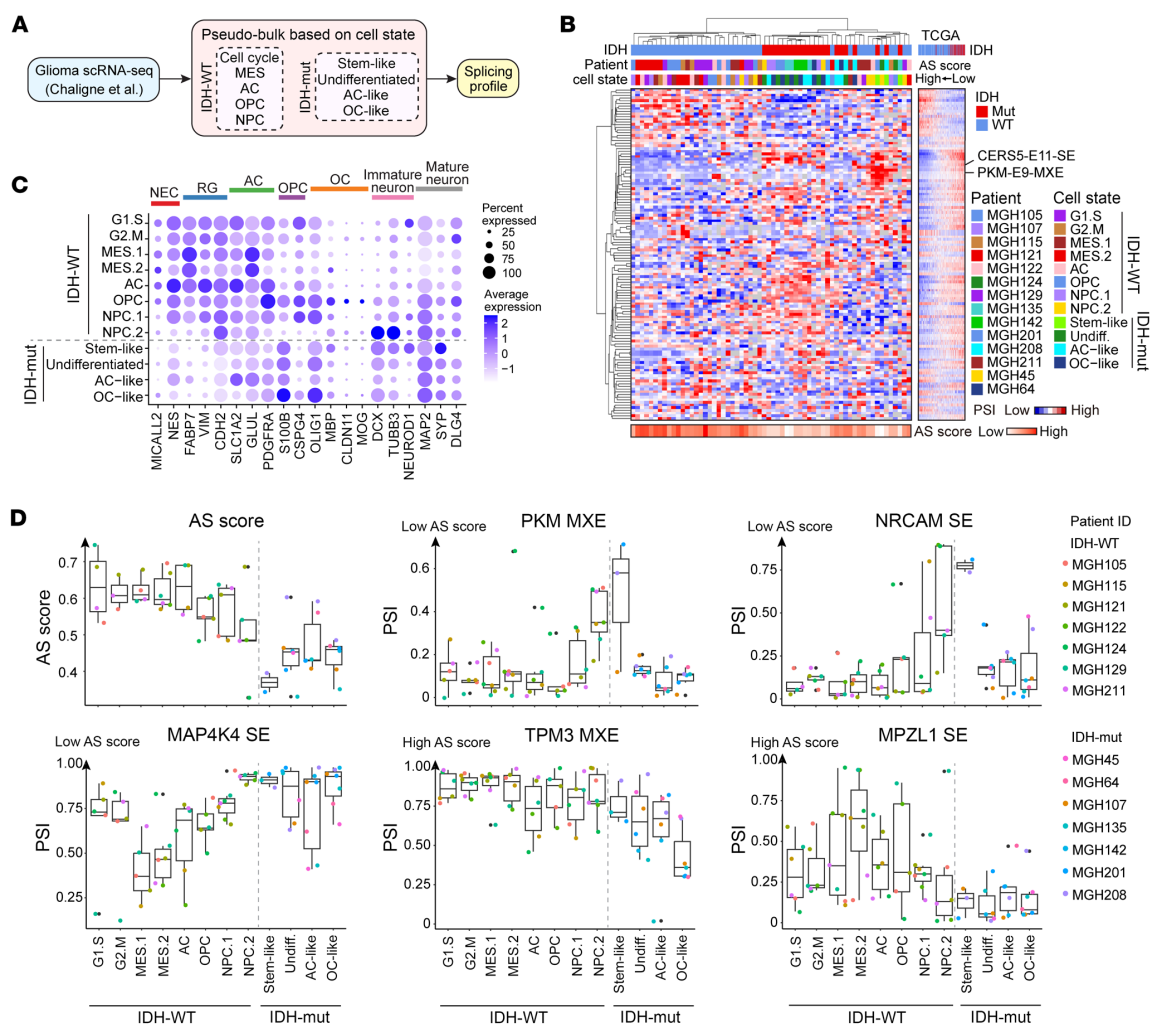
Intratumoral AS heterogeneity is associated with the developmental hierarchy in glioma. (**A**) Computational pipeline of AS analysis using a cell-state based pseudobulk strategy in scRNA-Seq data of gliomas. (**B**) Hierarchical clustering analysis with the PSI data of events in pseudobulks. The heatmap on the right illustrates the PSI data of events at the same order in TCGA samples. (**C**) Expression of neural lineage markers in each cell state. Dot sizes indicate the percentage of cells in each group expressing the gene, and colors indicate average expression levels. NEC, neuroepithelial cells; RG, radial glia; AC, astrocyte; OPC, oligodendrocyte progenitors; OC, oligodendrocytes. (**D**) Box plots showing the AS score and PSI distribution of representative AS events in each cell state. The box representing the interquartile range of the data, the line within the box representing the median, and the whiskers extending to the most extreme data points within 1.5 times the interquartile range. Individual data points beyond this range are shown as dots. The color of the dots represents the patient.

To investigate how the AS signature that we defined from bulk gliomas also contributes to the intratumoral heterogeneity in gliomas, we analyzed published full-length scRNA-Seq data from 7 patients with IDH-WT GBM and 7 patients with IDH-mut glioma (31). To increase the read coverage for AS estimation, we integrated cells from the same cellular state within each patient as a pseudobulk before AS profiling (Figure 3A and Supplemental Figure 3A). The MES state has been subdivided into 2 categories: hypoxia-independent MES.1 and hypoxia-dependent MES.2. Similarly, the neural progenitor cell (NPC) module has been further divided into 2 groups: NPC.1, which expresses OPC-related genes and NPC.2, which expresses genes related to neuronal lineage (31). The number of detected events significantly increased by utilizing this pseudobulk strategy compared with analysis at sc resolution (Supplemental Figure 3B). Then we performed a hierarchical clustering analysis with the PSI data of detected events from our 200-event list in each cell state-based pseudobulk (Figure 3B). Most of the cellular states between IDH-WT and -mut tumors were separated from each other and showed similarity with the AS landscapes observed in TCGA bulk RNA-seq data. Surprisingly, the NPC.2 pseudobulks from IDH-WT tumors clustered together with the stem-like pseudobulks from IDH-mut tumors, displaying similar AS patterns in genes like *CERS5* and *PKM*, as well as a comparably low AS score (Figure 3B). Gene expression analysis of neuronal lineage markers demonstrated that most IDH1-WT cells expressed high levels of stem/progenitor or AC markers, except for NPC.2, which expressed immature neuron markers, similar to the IDH1-mut stem-like subpopulations (Figure 3C), further supporting the link between neuronal differentiation and our AS signature.

The AS score was relatively lower in all cell states from IDH-mut versus IDH-WT tumors (Figure 3D). However, when focusing on specific events, they exhibited a heterogeneous splicing pattern across different cellular states (Figure 3D and Supplemental Figure 3C). For instance, NPC.2 cells of IDH-WT tumors and stem-like cells of IDH-mut tumors exhibited a similar splicing pattern in genes *PKM*, *NRCAM*, and *PICALM*, which differed from the splicing pattern observed in other cellular states. IDH-WT MES cells exhibited differential AS of *MAP4K4*, while IDH-mut OC-like cells exhibited differential AS of *TPM3*. Overall, our AS signature not only contributes to the intratumoral heterogeneity but is also linked to developmental hierarchies within each glioma.

### Glioma driver mutations modulate AS landscape and neural developmental programs.

**Figure 4:**
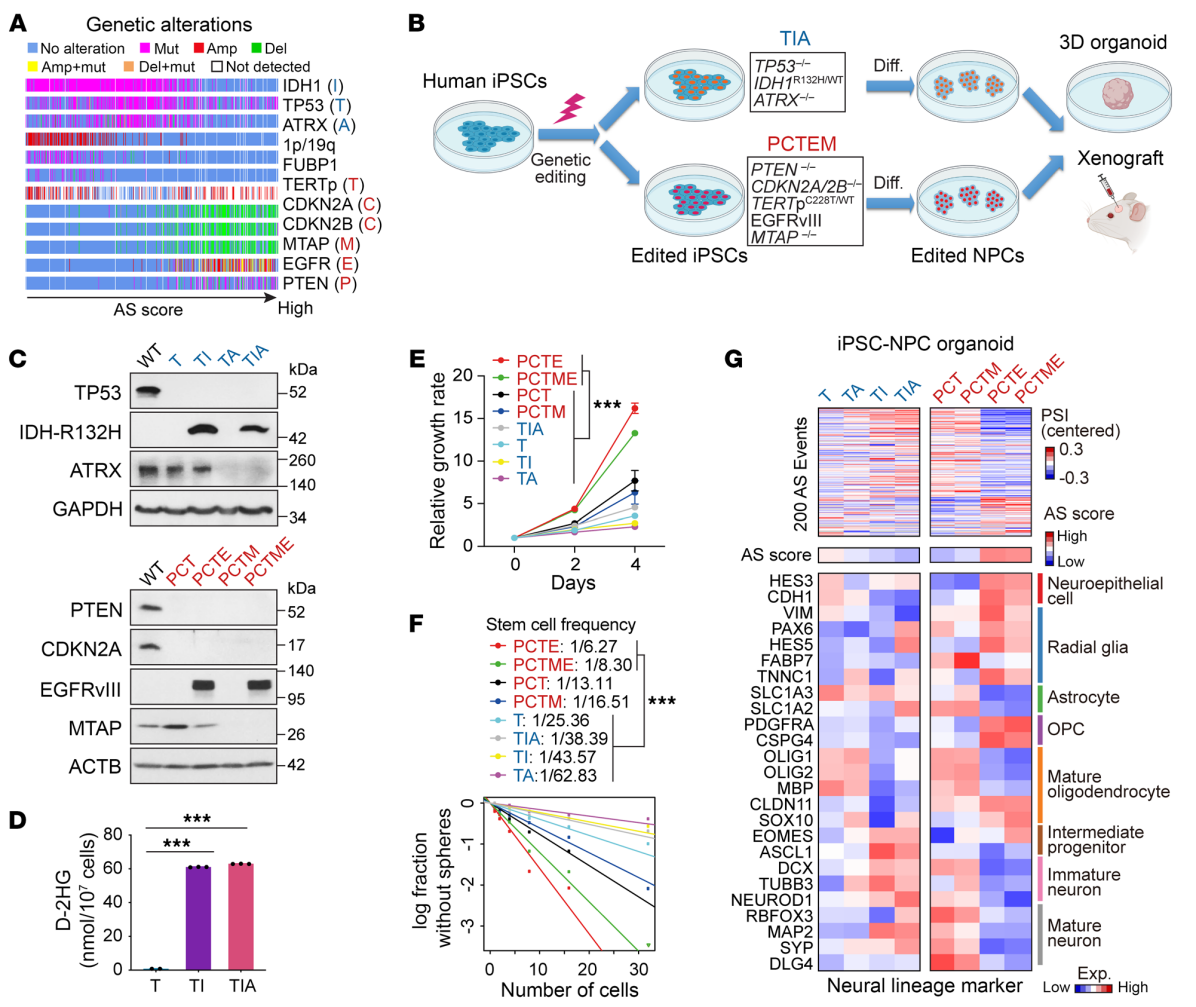
Glioma driver mutations modulate AS landscape and neural developmental programs in iPSC-based glioma models. (**A**) Mutational landscape of frequent somatic alterations in TCGA glioma samples ordered by AS score. (**B**) Workflow of iPSC editing, NPC induction, and in vitro and in vivo model system. (**C**) IB for edited iPSCs. WT, WT; T, *TP53*^–/–^; TI, T+*IDH1*^R132H/WT^; TA, T+*ATRX*^–/–^; TIA, TI+*ATRX*^–/–^; PCT, *PTEN*^–/–^ *CDKN2A/2B*^–/–^, *TERT*p^–/–^; PCTE, PCT+EGFRvIII-OE; PCTM, PCT+*MTAP*^–/–^; PCTME, PCTM+EGFRvIII-OE. (**D**) Detection of intracellular D-2HG in edited NPCs. *n* = 2–3. (**E**–**F**) Cell proliferation (**E**, *n* = 3–6) and self-renewal ability (**F**) of edited NPCs. (**G**) Heatmap showing the AS landscapes and the expression of neural lineage markers in iPSC organoids harboring indicated mutations. Data were analyzed using 2-tailed unpaired *t* test in **D**, 2-way ANOVA in **E**, and likelihood ratio test of single-hit model in **F**. ***P < 0.001.

Considering the association between AS signature and *IDH* mutation status (Figure 1B), we speculated that genetics might play a role in shaping the heterogeneous AS landscape. By analyzing TCGA data, we identified 3 genotypes that exhibited increasing AS scores: (a) *IDH1*-mut + *TERT* promoter–mut (*TERT*p-mut) + 1p/19q co-del + *CIC*/*FUBP1*-mut; (b) *IDH1*-mut + *TP53*-mut + *ATRX*-mut; (c) *TERT*p-mut, *CDKN2A*/*CDKN2B*/*MTAP-del*, *EGFR*-amp/mut + *PTEN*-mut data (Figure 4A and Supplemental Figure 4A). To assess the impact of these genetic alternations on the AS landscape, we utilized a human iPSC-derived glioma “avatar” model (32, 33). Given the difficulty of modeling 1p/19q codeletion, we focused on the latter 2 genotypes. Using CRISPR/Cas9, we developed edited human iPSCs harboring *TP53*^–/–^, *IDH1*^R132H/WT^, *ATRX*^–/–^ (T, I, A), or *PTEN*^–/–^, *CDKN2A/2B*^–/–^, *TERT*p^C228T/WT^, EGFRvIII-overexpression (OE), plus *MTAP*^–/–^ (P, C, T, E, M; Figure 4, B–D and Supplemental Figure 4B). Edited iPSCs were then differentiated into NPCs (33). The differentiation status was confirmed by the downregulation of pluripotency markers and upregulation of NPC markers in all edited NPCs except for iPSC^PCTE^-NPC and iPSC^PCTME^-NPC, which failed to induce *PAX6* expression (Supplemental Figure 4C). Considering that the EGFRvIII-OE in iPSCs might influence *PAX6* expression during NPC induction, we generated 2 other NPCs, iPSC^PCT^-NPC^E^ and iPSC^PCTM^-NPC^E^, in which the EGFRvIII was overexpressed at the NPC stage, and the *PAX6* expression was significantly upregulated (Supplemental Figure 4, C–E). We characterized these 2 edited NPCs as “PCTE” and “PCTME” models.

The edited NPCs representing each of the 8 genotypes (IDH1-mut: T, TI, TA, TIA; IDH1-WT: PCT, PCTM, PCTE, and PCTME) were assessed for their cellular properties. Compared with other edited NPCs, PCTE and PCTME displayed an increased capacity for proliferation and self renewal (Figure 4, E and F), validating the established oncogenic function of EGFRvIII. Next, we evaluated the AS landscape in these edited NPCs by using a 3D organoid model to recapitulate the composition and architecture of primary gliomas (Supplemental Figure 4F) (34). We performed transcriptome analysis on those organoids and focused on our 200-event AS landscape. Intriguingly, we observed that *IDH1* mutation had a modest impact on the AS profile, shifting it toward a lower AS score, whereas EGFRvIII significantly drove an AS signature with an increased score (Figure 4G and Supplemental Figure 4G). Consistently, *IDH1* mutation induced higher expression of neuronal lineage markers, while EGFRvIII blocked the differentiation of all 3 lineages, keeping cells in a stem/progenitor stage (Figure 4G).

**Figure 5:**
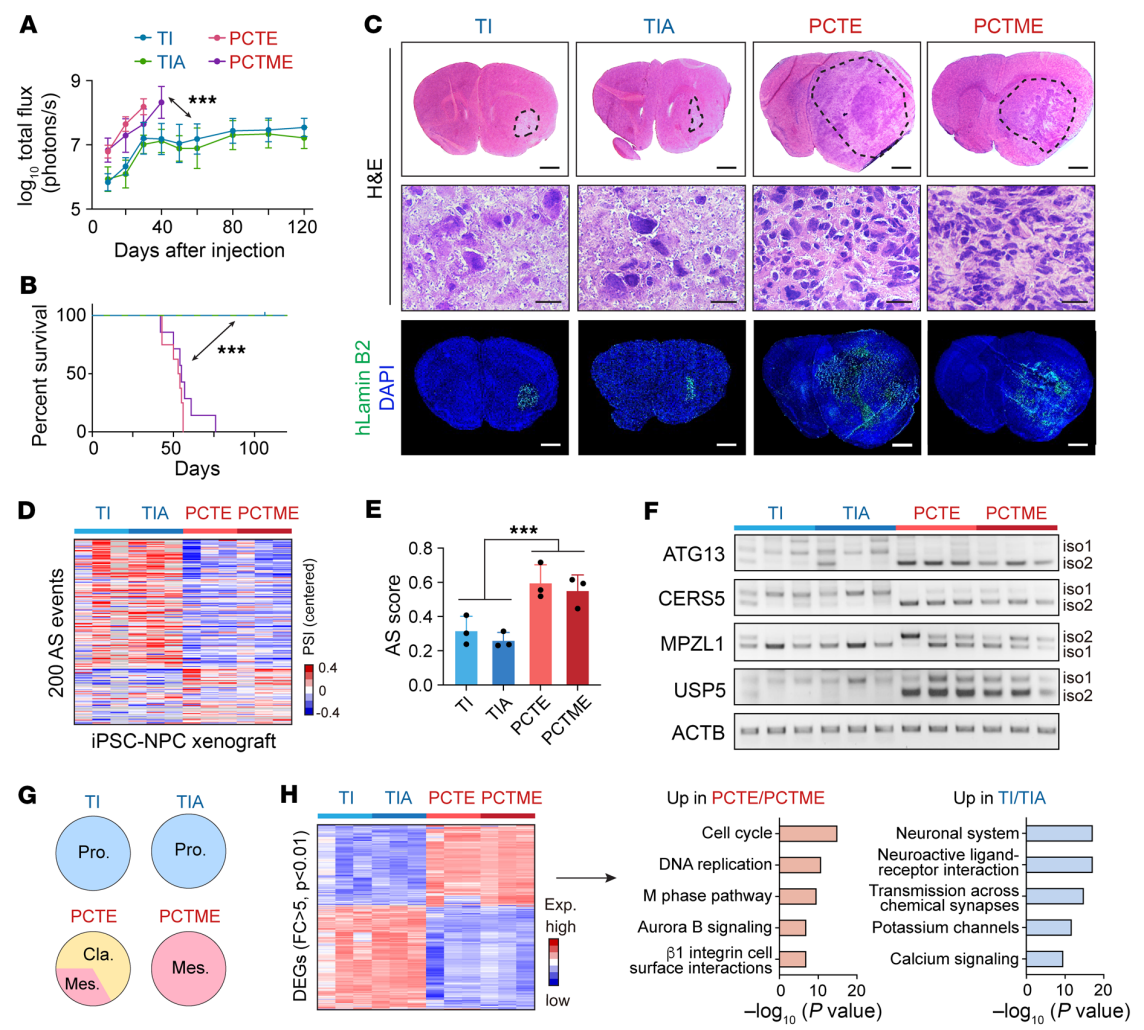
In vivo glioma models from edited iPSCs recapitulate the gene expression and AS signatures of clinical gliomas. (**A**) Quantification of bioluminescent intensity emitted from indicated intracranial xenografts. *n* = 5–6. (**B**) Kaplan-Meier analysis of tumor-bearing mice. Log-rank test was used to compare between groups. *n* = 5–8. (**C**) Representative images of H&E (upper and middle) and IF (lower) staining with a human-specific anti-laminin B2 antibody on brain sections from tumor-bearing mice (*n* = 5–6). Scale bars, upper and lower panel, 1 mm; middle panel, 20 μm. (**D** and **E**) AS landscape of the 200 events (**D**) and AS scores (**E**) in iPSC xenografts harboring indicated mutations from RNA-Seq data. (**F**) RT-PCR analysis with human-specific primers in intracranial xenografts from edited NPCs. (**G**) Subtyping results of iPSC-derived glioma xenografts based on a previously reported molecular subtype signatures using GlioVis subtyping tools. *n* = 3. (**H**) Left, Heatmap showing the differentially expressed genes between TI/TIA and PCTE/PCTME xenografts. Right, Top 5 significantly enriched GO biological processes of the differentially expressed genes between TI/TIA and PCTE/PCTME xenografts. Data were analyzed using 2-way ANOVA in **A**, log-rank test in **B**, and 2-tailed unpaired *t* test in **E**. ***P < 0.001.

Consistent with the in vitro behaviors, NPC PCTE and PCTME xenografts grew faster and shortened mouse survival compared with TI and TIA tumors (Figure 5, A and B and Supplemental Figure 4H). H&E and immunostaining for human-specific LaminB2 confirmed tumor formation in all groups (Figure 5C). Xenograft transcriptome analysis confirmed the differential AS profiles between mutant *IDH1*-driven and EGFRvIII-driven tumors, which recapitulated the clinical AS landscape (Figure 5, D–F). Additionally, all TI/TIA xenografts were assigned to the “proneural” subtype, while PCTE/PCTME xenografts were either in the “classical” or “mesenchymal” subtype (Figure 5G). Genes with elevated expression in PCTE/PCTME tumors compared with TI/TIA tumors were enriched for biological processes related to cell division, while genes upregulated in TI/TIA tumors were involved in neuronal function (Figure 5H). This analysis indicated that the iPSC-based model of gliomas displayed distinct mutation-dependent variation in their transcriptome, which recapitulated the gene expression and AS signatures of clinical gliomas.

To investigate whether mutant EGFR could drive AS changes in the *IDH1*-mut genetic background, we overexpressed EGFRvIII in the TIA model. Our findings revealed that EGFRvIII substantially enhanced cell proliferation and induced AS changes in the *IDH1*-mut background, similar to its effects observed in the PCT background (Supplemental Figure 4, I–K). Nevertheless, the introduction of the *IDH1*-R132H mutation into *IDH1*-WT GSC1478 cells did not induce AS changes in detected genes and exhibited no impact on cell proliferation or in vivo tumorigenesis (Supplemental Figure 4, L–Q). These data highlighted the regulatory role of EGFRvIII in global AS across different glioma models; in contrast, the impact of *IDH1* mutation on the AS landscape appears to be dependent on the specific genetic background.

### AS of CERS5 and MPZL1 influences the oncogenic potential of glioma cells.

**Figure 6:**
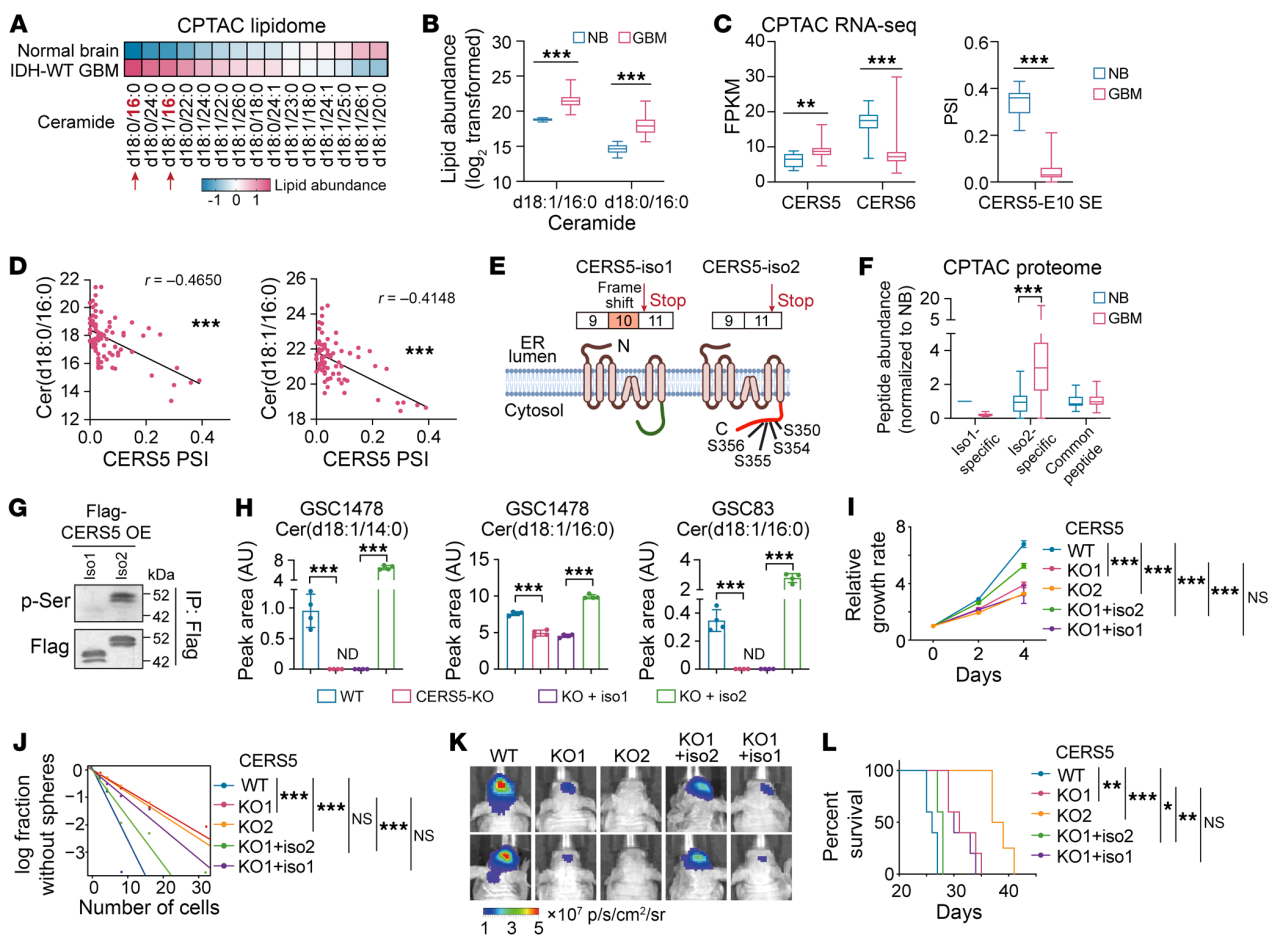
AS of CERS5-E10 affects the ceramide component and oncogenic potential of glioma cells. (**A** and **B**) Ceramide abundance between normal brain and GBM. “d18” represents a sphingoid base with 18 carbons. The number after “:” indicates the presence of double bonds, and the number after “/” denotes the carbon length in the fatty acid chain. (**C**) Gene expression of *CERS5*, *CERS6* (left) and PSI of *CERS5*-E10-SE between normal brain and GBM. (**D**) Spearman correlation analysis between C16-ceramide and PSI of *CERS5*-E10-SE. (**E**) A cartoon showing CERS5 isoforms. (**F**) Abundance of CERS5 peptides analyzed from CPTAC-proteome data. (**G**) IP-IB in GSC46 overexpressed with CERS5 isoforms. (**H**) Lipid-MS analysis of ceramides abundance in GSCs. *n* = 4. ND, not detected. (**I**–**L**) Effects of CERS5-KO and rescue on proliferation (**I**, *n* = 3–6), sphere-formation (**J**), xenograft growth of GSC1478 (**K**, representative BLI at 18 days after inoculation) and mouse survival (**L**, *n* = 4–5). Data were analyzed using 2-tailed unpaired *t* test in **B**, **C**, **F**, and **H**, 2-way ANOVA in **I**, likelihood ratio test in **J**, and log-rank test in **L**. In **B**, **C**, and **F**, the box represents the interquartile range, the line within the box represents the median, and the whiskers extending to the maximum and minimum values. *P < 0.05; **P < 0.01; ***P < 0.001.

To investigate the biological relevance of the AS signature we identified, we first assessed the exon skipping event in *CERS5* exon 10 (E10). Ceramide, the building block of all sphingolipids and a bioactive intermediate in signal transduction, is synthesized by a family of 6 ceramide synthases, CERS1–6, where each generate ceramides with specific N-acyl chain lengths (35). Previous reports indicate chain length–dependent function of ceramides in tumor growth and apoptosis (36). From CPTAC lipidome data (13), we found a significant alteration in the abundance of ceramides with distinct chain lengths (Figure 6A and Supplemental Figure 5A). Specifically, C16-ceramide was identified as the most highly upregulated species in GBM compared with normal brain tissue (Figure 6B). C16-ceramide is synthesized by CERS5 and CERS6, but the overall expression change of these 2 genes could not fully explain the upregulation of C16-ceramide in GBM (Figure 6C and Supplemental Figure 5B). Instead, E10 of *CERS5* is alternatively spliced between GBM and normal brain tissue and the PSI of *CERS5*-E10 is significantly correlated with C16-ceramide abundance (Figure 6D and Supplemental Figure 5C), indicating an isoform-specific function of CERS5 in increasing the levels of C16-ceramide in GBM.

CERS5 is an ER membrane protein consisting of 5 predicted transmembrane segments. The AS of *CERS5*-E10, a nontriplet exon whose inclusion causes a frameshift and an alternative stop code in the last exon (E11), generates 2 isoforms with distinct cytosolic C termini (Figure 6E). With the CPTAC proteomic data, we confirmed that normal brain preferentially expresses the isoform including E10 (iso1), while GBM preferentially expresses the isoform lacking E10 (iso2; Figure 6F), which contains 4 serine phosphorylation sites encoded by E11 (Figure 6E and Supplemental Figure 5D). Phosphorylation at these 4 serine sites is required for the increased C16-ceramide level after CERS5 OE (37). As expected, we detected serine phosphorylation of exogenously expressed CERS5 iso2, but not in iso1, in GSCs (Figure 6G). To study the isoform-specific function, we knocked out CERS5 in GSCs, in which iso2 is the dominant isoform (Figure 2I), then overexpressed either iso1 or iso2 in the KO cells (Supplemental Figure 5, E–G). Ablation of CERS5 resulted in a significant reduction of C14 and C16-ceramides, which could be rescued by reexpression of iso2 but not iso1 (Figure 6H and Supplemental Figure 5H). Further, CERS5 KO inhibited GSC cell proliferation and sphere-forming frequency and suppressed brain xenograft growth, extending animal survival. Reexpression of CERS5 iso2, but not iso1, rescued the inhibition by CERS5 KO on GSC tumorigenicity (Figure 6, I–L and Supplemental Figure 5, I and J).

**Figure 7:**
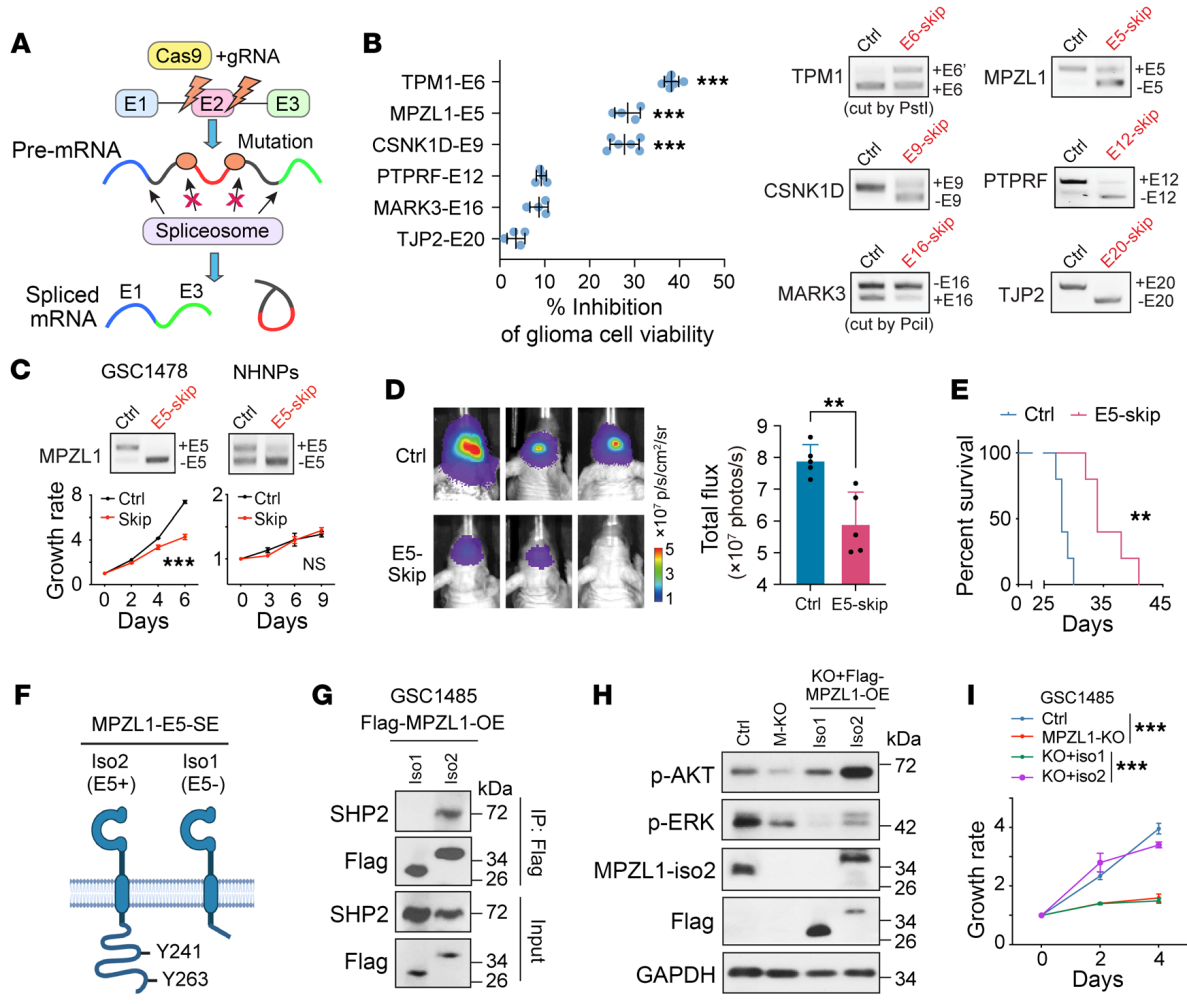
Modulation of MPZL1 splicing affects its interaction with SHP2 and the subsequent oncogenic signaling in glioma cells. (**A**) Scheme showing CRISPR-based splicing modulation. (**B**) Left, effects on cell viability of GSC1485 by skipping of indicated exons. Right, RT-PCR. *n* = 4–6. (**C**) Effect of *MPZL1*-E5 skipping on the proliferation of indicated cells. Upper, RT-PCR. Lower, growth curve. *n* = 4. (**D** and **E**) Effects of *MPZL1*-E5 skipping on xenograft growth of GSC1478 (**D**, representative BLI and quantification, *n* = 5) and mouse survival (**E**, *n* = 5). (**F**), A cartoon showing MPZL1 isoforms. (**G**) IP-IB in GSC1485 overexpressed with MPZL1 isoforms. (**H** and **I**) Effects of MPZL1-KO and rescue on signaling pathways (**H**) and proliferation (**I**, *n* = 3–4) of GSC1485. Data were analyzed using 2-tailed unpaired *t* test in **B** and **D**, 2-way ANOVA in **C** and **I**, and log-rank test in **E**. **P < 0.01; ***P < 0.001.

To further investigate AS signature event, we developed a CRISPR-based AS manipulation method to screen functional events (Figure 7A) (27). We selected 8 AS candidates based on their importance scores calculated from the random forest model as well as CRISPR targeting feasibility of their splice sites (Supplemental Figure 5D) and successfully induced the exon skipping in 6 candidates, confirmed by Sanger sequencing (Figure 7B and Supplemental Figure 5K). We showed that the induced exon skipping of *TPM1*-E6, *MPZL1*-E5, or *CSNK1D*-E9 significantly inhibited GSC1485 cell viability. Of note, the induced skipping of *MPZL1*-E5 inhibited in vitro cell proliferation as well as in vivo tumorigenesis in GSC1478 (Figure 7, C–E). However, normal NHAs and NHNPs do not require *MPZL1*-E5 inclusion for their viability (Figure 7C and Supplemental Figure 5L). MPZL1 was identified as a binding protein of tyrosine phosphatase SHP2 and has been demonstrated to be upregulated in various cancers and promote cell proliferation and migration (38–40). Compared with the E5-excluded iso1, the E5-included iso2 contains an extended C-terminus that harbors 2 tyrosine (Y) residues, Y241 and Y263 (Figure 7F). Phosphorylation at Y241 and Y263 of MPZL1 was shown to mediate its interaction with SHP2 (41). We confirmed that only iso2 but not iso1 could bind to SHP2 in GSCs (Figure 7G and Supplemental Figure 5M). SHP2 regulates key oncogenic pathways, including RAS-MAPK and PI3K-AKT, downstream of several receptor tyrosine kinases (42). Indeed, p-AKT and p-ERK were decreased in MPZL1-KO GSC1485 cells compared with control cells, whereas reexpression of exogenous MPZL1 iso2, but not iso1, in MPZL1-KO cells enhanced both p-AKT and p-ERK (Figure 7H). Further, the exogenous expression of iso2, but not iso1, rescued the MPZL1 KO–impaired cell growth in GSCs (Figure 7I).

### A group of RBPs modulate the AS landscape in glioma.

**Figure 8:**
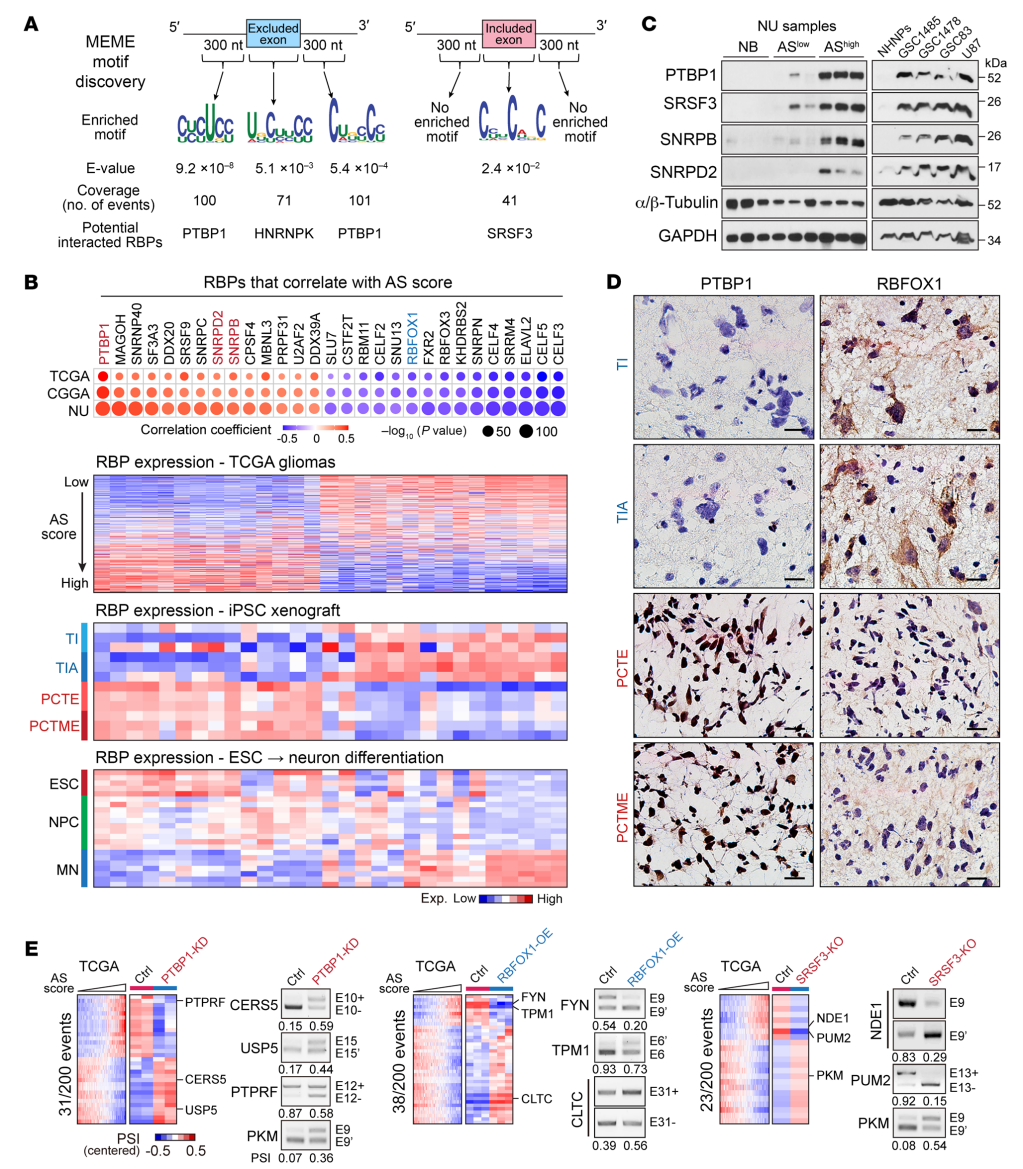
A group of RBPs modulate the AS landscape in glioma. (**A**) Motif analysis around the splice sites of the exons from the 200 events and predicted binding RBPs. (**B**) Upper, dot plot showing the correlation between AS score and the expression of each RBPs. Dot sizes indicate the *P* value from spearman correlation analysis, and colors indicate correlation coefficient value. Bottom, heatmap showing the expression of each RBPs in TCGA gliomas, iPSC glioma xenografts, and human ESC to MN differentiation model. (**C**) IB analysis of indicated RBPs in normal brains (NB), NU glioma tissues with low or high AS scores (AS^lo^ and AS^hi^), and indicated cell lines. (**D**) IHC staining shows the expression of PTBP1 and RBFOX1 in iPSC glioma xenografts. Scale bars: 20 μm. (**E**) Heatmaps show the PSI distribution of the events that are from the 200 events and affected by PTBP1-KD, RBFOX1-OE, or SRSF3-KO. RT-PCR shows the validation of AS changes in GSC1485 with indicated treatment. The numbers below the PCR plots show the PCR-quantified PSI values.

To identify the upstream regulator(s) of the identified AS signature, we analyzed the sequence characteristics surrounding the splice sites of the 200 events. We focused on the SE and MXE events and segregated the exons into those that are more included in GBM and those that are more excluded in GBM compared with normal brain. There was no significant difference in the splice site strength between included and excluded exons calculated with 3 different scoring models (Supplemental Figure 6A). However, included exons had substantially lower GC content upstream of the 3′ splice site (3′SS) compared with the excluded exons (Supplemental Figure 6B). Next, a de novo motif analysis identified potential PTBP1-binding motifs enriched in both the upstream (CYCUCY) and downstream (CUBCCY) intronic regions of the excluded exons, while a potential serine and arginine-rich (SR) splicing factor (SRSF)3–binding motif (CYUCWKC) was found enriched in the exonic regions of the included exons (Figure 8A).

Next, we analyzed the gene expression of 276 splicing-regulating RBPs and identified 29 RBPs whose expression correlated with the AS score in all 3 glioma data sets (Figure 8B). The differential expression of PTBP1, SNRPB, SNRPD2, and SRSF3 was corroborated at the protein level in NU tissue samples and GBM cell lines (Figure 8C). Consistently, these AS score–correlated RBPs are also differentially expressed between mutant IDH1- and EGFRvIII-driven iPSC glioma models (Figure 8, B and D, Supplemental Figure 6, C–E). Of interest, these RBPs exhibit distinct expression patterns during the neuronal differentiation process. Most of the positively correlated RBPs, including PTBP1, gradually decrease their expression during neuronal differentiation, while some of the negatively correlated RBPs, including RBFOX1, show increasing expression (Figure 8B). Splicing analysis in cells affected by PTBP1 KO, RBFOX1 OE, or SRSF3 KO (27, 43, 44) revealed that each of these 3 RBPs regulated a subset of the 200 events (Figure 8E, Supplemental Figure 6F, and Supplemental Table 6). This observation was validated by RT-PCR in GSCs (Figure 8E).

Further, we investigated the expression of these 29 RBPs and their association with AS landscape at the sc level (31). In line with the findings from the bulk RNA-Seq analysis, a hierarchical clustering analysis showed that most IDH-WT cells were distinguished from IDH-mut cells based on their elevated expression of AS positively correlated RBPs, including PTBP1, SNRPB2, and SNRPD (Supplemental Figure 6, G and H). As expected, the NPC.2 subpopulation of IDH-WT tumors clustered together with IDH-mut cells, which is consistent with the finding from the AS-based clustering analysis (Figure 3B). In contrast to the widespread expression of AS positively correlated RBPs, most of the negatively correlated RBPs displayed a scattered expression pattern, with expression limited to a small subset of cells, predominantly IDH-mut. Additionally, we observed significant correlations between certain RBPs and specific AS events at sc resolution (Supplemental Figure 6I).

### Targeting PTBP1 inhibits cell growth and induces neuronal-like differentiation of GSCs.

**Figure 9:**
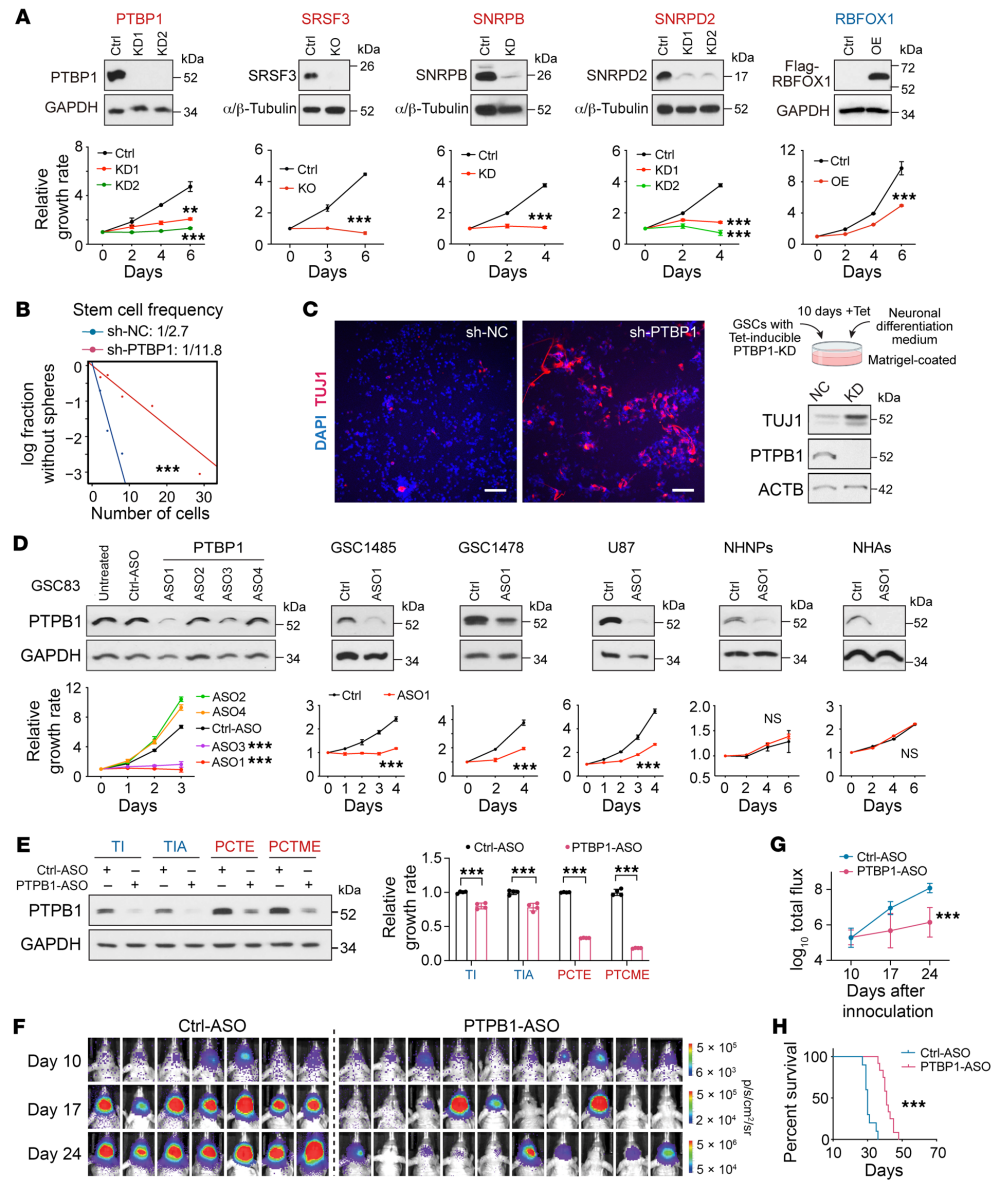
Targeting PTBP1 inhibited cell growth and induced neuronal-like differentiation in GSCs. (**A**) Effect of overexpression (OE), knockdown (KD), or knockout (KO) of indicated RBPs on cell proliferation of GSC1485 (PTBP1, SRSF3, SNRPB, SNRPD2) or GSC1478 (RBFOX1). Upper, IB. Lower, cell proliferation curve. *n* = 2–4. (**B**) Sphere-formation analysis in GSC1478 treated with shRNA-PTBP1 (sh-PTBP1) or a negative control (sh-NC). (**C**) IF and IB analysis of TUJ1 in GSC1478 cells treated with sh-PTBP1 or sh-NC. Scale bars: 100 μm. (**D**) Effects of PTBP1-targeting ASOs on PTBP1 expression (IB, upper) and cell proliferation (lower) in indicated cells. *n* = 3–6. (**E**) Effects of PTBP1-targeting ASO1 on PTBP1 expression (IB, left) and cell proliferation (right) in edited iPSC-derived NPCs with indicated mutations. *n* = 4. (**F**–**H**) In vivo effects of PTBP1-ASO1 on the growth of GSC1478-derived intracranial xenografts (**F**, BLI images of brain glioma xenografts. **G**, quantification) and survival of mice (**H**). *n* = 7–10. Data were analyzed using 2-way ANOVA in **A**, **D**, and **G**, 2-tailed unpaired *t* test in **E**, likelihood ratio test in **B**, and log-rank test in **H**. **P < 0.01; ***P < 0.001.

Next, we determined the biological function of specific RBPs in glioma cells that were positively or negatively correlated with AS scores. KO or KD of PTBP1, SRSF3, SNRPB, or SNRPD2 significantly reduced the proliferation of GSC1485 cells, while OE of RBFOX1, but not CELF4 or SRRM4, inhibited cell growth (Figure 9A and Supplemental Figure 7A). Of interest, a mutual-regulatory network may exist in the expression among these RBPs, wherein SRSF3 KO decreased PTBP1 expression, SNRPB KD reduced SRSF3 and SNRPD2 expression, and SNRPD2 KD led to the dephosphorylation of SRSF3 (Supplemental Figure 7B).

We focused on PTBP1 to further study its function in GSCs due to its strongest correlation with the AS score (Figure 8B). KD of PTBP1 not only induced apoptosis and decreased self renewal in GSC1478 cells, but also induced a neuronal-like morphology and the expression of a neuron marker TUJ1 under a neuronal differentiation culture condition (Figure 9, B and C and Supplemental Figure 7C), which further highlights the association between RBP/AS networks and differentiation programs in glioma. Through the modulation of *CERS5*-E10 AS (Figure 8E), PTBP1 KD led to a significant decrease of C16-ceramides in GSC1478 (Supplemental Figure 7D).

We then assessed the therapeutic vulnerability of targeting PTBP1 in glioma by using an antisense oligonucleotide–based (ASO-based) therapy (45). Compared with a control GFP-ASO, the PTBP1-ASO1 decreased PTBP1 protein level and inhibited in vitro GSC growth but had no effect on NHNPs or NHAs (Figure 9D). Notably, PTBP1 targeting exerted much stronger antitumor effects in *IDH1*-WT iPSC-NPC than in *IDH1*-mut iPSC-NPC (Figure 9E and Supplemental Figure 7E). Consistently, PTBP1 KO had milder effects on the *IDH1*-mut GSCs compared with *IDH1*-WT GSCs (Supplemental Figure 7F), indicating variable dependence on PTBP1-modulated AS program in *IDH1*-WT and -mut tumors.

Next, we assessed the therapeutic effects of the PTBP1-ASO or a control-ASO through intratumoral delivery in an orthotopic xenograft model of GSC1478 cells. Consistently, PTBP1-ASO1 treatment significantly inhibited the growth of orthotopic tumor xenografts and prolonged animal survival (Figure 9, F–H). Further analysis of ASO-treated tumor xenografts showed effective downregulation of PTBP1 expression and induction of apoptosis in PTBP1-ASO group (Supplemental Figure 7G). Taken together, these results suggest that PTBP1-targeting ASO produces a potent antitumor effect in the *IDH1*-WT model.

## Discussion

Considerable progress has been made in uncovering dysregulated AS in adult glioma. Previous studies have been primarily focused on either identifying the AS signature related to glioma subtyping (46), prognosis (10, 47), or recurrence (11) using bulk RNA-Seq data, or conducting functional investigations of specific splicing factors or AS events (12, 48, 49). While some subtyping or prognostic AS signatures have been identified (10, 46, 47), these studies often lack validation across multiple data sets and fail to explore the biological relevance behind the AS signatures. In contrast, our study provides the most comprehensive AS profiling to date in both IDH-WT and -mut adult gliomas through integrating multiple glioma data sets, followed by rigorous validation of the differential splicing patterns in clinical samples, GSCs, and iPSC-based glioma models. scRNA-seq analysis not only validate our data on bulk RNA-Seq but also reveals an intratumoral heterogeneity reflected at the AS level. Moreover, our data reveal a link between the AS landscape and developmental hierarchies in glioma cells. Accumulating evidence revealed that while exhibiting considerable plasticity, gliomas follow a typical neurodevelopmental trajectory where multipotent cells differentiate into neurons, ACs, or OCs (5–7). The identified AS signature is strongly associated with the multipotent state of gliomas and shows a negative correlation with neuronal lineage differentiation. Interestingly, the AS and RBP signatures in NPC.2 subpopulation in IDH-WT tumors share a similarity with IDH-mut tumors, especially the stem-like population. We speculate that the predefined stem-like subgroup in IDH-mut tumors is more neuronal lineage restricted rather than a multipotent population based on their expression levels of lineage markers and lower AS scores. This may explain the reported low rate of dedifferentiation from OC- or AC-like cells to stem-like cells in IDH-mut tumors (31). The distinct AS landscapes observed between IDH-WT and IDH-mut gliomas that link to neurodevelopmental programs suggest variations in the cell-of-origin for these 2 glioma subtypes (50). In brief, with its cell-, tissue-, or developmental-specific regulation, AS provides another perspective for studying the developmental hierarchy in gliomas.

While extensive AS alterations have been identified in cancers, the functional investigation of these changes is often lacking. In this study, we provide mechanistic insights into the isoform-specific functions in *CERS5* and *MPZL1*. CERS5 is known to be responsible for C16-ceramide synthesis that is critical for sphingolipid signaling, tumor growth, and cell apoptosis (35, 36). However, there has been no investigation into the differential AS of CERS5 in cancers and its functional implications for tumorigenesis. Here, we show that the AS of *CERS5* E10 is an important mechanism for the increased C16-ceramide in GBM compared with normal brain. The GBM-associated CERS5 iso2, which contains 4 phosphorylated serine residues at its cytoplasmic tail, is required for the synthesis of C16-ceramide in GSCs. Moreover, CERS5 iso2 but not iso1 promotes GSC growth and self-renewal capacity in vitro and tumorigenicity in vivo. MPZL1 was shown to interact with SHP2 and regulate downstream oncogenic signaling (38, 41). Our data reveal the differential AS of *MZPL1* E5 in gliomas, showing a higher inclusion in high AS score gliomas compared with low AS score gliomas and normal brain tissue. Mechanistically, the E5^+^ isoform includes Y241 and Y263 in its C-terminal tail, which was reported to mediate the interaction between MPZL1 and SHP2 (41). Consistently, we show that the E5^–^ isoform lost its interaction with SHP2 and only the E5^+^ isoform could activate AKT/ERK signaling and promote GSC proliferation. In conclusion, the AS isoforms from the AS signature we defined here affect different hallmarks of cancer including metabolism (PKM2) (25), Src-signaling (FYN) (29), mitosis (NDE1) (27), ceramide synthesis (CERS5), and SHP2-signaling (MPZL1), thereby contributing together to the malignancy and heterogeneity of gliomas.

Our data reveal a link between the oncogenic mutations, neurodevelopmental program, and AS heterogeneity in gliomas. With our hiPSC-derived glioma avatar system, we show that glioma-driven mutations, *IDH1*-mut and EGFRvIII, not only affect the AS landscapes and tumorigenicity, but also modulate the neural differentiation programs. Based on the AS analysis and expression analysis of neural marker genes, we show that EGFRvIII sustains the iPSC-derived NPCs in a multipotent state, while *IDH1*-mut promotes differentiation toward neuronal lineage. Our observation that EGFRvIII impaired the PAX6 induction during iPSC to NPC differentiation also suggests its roles in maintaining stemness. In fact, EGFR signaling is well-known for its crucial role in neural stem cell pool maintenance as well as the inhibition of neuronal differentiation (51, 52). Although *IDH1*-mut was shown to block cell differentiation in tumors of different tissue origins (53, 54), whether it hinders the differentiation of all 3 neural lineages in glioma remains controversial. Here, we find that instead of blocking the differentiation of all neural lineages, *IDH1*-mut shifted differentiation from the glial to the neuronal lineage. Our finding contradicts 2 previous studies that demonstrated the inhibitory effect of *IDH1*-mut on the expression of both astrocytic and neuronal markers (55, 56). The major difference between our model and theirs is that they overexpressed exogenous mutant IDH1, whereas we mutated the endogenous *IDH1* gene locus, thus recapitulating the clinical heterozygous *IDH1* mutation. Moreover, our data corroborates a previous study showing that *IDH1*-mut reduced the expression of GFAP and concomitantly increased the expression of the neuronal lineage marker TUJ1/β3-tubulin (54). Further investigations are needed to determine how mutant IDH1 or EGFR regulate neural lineage differentiation and what functional roles AS plays in this process. A plausible mechanism linking genetic drivers and AS could involve the methylome change induced by *IDH1*-mut, which may affect specific RBPs from binding their target premRNA (57) or the EGFR-activated AKT/SRPK pathway, which increases SR family protein phosphorylation (58).

Last, we identified 2 subsets of RBPs that positively or negatively correlate with the malignant AS signature. Interestingly, these RBPs also exhibit distinct expression patterns during neuron differentiation and correlate with AS landscapes in gliomas at the sc level. We also showed that PTBP1 and RBFOX1 displayed opposing effects on the cell proliferation of *IDH1*-WT GSCs. Context-dependent RBP regulation was also demonstrated when PTBP1 was depleted in *IDH1*-WT or -mut GSCs and iPSC-NPCs. PTBP1 has been described as one of the key RBPs in regulating AS in neural development and known oncogenic AS gene isoforms such as *PKM* and *USP5* in glioma (24, 45, 48). Recent studies show that targeting PTBP1 by ASOs can convert midbrain ACs to dopaminergic neurons and improved Parkinson’s disease in mice (45), and that PTBP1 knockdown promotes neuronal-like differentiation of IDH-WT GBM cell lines (59). Our data not only confirm PTBP1’s role in regulating neuronal-like differentiation in GSCs and but also unveil its previously unknown function in sphingolipid metabolism/C16 ceramide synthesis through regulating *CERS5* E5 SE. Additionally, our study demonstrates that in situ delivery of PTBP1-ASOs successfully inhibited the growth of GSC tumor xenografts and prolonged survival in a mouse model. However, tumor regrew with PTBP1-untargeted cells in the end due to inadequate diffusion of ASOs throughout the entire tumor. Further investigation is necessary to improve the delivery strategy for ASOs to ensure robust tumor targeting. Traditional therapies aim to target tumor-specific features to eradicate specific populations but can induce cellular reprogramming to evade treatment. This is particularly common in tumors with high plasticity, including GBM. Therefore, therapeutics that restrain tumor plasticity, such as differentiating tumor cells to a terminal stage, may be necessary for effective glioma treatment (60). We propose that targeting the AS regulator PTBP1 and/or other key RBPs that regulate AS landscapes represents a potential avenue for neuronal-like differentiation therapy in glioma, particularly in GBMs.

## Methods

### Sex as a biological variable.

Our study examined orthotopic glioma tumor xenografts in male and female animals, and similar findings are reported for both sexes.

### AS analysis in bulk RNA-seq data sets of human gliomas.

RNA-Seq data from TCGA-LGG and -GBM data sets (530 low-grade gliomas, 169 GBMs, and 5 normal brain tissue samples) were downloaded from the National Cancer Institute Genomic Data Commons Legacy Archive (https://portal.gdc.cancer.gov/legacy-archive/). Reads of TCGA-GBM data sets were trimmed to 48 bp from 3′ ends, the same length as TCGA-LGG data sets, to avoid the bias on AS analysis caused by read length variation. RNA-Seq data from the Chinese Glioma Genome Atlas (CGGA) database (14), including 182 LGGs and 143 GBMs, were downloaded from http://cgga.org.cn RNA-Seq analysis was performed in clinical glioma specimens from the Northwestern Nervous System Tumor Bank (NSTB, including 18 LGGs and 85 GBMs) and 15 normal brain tissue specimens from the NIH NeuroBiobank as previously reported (15), referred as NU data set. RNA-Seq data from the Clinical Proteomic Tumor Analysis Consortium (CPTAC) database (13), including 100 GBMs and 9 normal brain tissue samples, were downloaded from PDC portal (https://pdc.cancer.gov/pdc). All the reads from TCGA, CGGA, NU, and CPTAC data sets were aligned to the human genome reference hg19 (genecode_v19) using HISAT2 v2.0.4 (61). The alignments were processed through the MISO v0.5.4 (16) or rMATS v4.0.2 (19) to estimate the PSI (ranging from 0 to 1) value for each AS event. Those samples that showed undetectable PSI values in more than 40% of total annotated events (TCGA: 26/699; CGGA: 12/325; NU: 3/118) were filtered out to ensure high sequence quality for AS analysis. Before performing clustering analysis based on the PSI data of TCGA samples, several filtering criteria were applied to select informative and reliable AS events based on the PSI values calculated by MISO (v0.5.4), including: (a) Max(PSI)–Min(PSI) > 0.6; (b) SD(PSI) >0.1; and (c) the range of 95% confidence intervals (CIs) of the PSI estimates < 0.5 in more than 80% samples. A total of 1,300 AS events satisfied these 3 criteria in TCGA, CGGA, and NU data sets. Samples with a “neuronal” molecular subtype were removed in consideration of potential normal brain contamination. We performed a consensus clustering analysis on GenePattern platform (www.genepattern.org) using the following parameters: clustering algorithm: KMeans; distance measure: Pearson; resampling iterations: 1,000; normalize type: AS row wise, and identified 2 clusters. Next, we built a random forest model (R-package: randomForest, v4.6.14) with the top 400 representative samples selected based on their silhouette width values (R-package: cluster v2.1.0). The 200 most important AS events were selected representing the AS signature according to the MDG value generated by the random forest algorithm. The heatmap visualization of the 200-event signature was conducted using the TreeView tool (v1.2.0). From these 200 events, we further narrowed down to 40 events based on their PSI distribution and MDG values to develop an AS score. The events were categorized into 2 groups according to their PSI distribution among glioma samples: group 1 events show higher PSI values in samples with worse survival; group 2 events show lower PSI values in samples with worse survival (Supplemental Figure 1I). We selected the top 20 events with the highest MDG values in each group to compose the final set of 40 events. We chose to use 40 events instead of including all 200 events to calculate the AS score, taking into consideration their MDG distribution as illustrated in Supplemental Figure 1G, which suggests that the top 40 events are the major contributors of the AS-based clustering in glioma. To calculate the AS score, we subtracted the average PSI value of group 2 events from the average PSI value of group 1 events. The functional prediction of AS events were performed on ASpedia website (http://combio.snu.ac.kr/aspedia/) (20). The neural lineage markers list is obtained from the neural marker booklet available on the Abcam website: https://www.abcam.com/neuroscience/neural-markers-guide TCGA Samples have been reclassified according to the 2021 WHO CNS5 guidelines (62). Genetic information, including TERT promoter mutation, EGFR amplification, and chromosome +7/−10, is required to establish the molecular diagnosis of GBM. However, CGGA and NU samples lack sufficient information in this regard. Consequently, classification of samples from these 2 data sets was conducted based on the presence of IDH-mutation and/or 1p/19q codeletion.

### AS and gene expression analysis in scRNA-Seq data sets.

ScRNA-Seq data of 7 IDH-WT and 7 IDH-mut gliomas were downloaded from European Nucleotide Archive (www.ebi.ac.uk/ena/) with data set ID EGAS00001005472. The gene expression matrix was downloaded from GEO with data set ID GSE151506. The clinical information of each sample as well as the cellular state assignment were obtained from the supplemental data of a previous publication (31). For AS analysis at sc resolution, the reads were aligned to the human genome reference hg19 using HISAT2 and processed through MISO to estimate PSI. In pseudobulk strategy, the read alignments from cells at the same cellular state in each patient were combined before PSI estimation with MISO. We performed hierarchical clustering analysis with PSI data after filtering out pseudobulks in which less than 50 of the 200 events were detected as well as events whose PSI were detected in less than 100 pseudo-bulks. The hierarchical clustering analysis were performed using Cluster v3.0 with the following setting: Similarity Metric, Correlation (uncentered); Clustering method, Centroid linkage. Another hierarchical clustering analysis were performed with the gene expression data of the 29 RBPs with the same setting in Cluster v3.0 software. Spearman’s rank correlation analysis was performed between RBP expression and the PSI of 108 filtered events which were detected in more than 200 cells. The correlation coefficient values from each RBP-event pair were used to perform hierarchical clustering analysis.

scRNA-Seq data of normal adult and fetal brain tissues from GSE67835 (23) were downloaded from European Nucleotide Archive (www.ebi.ac.uk/ena/). Reads were aligned to the human genome reference hg19 using HISAT2 and the read alignments from same cell types in each sample were combined before PSI estimation with MISO.

### CRISPR-mediated exon skipping.

We selected candidates from cassettes exons that are more included in samples with high AS scores to study their biological function in IDH-WT GBM/GSC cells. 8 SE or MXE events in genes *TPM3*, *MPZL1*, *TJP2*, *CSNK1D*, *MARK3*, *TPM1*, *PTPRF*, and *FYN* were selected by using the following criteria: (a) MeanDecreaseGini value greater than 0.5 or PSI difference between samples with high and low AS scores greater than 0.2; (b) moderately or highly expressed in IDH-WT GBM/GSC cells; (c) consistent splicing pattern between patient tumor samples and IDH-WT GBM/GSC cells; and (d) sequence around the splice sites is appropriate to design CRISPR-gRNA. To induce skipping of targeted exons, CRISPR-gRNAs were designed around the 5′/3′ splice sites or predicted branch point (http://nsclbio.jbnu.ac.kr/tools/RNABP/) of targeted exons, based on 2 criteria: (a) Cas9-mediated cleavage site (3–4 nucleotides upstream of the PAM sequence) less than 5 nucleotides away from the splice site or predicted branch point; (b) satisfy the “minimal off-target” criteria provided by the SYNTHEGO online tool (design.synthego.com). The target sequences are listed in Supplemental Table 7. The CRISPR backbone vector was used as the negative control. GSC/GBM cell lines, NHNPs, or NHAs were infected with exon-targeting CRISPR/Cas9 in a lentiviral vector. After validating exon-skipping by RT-PCR, heterogeneous cell populations were used in experiments to study the effects of exon skipping in specific cells.

### Generation of Genetically Engineered hiPSC Clones.

The iPSC clones, including WT, iPSC-T (*TP53*^–/–^), iPSC-C (*CDKN2A/2B*^–/–^), and iPSC-PCT (*PTEN*^–/–^, *CDKN2A/2B*^–/–^, *TERT*p^C228T/WT^), were recently described (33, 63). We introduced *IDH1*^R132H/WT^ or/and *ATRX*^–/–^ in iPSC-T and *MTAP*^–/–^ and/or EGFRvIII OE in iPSC-PCT using methods detailed below. *IDH1*-mutation was described to be an early genetic event (64). Thus, in the TIA model, we introduced the *IDH1* mutation before the *ATRX*-KO. On the other hand, EGFRvIII mutations can emerge as late and heterogenous events in GBM development (65). Therefore, in the PCTME model, we introduced EGFRvIII as the final modification.

We utilized a “CRISPR-single base editing” method (66) to generate a heterozygous IDH1-R132H mutation (CGT to CAT). The guide RNA with the sequence of 5′-GCAUGACGACCUAUGAUGAU-3′ was cloned into pLKO5.sgRNA.EFS.GFP (Addgene, no. 57822) (67). iPSCs were cotransfected with gRNA plasmid and pCMV-BE3 plasmid (Addgene, no. 73021) (68) by electroporation using the Neon Transfection System (Invitrogen, no. MPK5000) according to the manufacturer’s instructions. After 2 days, GFP^+^ cells were sorted and seeded into 96-well plates coated with matrigel at the density of 1 cell/well using FACSMelody 3-Laser Sorter (BD Biosciences) in mTeSR Plus medium supplemented with 10 μM Y-26732 (Stemcell Technologies) and 1 μM AG-120 (mutant IDH1 inhibitor). Y-26732 was removed when the medium was refreshed while AG-120 (1 μM) was continuously added during the whole culture process of iPSCs with *IDH1* mutation. Monoclonal cells were obtained after 3 weeks. Genomic DNA was extracted, and sanger sequencing was performed to screen genomic mutations using the primers listed in Supplemental Table 8. IDH1^R132H^ protein expression was confirmed by IB. AG-120 was removed 1 passage before the start of NPC induction. Cellular D-2-Hydroxyglutarate (D-2HG) level was detected in NPCs using a D-2HG Assay Kit (Abcam, ab211070) according to the manufacturer’s instructions.

We applied a CRISPR/Cas9-based gene KO method to induce *ATRX* or *MTAP* KO. The target gRNAs were designed using the SYNTHGO CRISPR Design Tool for Knockouts (https://design.synthego.com) and cloned into PX459 plasmid (Addgene, no. 62988) (69). iPSCs were transfected with PX459 plasmids with target gRNAs using Lipofectamine 2000 according to the manufacturer’s instructions. After 24 hours, the transfected cells were selected with 1 μg/mL puromycin for another 3 days. The survived cells were seeded into 96-well plates at the density of 1 cell/well. Monoclonal cells were obtained after 2 to 3 weeks. Genomic DNA was extracted, and Sanger sequencing was performed to screen genomic mutation. Immunoblotting was performed to measure the KO at the protein level.

Exogenous expression of EGFRvIII isoform was introduced using a lentiviral vector pLV-EF1a-EGFRvIII-IRES-Hyg (33). iPSCs or NPCs were infected with EGFRvIII-lentivirus packaged in transfected 293T cells and selected with 100 μg/mL hygromycin (Roche). The overexpression of EGFRvIII was validated by immunoblot (IB) and flow cytometry analysis.

### Animal studies.

Athymic mice (Ncr nu/nu) at 6 to 8 weeks of age were purchased from Taconic Farms.

For the tumorigenicity studies in intracranial xenograft models, luciferase reporter–labeled GSC1478 (2 × 10^4^ cells per mouse) or various iPSC-avatar NPC (5 × 10^5^ cells per mouse) suspensions were intracranially injected into the brain of individual athymic mice (5–6 mice/group) using the following coordinates from bregma: 2.5 mm lateral, 1.5 mm anterior, and 2.8 mm deep from the skull. Bioluminescence imaging (BLI) was conducted to monitor in vivo tumor growth using the SII Lago imaging system (Spectral Instruments Imaging). For survival analysis, mice were maintained until pathologic symptoms developed resulting from tumor burden or 120 days after brain transplantation.

To determine the antitumor effect of ASOs in an intracranial xenograft model of GBM, luciferase reporter–labeled GSC1478 cell suspension (2 × 10^4^ cells) was injected into the brain of individual mice, using the following coordinates from bregma: 2.5 mm lateral, 1.5 mm anterior, and 2.8 mm deep from the skull. Ten days after the GSC inoculation, tumor formation was confirmed by BLI, and mice were randomly divided into PTBP1-ASO and control-ASO groups (7–10 mice per group). Each mouse received sequential intratumoral injection (twice a week until the first mouse reached endpoint) of the ASOs (4 μg/mouse) mixed with in vivo-jetPEI reagent (Polyplus, 0.4 μL/mouse), which is a polymer-based reagent that condenses nucleic acid into stable nanoparticles. BLI was conducted to monitor in vivo tumor growth. For the survival analysis, mice were maintained until pathologic symptoms developed resulting from tumor burden.

### Statistics.

Statistical analyses were carried out using GraphPad Prism version 9 or Microsoft Excel 2022. All experiments were performed on biological replicates, and the exact sample size (*n*) for each experiment was reported in the appropriate figure legends and methods. Quantitative data are expressed as mean ± SD unless otherwise stated. For comparing 2 groups, the 2-tailed unpaired Student’s *t* test was used unless otherwise stated. For comparing multiple groups, 1-way ANOVA multiple comparisons with correction by controlling the FDR were used. For growth curve data, 2-way ANOVA with Geisser-Greenhouse correction were used. For survival curve data, Kaplan-Meier analysis was performed, and log-rank test was used to compare between groups. For limiting dilution assay, likelihood ratio test of single-hit model was used. Spearman correlation analysis was used to examine the correlation between 2 factors unless otherwise stated. *P* < 0.05 was considered significant.

### Study approval.

All experiments using animals were conducted under the IACUC-approved protocols at Northwestern University in accordance with NIH and institutional guidelines. Human Subjects Research protocols were approved by the Institutional Review Board at Northwestern University in accordance with guidelines by Declaration of Helsinki, NIH, and institutional Ethics Committee.

### Data availability.

The RNA-Seq data generated by this study have been deposited in the NCBI’s Gene Expression Omnibus database (GEO GSE212671). Values for all data points in graphs are reported in the Supporting Data Values file. No custom algorithms were used in this study.

Description of the experimental procedures related to cell lines and cell culture, glioma and normal brain tissue specimens, plasmids, differentiation of hiPSCs to NPCs, 3D organoid model of iPSC glioma avatars, RNA isolation, RT-PCR, IB, and immunoprecipitation assay, CRISPR-mediated gene KO, doxycycline-inducible shRNA-mediated gene knockdown, immunofluorescent and immunochemistry assay, flow cytometric detection, RNA-Seq in iPSC models, in vitro cell proliferation assays, in vitro limiting dilution assays, ceramide extraction and liquid-chromatography mass spectrometry analysis, synthesis and in vitro transfection of ASOs, and other bioinformatics analyses are detailed in the Supplemental Methods section.

## Author contributions

SYC, BH, and XS conceived the project. XS performed almost all the experiments and computational analyses, ML performed some of IHC experiments and analyses of splice site strength estimation and GC content estimation. MW performed some WB experiments during the revision process. CZ and WZ performed initial computational analysis and provided advice. SM, TK, and FBF established, and XS further developed hiPSC-derived glioma avatar models. HS performed lipidomic analysis of ceramides. SHSW and AH analyzed the expression of CERS5 isoforms from CPTAC-GBM proteome data set. CMH provided clinical glioma tumor samples. CH provided general advice on 3D organoid models. HS and SHSW helped in analyzed data of lipid metabolome and CPTAC proteome. XS, DT, SYC, and BH wrote the original draft of the mansucript. XS, DT, SYC, BH, TH, AG, RI, ML, XY, RW, MW, WZ, CMH, and FBF reviewed and edited the manuscript. SYC and BH supervised the project, were project administrators,and acquired funding for the project.

## Supplementary Material

Supplemental data

Unedited blot and gel images

Supplemental tables 1-10

Supporting data values

## Version 1 04/25/2024

In-Press Preview

## Version 2 06/03/2024

Electronic publication

## References

[1] Nicholson JG, Fine HA (2021). Diffuse glioma heterogeneity and its therapeutic implications. Cancer Discov.

[2] Louis DN (2021). The 2021 WHO classification of tumors of the central nervous system: a summary. Neuro Oncol.

[3] Ceccarelli M (2016). Molecular profiling reveals biologically discrete subsets and pathways of progression in diffuse glioma. Cell.

[4] Suva ML, Tirosh I (2020). The glioma stem cell model in the era of single-cell genomics. Cancer Cell.

[5] Tirosh I (2016). Single-cell RNA-seq supports a developmental hierarchy in human oligodendroglioma. Nature.

[6] Venteicher AS (2017). Decoupling genetics, lineages, and microenvironment in IDH-mutant gliomas by single-cell RNA-seq. Science.

[7] Neftel C (2019). An integrative model of cellular states, plasticity, and genetics for glioblastoma. Cell.

[8] Marasco LE, Kornblihtt AR (2023). The physiology of alternative splicing. Nat Rev Mol Cell Biol.

[9] Raj B, Blencowe BJ (2015). Alternative splicing in the mammalian nervous system: recent insights into mechanisms and functional roles. Neuron.

[10] Zhao L (2020). Comprehensive characterization of alternative mRNA splicing events in glioblastoma: implications for prognosis, molecular subtypes, and immune microenvironment remodeling. Front Oncol.

[11] Wang L (2021). The evolution of alternative splicing in glioblastoma under therapy. Genome Biol.

[12] Siddaway R (2022). Splicing is an alternate oncogenic pathway activation mechanism in glioma. Nat Commun.

[13] Wang LB (2021). Proteogenomic and metabolomic characterization of human glioblastoma. Cancer Cell.

[14] Zhao Z (2021). Chinese Glioma Genome Atlas (CGGA): a comprehensive resource with functional genomic data from Chinese glioma patients. Genomics Proteomics Bioinformatics.

[15] Huang T (2021). PRMT6 methylation of RCC1 regulates mitosis, tumorigenicity, and radiation response of glioblastoma stem cells. Mol Cell.

[16] Katz Y (2010). Analysis and design of RNA sequencing experiments for identifying isoform regulation. Nat Methods.

[17] Wang Q (2017). Tumor evolution of glioma-intrinsic gene expression subtypes associates with immunological changes in the microenvironment. Cancer Cell.

[18] Breiman L (2001). Decision trees and random forests. Am J Orthod Dentofacial Orthop.

[19] Shen S (2014). rMATS: robust and flexible detection of differential alternative splicing from replicate RNA-Seq data. Proc Natl Acad Sci U S A.

[20] Hyung D (2018). ASpedia: a comprehensive encyclopedia of human alternative splicing. Nucleic Acids Res.

[21] Ziller MJ (2018). Dissecting the functional consequences of de novo DNA methylation dynamics in human motor neuron differentiation and physiology. Cell Stem Cell.

[22] Jessa S (2019). Stalled developmental programs at the root of pediatric brain tumors. Nat Genet.

[23] Darmanis S (2015). A survey of human brain transcriptome diversity at the single cell level. Proc Natl Acad Sci U S A.

[24] Izaguirre DI (2012). PTBP1-dependent regulation of USP5 alternative RNA splicing plays a role in glioblastoma tumorigenesis. Mol Carcinog.

[25] Christofk HR (2008). The M2 splice isoform of pyruvate kinase is important for cancer metabolism and tumour growth. Nature.

[26] Hu J (2013). From the cover: neutralization of terminal differentiation in gliomagenesis. Proc Natl Acad Sci U S A.

[27] Song X (2019). SRSF3-regulated RNA alternative splicing promotes glioblastoma tumorigenicity by affecting multiple cellular processes. Cancer Res.

[28] Yamaguchi F (1994). Differential expression of two fibroblast growth factor-receptor genes is associated with malignant progression in human astrocytomas. Proc Natl Acad Sci U S A.

[29] Brignatz C (2009). Alternative splicing modulates autoinhibition and SH3 accessibility in the Src kinase Fyn. Mol Cell Biol.

[30] Mouneimne G (2012). Differential remodeling of actin cytoskeleton architecture by profilin isoforms leads to distinct effects on cell migration and invasion. Cancer Cell.

[31] Chaligne R (2021). Epigenetic encoding, heritability and plasticity of glioma transcriptional cell states. Nat Genet.

[32] Miki S (2022). TERT promoter C228T mutation in neural progenitors confers growth advantage following telomere shortening in vivo. Neuro Oncol.

[33] Koga T (2020). Longitudinal assessment of tumor development using cancer avatars derived from genetically engineered pluripotent stem cells. Nat Commun.

[34] Sundar SJ (2022). Three-dimensional organoid culture unveils resistance to clinical therapies in adult and pediatric glioblastoma. Transl Oncol.

[35] Levy M, Futerman AH (2010). Mammalian ceramide synthases. IUBMB Life.

[36] Grosch S (2012). Chain length-specific properties of ceramides. Prog Lipid Res.

[37] Sassa T (2016). Enzyme activities of the ceramide synthases CERS2-6 are regulated by phosphorylation in the C-terminal region. J Biol Chem.

[38] Zhao ZJ, Zhao R (1998). Purification and cloning of PZR, a binding protein and putative physiological substrate of tyrosine phosphatase SHP-2. J Biol Chem.

[39] Chen D (2019). MPZL1 promotes tumor cell proliferation and migration via activation of Src kinase in ovarian cancer. Oncol Rep.

[40] Feng J (2022). MPZL1 upregulation promotes tumor metastasis and correlates with unfavorable prognosis in non-small cell lung cancer. Carcinogenesis.

[41] Zhao R, Zhao ZJ (2000). Dissecting the interaction of SHP-2 with PZR, an immunoglobulin family protein containing immunoreceptor tyrosine-based inhibitory motifs. J Biol Chem.

[42] Zhang J (2015). Functions of Shp2 in cancer. J Cell Mol Med.

[43] Consortium EP (2012). An integrated encyclopedia of DNA elements in the human genome. Nature.

[44] Damianov A (2016). Rbfox proteins regulate splicing as part of a large multiprotein complex LASR. Cell.

[45] Qian H (2020). Reversing a model of Parkinson’s disease with in situ converted nigral neurons. Nature.

[46] Li Y (2019). Classification of glioma based on prognostic alternative splicing. BMC Med Genomics.

[47] Zeng Y (2020). Identification of prognostic signatures of alternative splicing in glioma. J Mol Neurosci.

[48] Kim JH (2021). SON drives oncogenic RNA splicing in glioblastoma by regulating PTBP1/PTBP2 switching and RBFOX2 activity. Nat Commun.

[49] Zhou X (2019). Splicing factor SRSF1 promotes gliomagenesis via oncogenic splice-switching of MYO1B. J Clin Invest.

[50] Kim HJ (2020). Genetic architectures and cell-of-origin in glioblastoma. Front Oncol.

[51] Robson JP (2018). Impaired neural stem cell expansion and hypersensitivity to epileptic seizures in mice lacking the EGFR in the brain. FEBS J.

[52] Ayuso-Sacido A (2010). Activated EGFR signaling increases proliferation, survival, and migration and blocks neuronal differentiation in post-natal neural stem cells. J Neurooncol.

[53] Figueroa ME (2010). Leukemic IDH1 and IDH2 mutations result in a hypermethylation phenotype, disrupt TET2 function, and impair hematopoietic differentiation. Cancer Cell.

[54] Lu C (2012). IDH mutation impairs histone demethylation and results in a block to cell differentiation. Nature.

[55] Modrek AS (2017). Low-grade astrocytoma mutations in IDH1, P53, and ATRX cooperate to block differentiation of human neural stem cells via repression of SOX2. Cell Rep.

[56] Rosiak K (2016). IDH1R132H in neural stem cells: differentiation impaired by increased apoptosis. PLoS One.

[57] Mendel M (2021). Splice site m^6^A methylation prevents binding of U2AF35 to inhibit RNA splicing. Cell.

[58] Zhou Z, Fu XD (2013). Regulation of splicing by SR proteins and SR protein-specific kinases. Chromosoma.

[59] Wang K (2022). PTBP1 knockdown promotes neural differentiation of glioblastoma cells through UNC5B receptor. Theranostics.

[60] De Silva MI (2023). Neuronal and tumourigenic boundaries of glioblastoma plasticity. Trends Cancer.

[61] Kim D (2015). HISAT: a fast spliced aligner with low memory requirements. Nat Methods.

[62] Zakharova G (2022). Reclassification of TCGA diffuse glioma profiles linked to transcriptomic, epigenetic, genomic and clinical data, according to the 2021 WHO CNS tumor classification. Int J Mol Sci.

[63] Miki S (2022). TERT promoter C228T mutation in neural progenitors confers growth advantage following telomere shortening in vivo. Neuro Oncol.

[64] Watanabe T (2009). IDH1 mutations are early events in the development of astrocytomas and oligodendrogliomas. Am J Pathol.

[65] Eskilsson E (2016). EGFRvIII mutations can emerge as late and heterogenous events in glioblastoma development and promote angiogenesis through Src activation. Neuro Oncol.

[66] Wei S (2018). Heterozygous IDH1^R132H/WT^ created by “single base editing” inhibits human astroglial cell growth by downregulating YAP. Oncogene.

[67] Heckl D (2014). Generation of mouse models of myeloid malignancy with combinatorial genetic lesions using CRISPR-Cas9 genome editing. Nat Biotechnol.

[68] Komor AC (2016). Programmable editing of a target base in genomic DNA without double-stranded DNA cleavage. Nature.

[69] Ran FA (2013). Genome engineering using the CRISPR-Cas9 system. Nat Protoc.

