# Supplementary material for: RNA splicing analysis deciphers developmental hierarchies and reveals therapeutic targets in adult glioma
Source: J Clin Invest. 2024 Apr 25;134(11):e173789. doi: 10.1172/JCI173789 (PMC11142752; doi:10.1172/JCI173789)
Supplement: Supplemental data [file jci-134-173789-s100.pdf]

SUPPLEMENTAL INFORMATION

Document 1: Supplemental Figure 1-7 and Supplemental Methods.

Document 2: Supplemental Tables 1-10.

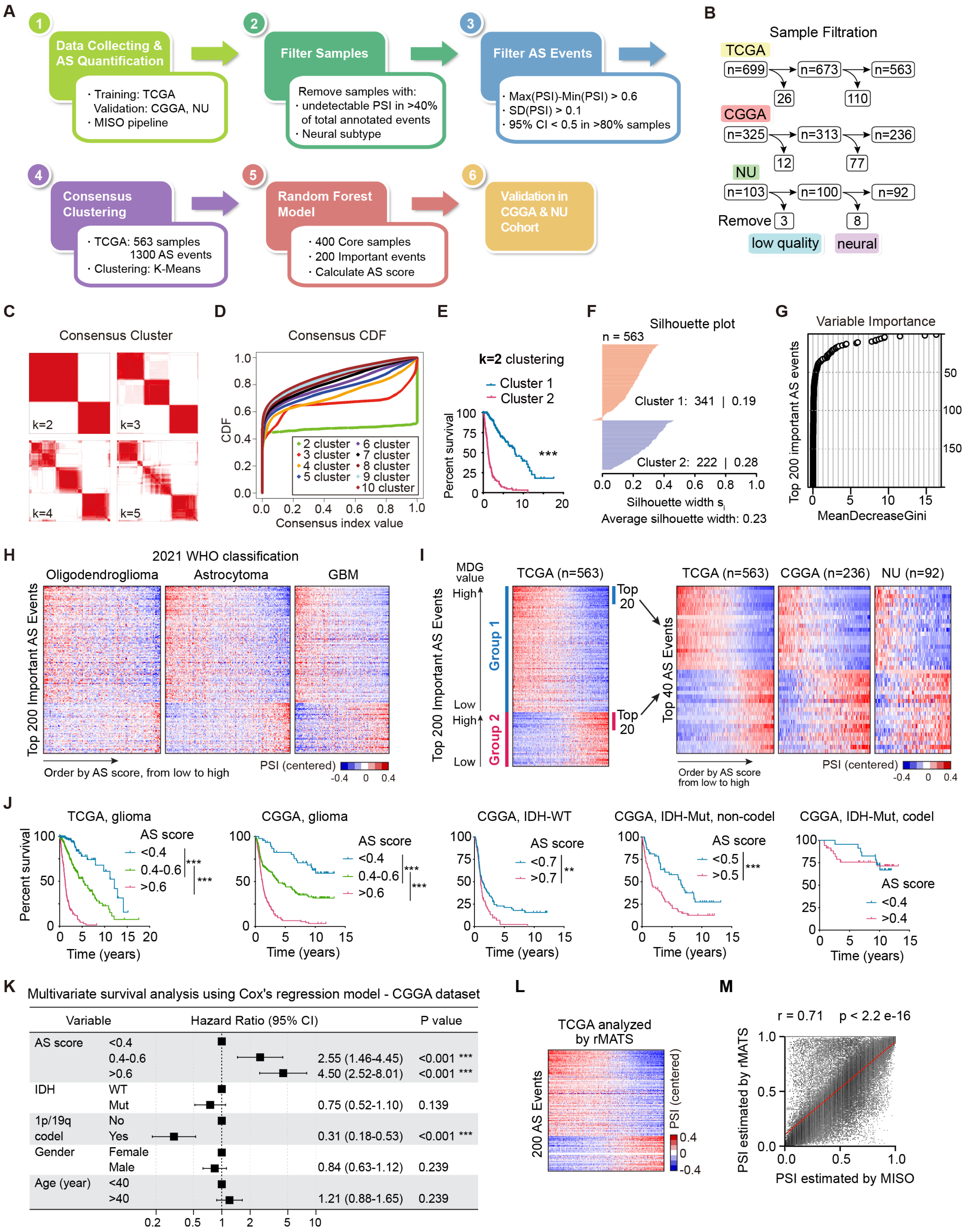
A
1 Data Collecting & AS Quantification
· Training: TCGA Validation: CGGA, NU
· MISO pipeline
2 Filter Samples
Remove samples with:
· undetectable PSI in >40% of total annotated events
· Neural subtype
3 Filter AS Events
· Max(PSI)-Min(PSI) > 0.6
· SD(PSI) > 0.1
· 95% CI < 0.5 in >80% samples
4 Consensus Clustering
· TCGA: 563 samples 1300 AS events
· Clustering: K-Means
5 Random Forest Model
· 400 Core samples
· 200 Important events
· Calculate AS score
6 Validation in CGGA & NU Cohort
B
Sample Filtration
TCGA
n=699
n=673
n=563
26
110
CGGA
n=325
n=313
n=236
12
77
NU
n=103
n=100
n=92
Remove
3
8
low quality
neural
C
Consensus Cluster
k=2
k=3
k=4
k=5
D
Consensus CDF
CDF
Consensus index value
2 cluster
3 cluster
4 cluster
5 cluster
6 cluster
7 cluster
8 cluster
9 cluster
10 cluster
E
k=2 clustering
Cluster 1
Cluster 2
Percent survival
***
F
Silhouette plot
n = 563
Cluster 1: 341 | 0.19
Cluster 2: 222 | 0.28
Silhouette width s_i
Average silhouette width: 0.23
G
Variable Importance
Top 200 important AS events
MeanDecreaseGini
H
2021 WHO classification
Oligodendroglioma
Astrocytoma
GBM
Top 200 Important AS Events
Order by AS score, from low to high
PSI (centered)
I
MDG value
High
Low
TCGA (n=563)
Group 1
Group 2
Top 20
Top 200 Important AS Events
Top 40 AS Events
TCGA (n=563)
CGGA (n=236)
NU (n=92)
Order by AS score from low to high
PSI (centered)
J
TCGA, glioma
CGGA, glioma
CGGA, IDH-WT
CGGA, IDH-Mut, non-codel
CGGA, IDH-Mut, codel
AS score
<0.4
0.4-0.6
>0.6
<0.7
>0.7
<0.5
>0.5
>0.4
Percent survival
Time (years)
K
Multivariate survival analysis using Cox's regression model - CGGA dataset
Variable
Hazard Ratio (95% CI)
P value
AS score <0.4
0.4-0.6 2.55 (1.46-4.45) <0.001 ***
>0.6 4.50 (2.52-8.01) <0.001 ***
IDH WT
Mut 0.75 (0.52-1.10) 0.139
1p/19q codel No
Yes 0.31 (0.18-0.53) <0.001 ***
Gender Female
Male 0.84 (0.63-1.12) 0.239
Age (year) <40
>40 1.21 (0.88-1.65) 0.239
L
TCGA analyzed by rMATS
200 AS Events
PSI (centered)
M
r = 0.71 p < 2.2 e-16
PSI estimated by rMATS
PSI estimated by MISO

Supplemental Figure 1. Unsupervised splicing analysis in glioma bulks revealed a prognostic AS signature that links neural lineage differentiation program.

(**A**) Workflow to define AS-based signature in gliomas.

(**B**) Information of sample size and filtration of all three datasets.

(**C**) Consensus clustering matrix of TCGA samples for k = 2 to k = 5.

(**D**) Consensus clustering cumulative distribution function (CDF) for k = 2 to k = 10.

(**E**) Survival curves of clusters for k = 2.

(**F**) Silhouette plot for identification of the most representative samples.

(**G**) Mean Decrease Gini plot for identification of top 200 important AS events.

(**H**) Heatmaps showing the PSI values of the 200 AS events across TCGA gliomas divided by the 2021 WHO CNS5 classification. Samples were ordered based on their AS scores.

(**I**) Heatmaps showing the PSI values of the 40 AS events across three glioma datasets.

(**J**) Kaplan-Meier analyses in glioma subtypes from TCGA or CGGA datasets grouped by AS score.

(**K**) Multivariate cox regression analysis for overall survival in CGGA glioma samples. HR: hazard ratio; CI: confidence interval. LGG, lower-grade glioma.

(**L**) Heatmaps showing the PSI values of the 200 events estimated by rMATS algorithms.

(**M**) Pearson correlation analysis between PSI values estimated by MISO and rMATS algorithms.

Data were analyzed using log-rank test in E and G. **, p<0.01; ***, p<0.001.

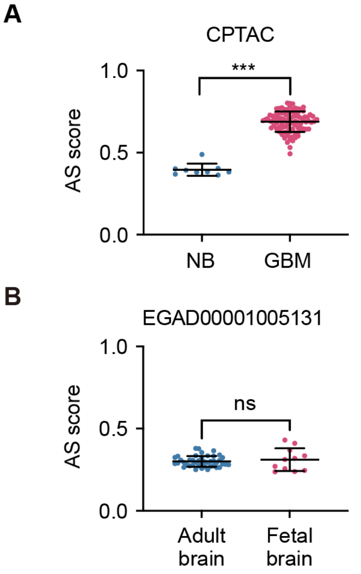

C

AS events	AS Type	Isoform-specific function
USP5 A5SS	Isoform 1: longer E15<br>Isoform 2: shorter E15	Isoform 1<br>inhibits GBM growth
PKM MXE	Isoform 1: E9+<br>Isoform 2: E9' +	Isoform 2<br>promotes tumor glycolysis
TPM1 MXE	Isoform 1: E6'+<br>Isoform 2: E6 +	Isoform 1<br>inhibits GBM growth
NDE1 MXE	Isoform 1: E9'+<br>Isoform 2: E9 +	Isoform 2 regulates<br>spindle formation and<br>promote GBM growth
FGFR1 SE	Isoform 1: E3+<br>Isoform 2: E3 -	Isoform 2 has<br>higher affinity to FGF1
FYN MXE	Isoform 1: E9+<br>Isoform 2: E9' +	Isoform 2 has<br>enhanced kinase activity
PFN2 A3SS	Isoform 1: longer E3<br>Isoform 2: shorter E3	Isoform 1 inhibited<br>breast cancer cell invasion

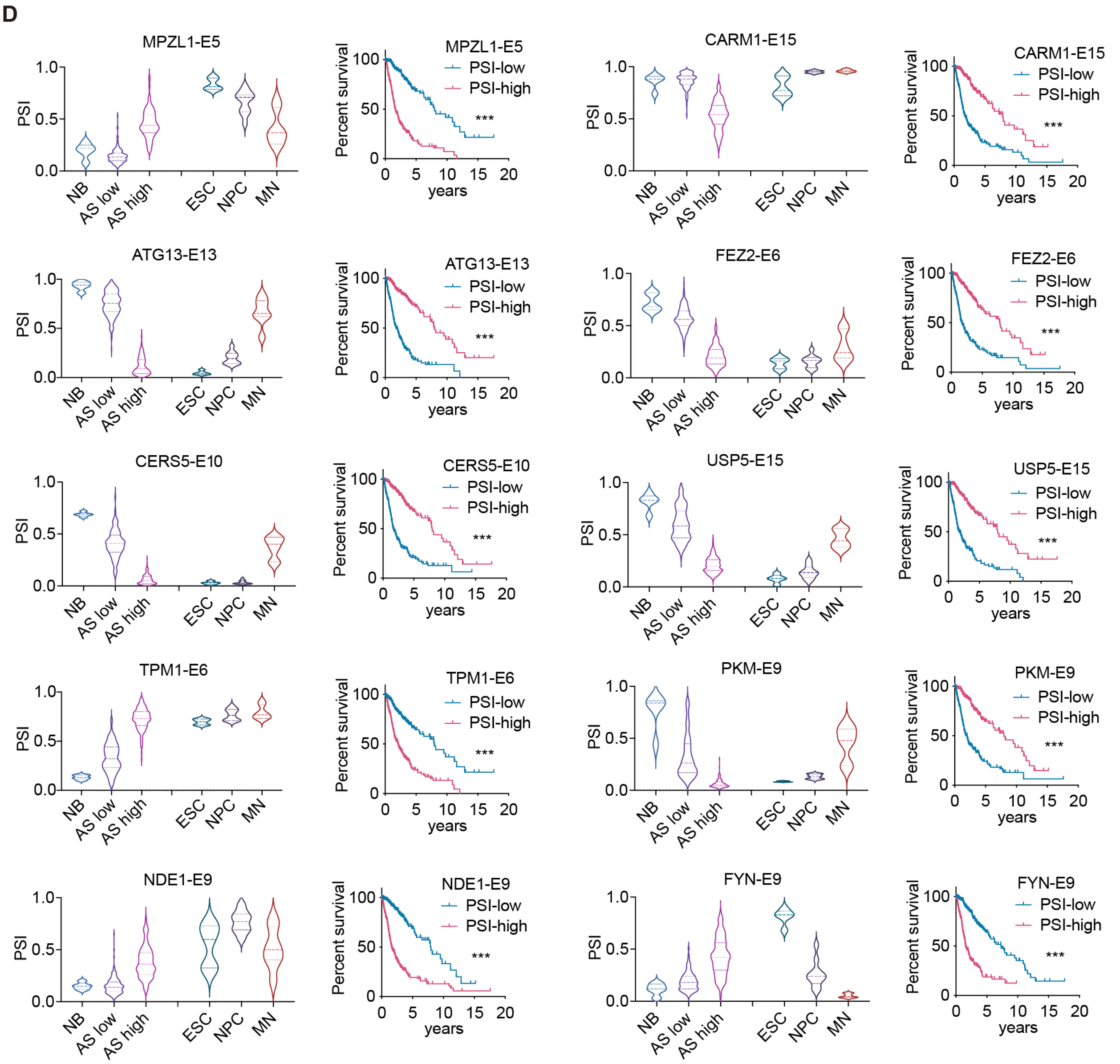

Supplemental Figure 2. An overview of the 200 events.

(**A**) AS scores in normal brain (NB) and GBM tissues from CPTAC dataset.

(**B**) AS scores in adult brain and fetal brain tissue from EGAD00001005131 datasets.

(**C**) Published functional impact of AS events on biological functions and/or tumorigenic properties from the 200 AS events.

(**D**) Percent spliced in (PSI) distribution and survival analysis of each indicated AS events from RNA-seq data of TCGA and GSE90553.

Data were analyzed using two-tailed unpaired t-test in A and B, and log-rank test in D. Median and quartiles are indicated on violin plots in D. Data in other quantitative panels are presented as mean ± SD. ***, $p<0.001$; ns, not significant.

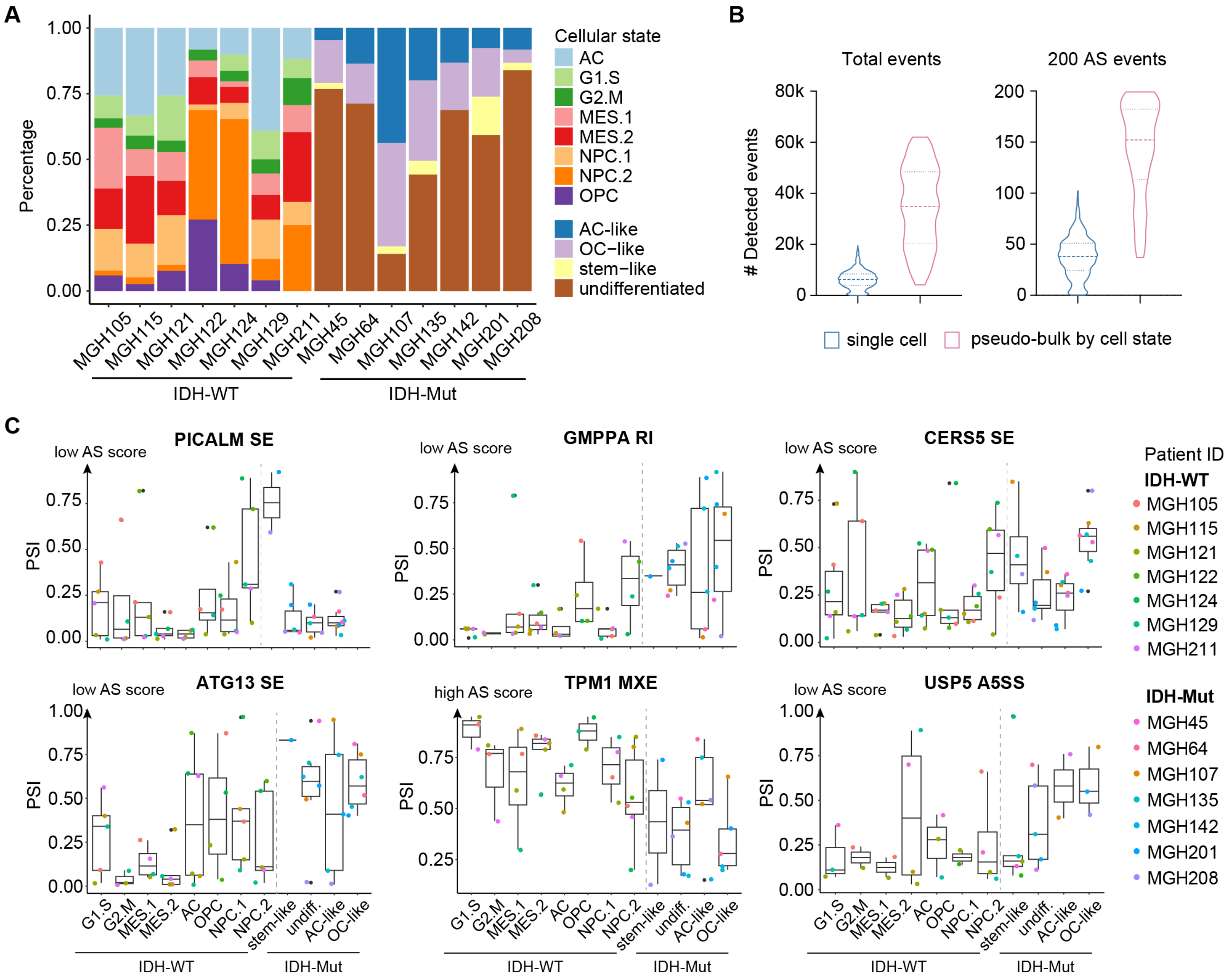

Supplemental Figure 3. Intra-tumoral AS heterogeneity is associates with the developmental hierarchy in glioma.

(**A**) The proportion of cells in each cellular state for each patient in GSE151506 scRNA-seq dataset of gliomas.

(**B**) Number of detected events in AS estimation at single-cell resolution or using a pseudo-bulk strategy.

(**C**) Box-plots showing the PSI distribution of representative AS events in each cell states. The box representing the interquartile range of the data, the line within the box representing the median, and the whiskers extending to the most extreme data points within 1.5 times the IQR. Individual data points beyond this range are shown as dots. The color of the dots represents the patient as indicated.

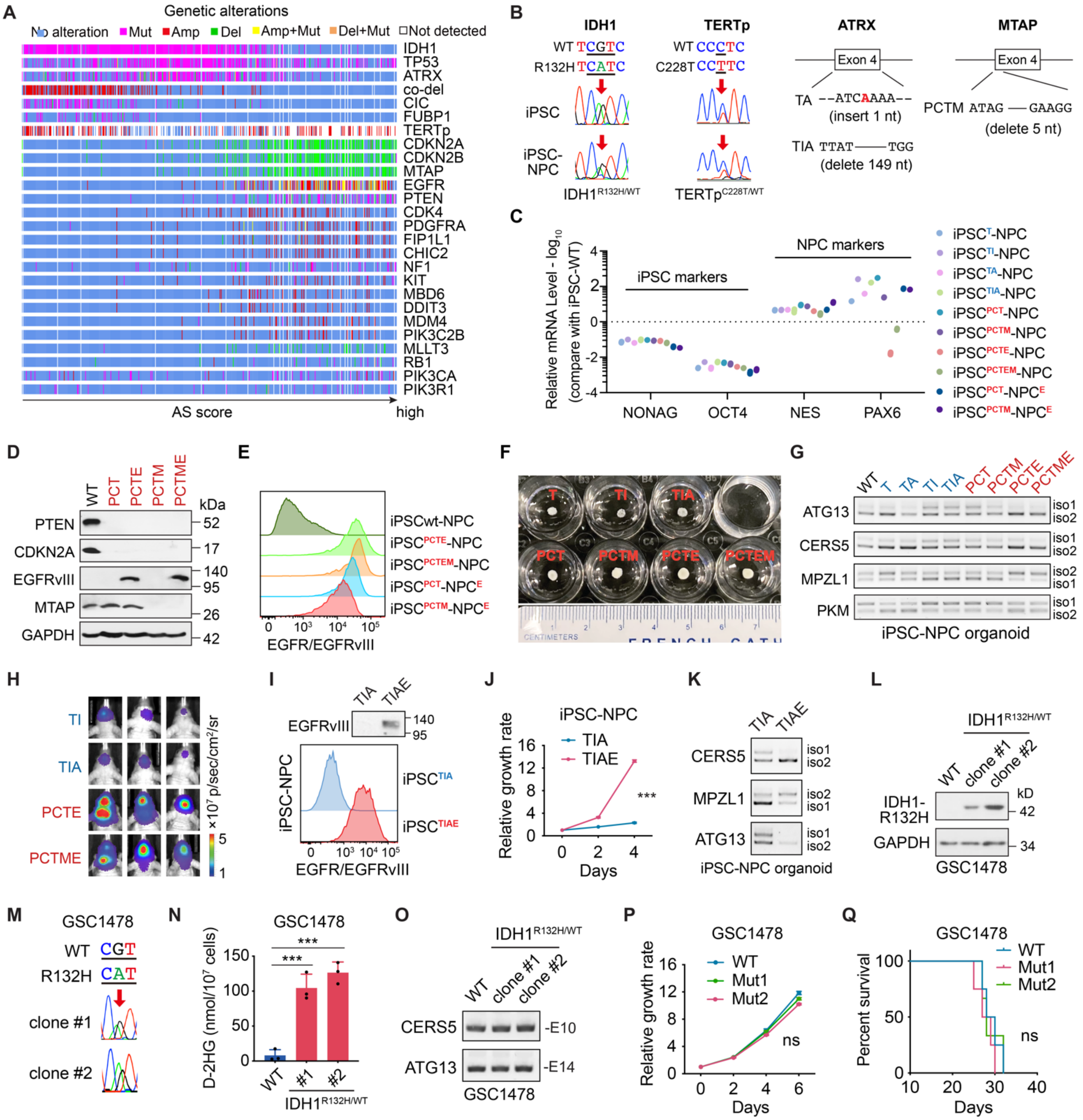

Supplemental Figure 4. Glioma driver mutations modulate AS landscape and neural developmental program.

(**A**) Mutational landscape of somatic alterations in TCGA glioma samples order by AS scores, from low to high.

(**B**) Sanger sequencing results showing heterozygous mutations of *IDH1* and *TERT* promoter (TERTp), and indel mutations in *ATRX* and *MTAP* in edited iPSCs.

(**C**) qRT-PCR analysis of indicated markers in edited iPSC-derived NPCs.

(**D**) IB analysis in indicated NPCs. GAPDH was a loading control.

(**E**) Flow cytometry analysis with an anti-EGFR monoclonal antibody that recognize both WT and vIII isoform of EGFR proteins in edited NPCs.

(**F**) Photos of organoids with indicated genotypes after a 14-day culture.

(**G**) RT-PCR analysis of indicated AS events in edited iPSC-derived NPC organoids.

(**H**) Representative bioluminescent images (BLI) of intracranial xenografts from edited NPCs at 30 days post implantation.

(**I-K**) Effect of EGFRvIII on cell proliferation and AS in iPSC-TIA. **I**, IB (upper) and flow cytometry (lower) analysis of EGFRvIII overexpression. **J**, In vitro cell proliferation assay. N = 4. **K**, RT-PCR analysis of indicated AS events.

(**L-Q**) Effect of IDH1 mut on AS and tumor growth in GSC1478. **L**, IB analysis of IDH1 mut proteins. **M**, Sanger sequencing. **N**, Detection of intracellular D-2HG. N = 3. **O**, RT-PCR analysis of indicated AS events. **P**, In vitro cell proliferation assay. N = 4. **Q**, Kaplan-Meir analysis of mice injected with GSC1478 IDH-WT or mut cells. N = 3-4.

Data were analyzed using two-way ANOVA in J and P, and log-rank test in Q. ***, $p<0.001$. ns, not significant.

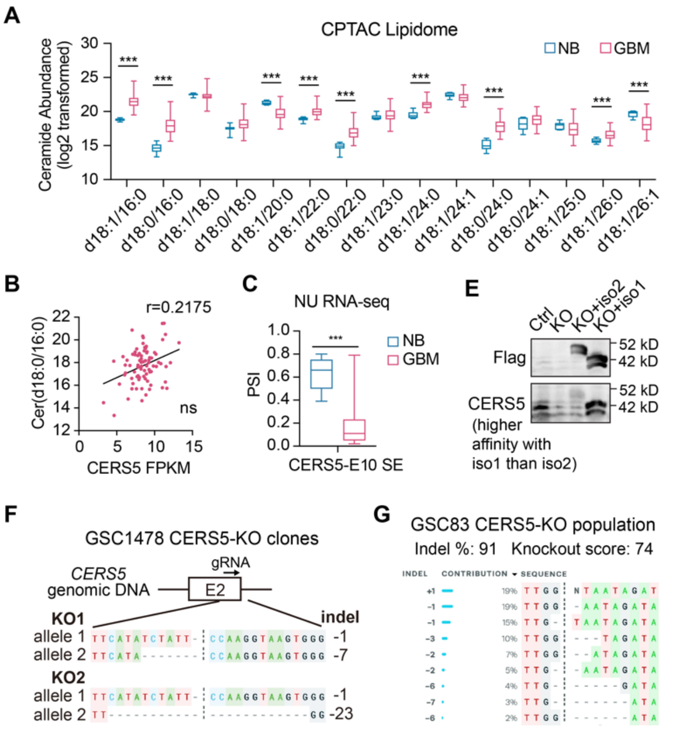

D

AS events	AS Type	Amino acid variations
CERS5 SE	iso 1: E10+<br>iso 2: E10 -	Different C-terminus:<br>iso 1: TCPGRIRPCTLHSFLTSFLFSMPGGSWTVSSLA CIEG<br>iso 2: SKDDRSDVESSSEEEDVTTCTKSPCDSSSSNG ANRVNGHMGGSYWAEE
CSNK1D SE	iso 1: E9 -<br>iso 2: E9 +	Different C-terminus:<br>iso 1: IPGRVASSGLQSVVHR<br>iso 2: NSIPFEHHGK
MPZL1 SE	iso 1: E5 -<br>iso 2: E5 +	Different C-terminus:<br>iso 1: AQSYMHS<br>iso 2: CSTSESLSPVKQAPRKSPSDTEGLVKSLPSGSH QGPVIYAQLDHSGGHHSDKINKSESVVYADIRKN
FYN MXE	iso 1: E9 +<br>iso 2: E9' +	E9/E9'-encoded peptide (234-287/573 aa)<br>iso 1: RAAGLCCRLVVPCHKGMPRLTDLSVKTKDVWE IPRESLQLIKRLGNGQFGEVWM<br>iso 2: KADGLCFNLTVIASSCTPQTSGLAKDAWEVARR SLCLEKKLGQGCFAEVWL
TPM1 MXE	iso 1: E6'+<br>iso 2: E6 +	E6/E6'-encoded peptide (153-175/248 aa, tropomyosin)<br>iso 1: KCAELEEELKTVTNNLKSLEAQAE<br>iso 2: EEKVAHAKEENLSMHQMLDQTLLELNNM
TPM3 MXE	iso 1: E5'+<br>iso 2: E5 +	E5/E5'-encoded peptide (153-175/248 aa, tropomyosin)<br>iso 1: KCSELEEELKNVTNNLKSLEAQAE<br>iso 2: QVRQLEEQLRIMDQTLKALMAAED
PTPRF SE	iso 1: E12 -<br>iso 2: E12 +	E12-encoded peptide (605-706/1898 aa, fn3 domain)<br>TAQSTPSAPPQKVMCVSMGSTTVRVSWVPPPADSRN GVITQYSVAYEAVDGEDRGRHVVDGISREHSSWDLVG LEKWTEYRVWVRAHTDVGPGPESSPVLVRTDED
MARK3 MXE	iso 1: E16' +<br>iso 2: E16 +	E16/E16'-encoded peptide (616-707/744 aa)<br>iso 1: LPTEYERNGRYEGS<br>iso 2: NMSFRFIK
TJP2 SE	iso 1: E20 -<br>iso 2: E20 +	E20-encoded peptide (961-997/1190 aa)<br>SIRKPSPEPRAQMRRAASSDQLRDNSPPPAFKPEPPK

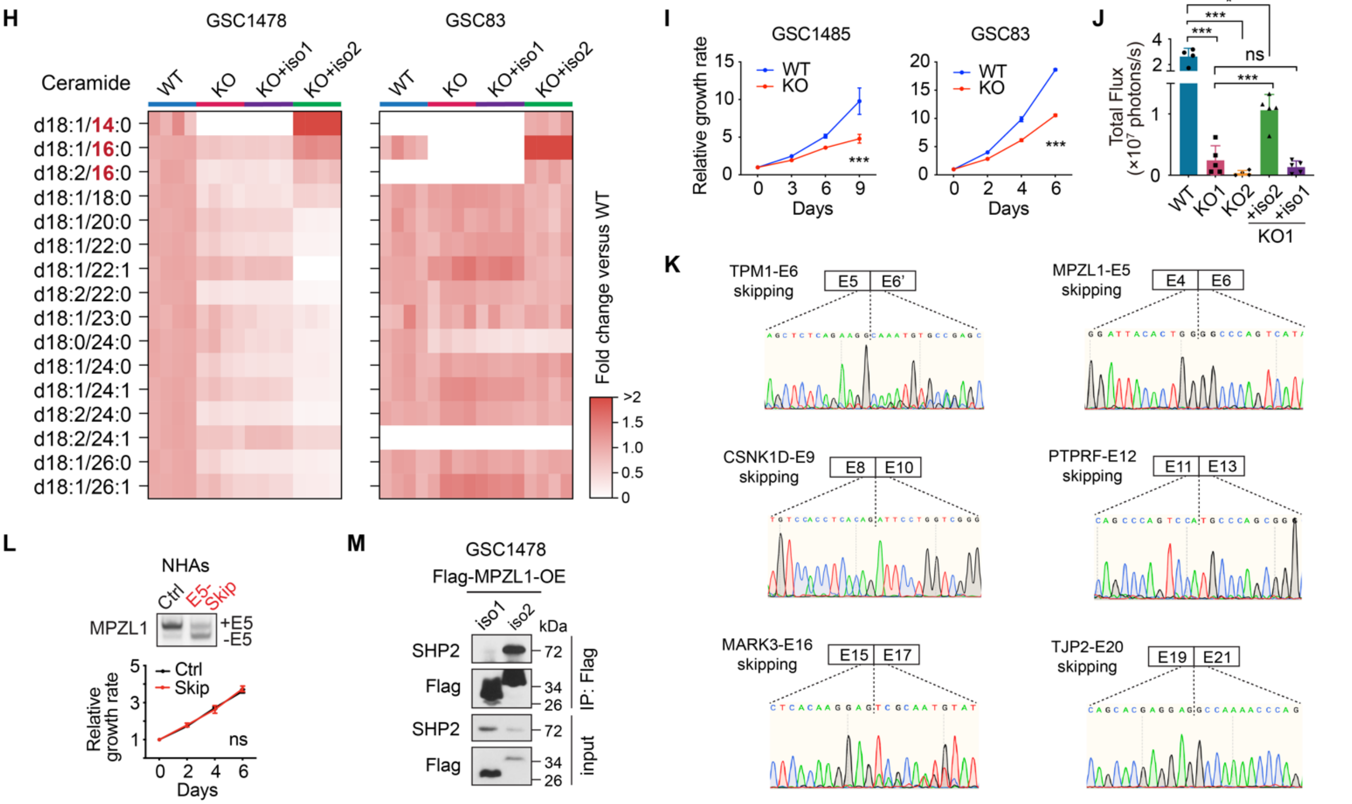

Supplemental Figure 5. AS of CERS5 and MPZL1 influences the oncogenic potential of glioma cells.

(**A**) Abundance of ceramide with specific acyl chain lengths between normal brain and IDH-WT GBM tissues analyzed from the CPTAC-GBM lipidome dataset.

(**B**) Spearman correlation analysis between C16-ceramide and PSI of CERS5 gene expression.

(**C**) PSI value of CERS5-E10-SE event between normal brain (NB) and GBM tissues analyzed from NU RNA-seq dataset.

(**D**) Detailed information of AS types and consequent impact on amino acid variations of nine selected candidates. Phosphorylation sites curated at PhosphoSitePlus were highlighted with red color.

(**E**) IB analysis in GSC1478 CERS5-KO, -KO+iso1, -KO+iso2, or a control cells.

(**F-G**) Sanger sequence results in two GSC1478 CERS5-KO clones (**F**) and GSC83 CERS5-KO population (**G**).

(**H**) Ceramide profile in GSCs with indicated treatment. N = 4.

(**I**) Effects of CERS5-KO on cell proliferation of indicated GSCs. N = 4.

(**J**) Quantification of bioluminescent signals of brain xenografts from GSC1478 with CERS5-KO or indicated rescue. N = 4-5.

(**K**) Sanger sequencing results showing the skipping of specific exons using CRISPR-based method.

(**L**) Effect of CRISPR-mediated exon skipping of *MPZL1*-E5 (upper, RT-PCR) on the cell viability (lower, cell proliferation) of NHAs. N=4.

(**M**) IP-IB with indicated antibodies in GSC1478 cells overexpressed with Flag-tagged MPZL1 isoforms.

Data were analyzed using two-tailed unpaired t-test in A, C, L, and J, and two-way ANOVA in I.

*, $p<0.05$; ***, $p<0.001$; ns, not significant.

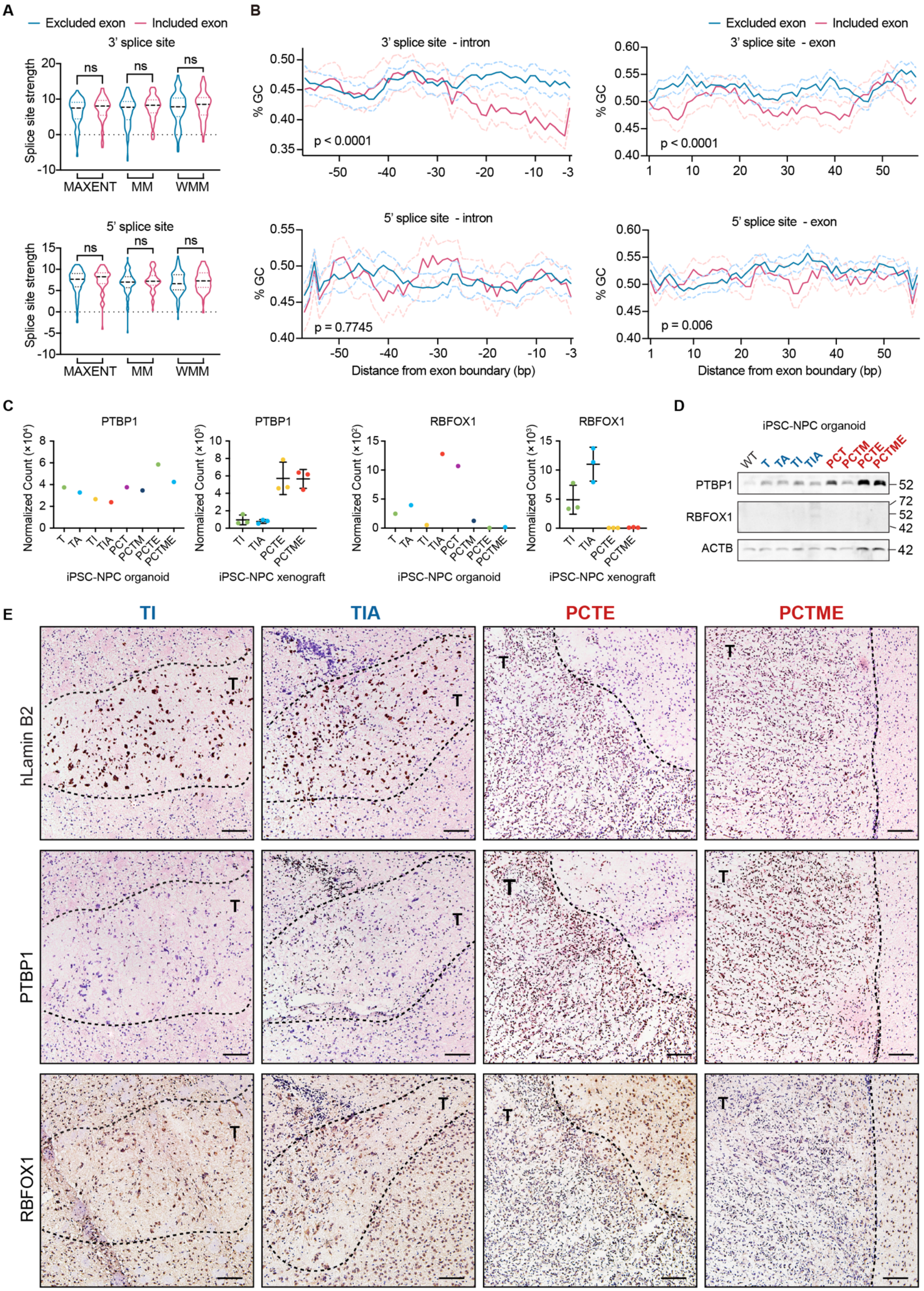
A
Excluded exon
Included exon
3' splice site
Splice site strength
ns
MAXENT
MM
WMM
5' splice site
B
3' splice site - intron
% GC
p < 0.0001
3' splice site - exon
5' splice site - intron
p = 0.7745
Distance from exon boundary (bp)
5' splice site - exon
p = 0.006
C
PTBP1
Normalized Count
iPSC-NPC organoid
iPSC-NPC xenograft
RBFOX1
T
TA
TI
TIA
PCT
PCTM
PCTE
PCTME
D
iPSC-NPC organoid
WT
ACTB
E
hLamin B2

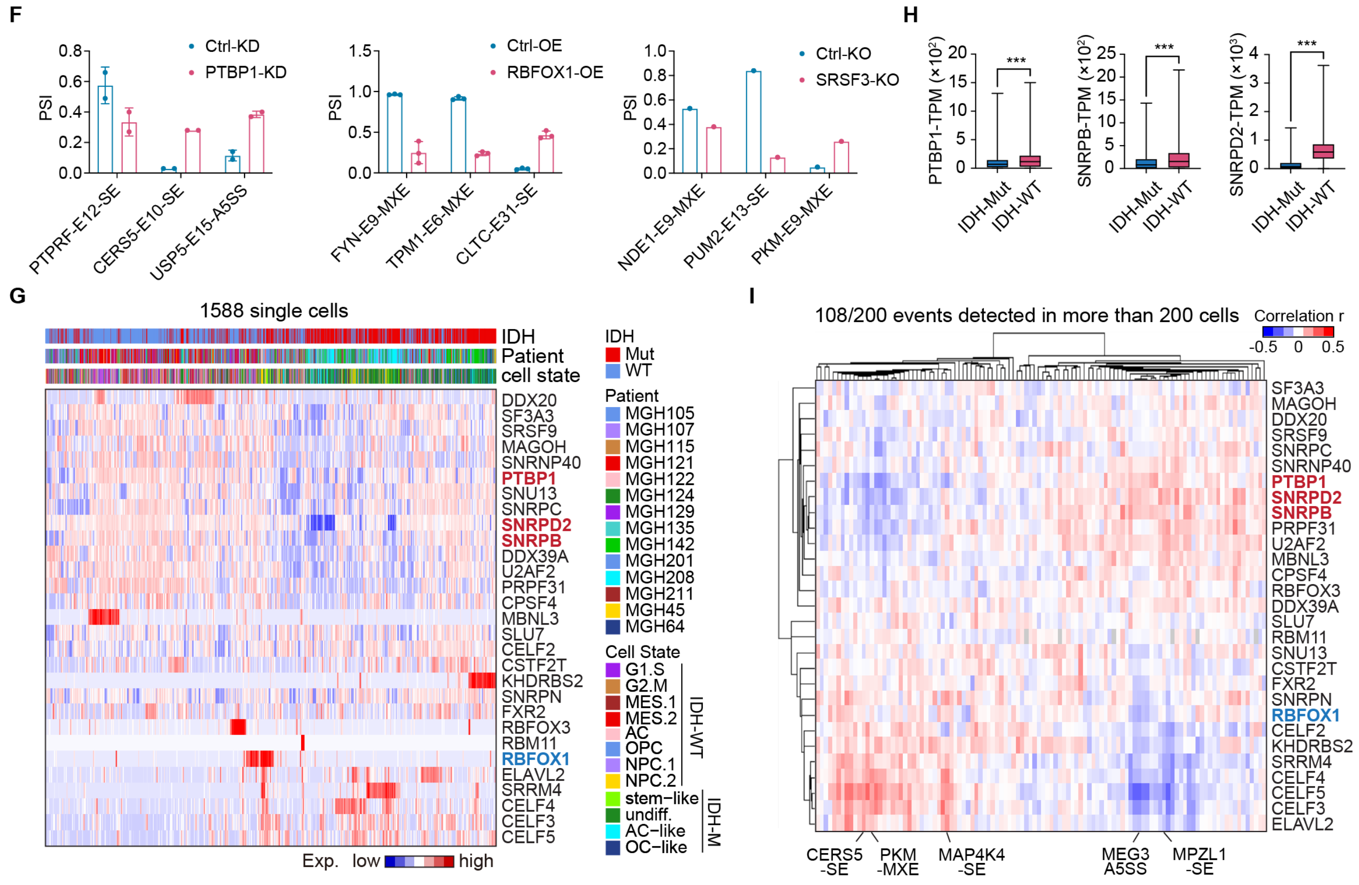

Supplemental Figure 6. A group of splicing-regulating RBPs modulate the AS landscape in glioma.

(**A**) Splice site strength evaluated by maximum entropy (MAXENT), first-order Markov model (MM), or weight matrix model (WMM) at the 3′ or 5′ splice sites of the exons from 200 events. Median and quartiles are indicated on violin plots.

(**B**) A sliding window (8 bp) plot of GC content in regions around 3' or 5' splice sites of the exons from 200 events. The dash lines correspond to the 95% confidence intervals.

(**C-E**) PTBP1 and RBFOX1 expression in edited iPSC-NPC organoids and xenografts analyzed from RNA-seq data (**C**), IB (**D**), and IHC analysis (**E**), Scale, 100 μm. T: tumor area.

(**F**) PSI changes of indicated AS events that are affected by PTBP1-KD, RBFOX1-OE, and SRSF3-KO analyzed from public RNA-seq datasets.

(**G**) Hierarchical clustering analysis with the gene expression data of RBPs in each glioma single cells analyzed from glioma scRNA-seq dataset, GSE151506.

(**H**) Box-plots showing the expression of indicated RBPs between IDH1-mut and WT glioma cells analyzed from glioma scRNA-seq dataset. The box represents the interquartile range, the line within the box represents the median, and the whiskers extending to the maximum and minimum values.

(**I**) Spearman correlation analysis between RBP expression and the PSI of 108 events that are from the 200 events and were detected in more than 200 single cells.

Data were analyzed using two-tailed unpaired t-test in A and H. ***, $p<0.001$; ns, not significant.

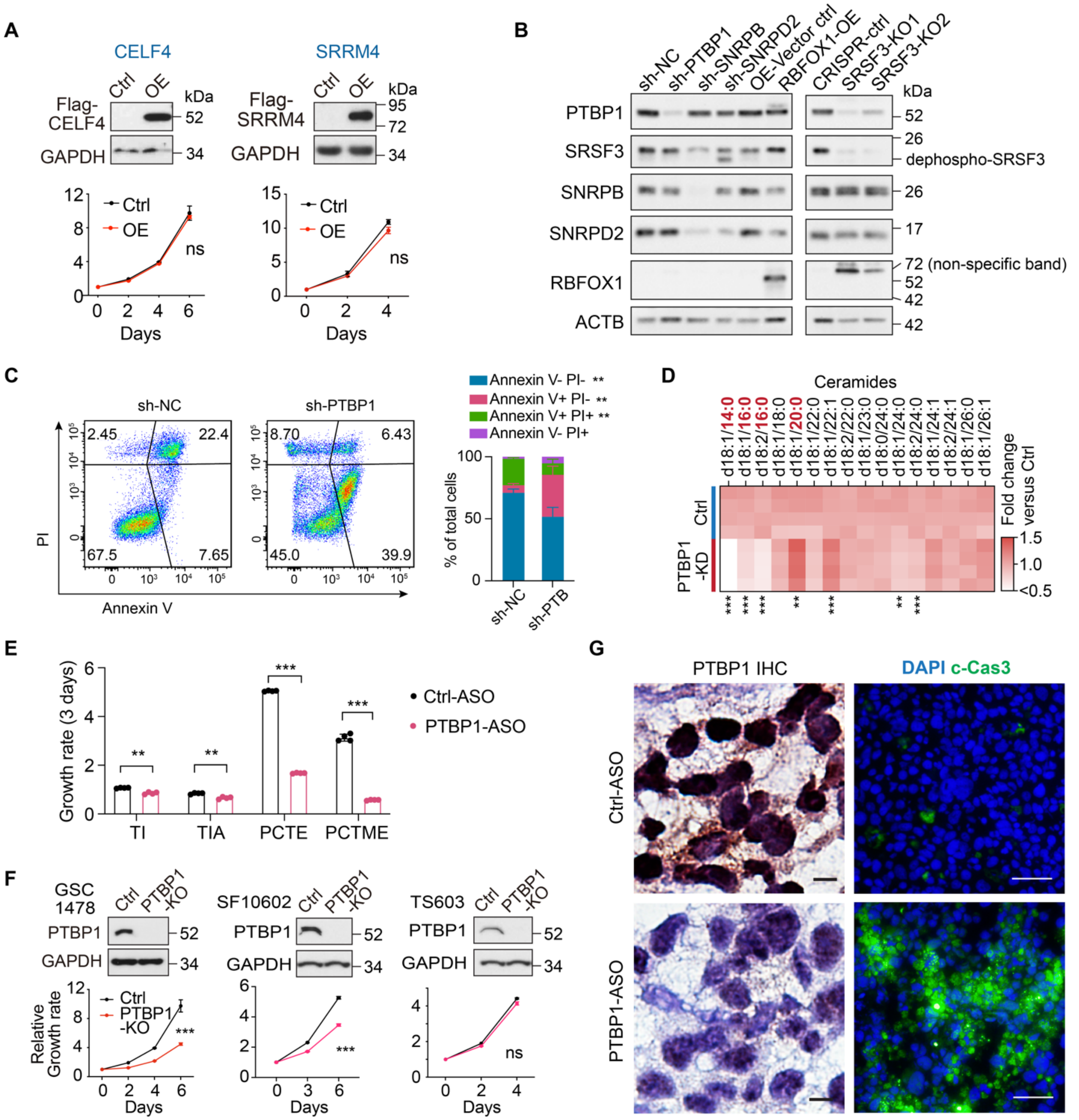

Supplemental Figure 7. Therapeutic effect of PTBP1-targeting ASO in vivo.

(A) Effect of overexpression (OE) of indicated RBPs on cell proliferation of GSC1478 (CELF4) or U87 (SRRM4). Upper, IB. Lower, cell proliferation curve. N = 4.

(B) IB analysis of RBP expression in GSC1478 cells with indicated treatment.

(**C**) Flow cytometry analysis of cell apoptosis in GSC1478 treated with PTBP1 shRNA (sh-PTBP1) or a negative control (sh-NC). N = 4.

(**D**) Ceramide profile in GSC1478 treated with sh-PTBP1 or sh-NC.

(**E**) Effects of PTBP1-targeting ASO1 on cell proliferation in edited iPSC-derived NPCs with indicated mutations. N = 4.

(**F**) Effect of PTBP1-KO on cell proliferation of indicated cells. Upper, IB. Lower, cell proliferation curve. N = 3-4.

(**G**) Representative images of IHC staining of PTBP1 (scale bar, 10 μm) and immunofluorescent analysis of cleaved-Caspase 3 (c-Cas3, scale bar, 40 μm) in GSC1478-derived intracranial xenografts treated with PTBP1-ASO or control-ASO. N = 3.

Data were analyzed using two-way ANOVA in A and F, and two-tailed unpaired t-test in C-E. **, $p<0.01$; ***, $p<0.001$; ns, not significant.

Supplemental Methods

Cell Lines and Cell Culture

Human HEK293T cells (ATCC) and glioma U87 cells (ATCC) were cultured in DMEM (Thermo Fisher Scientific, 11995-065) supplemented with 10% FBS (Thermo Fisher Scientific, 10437028) and 1% penicillin and streptomycin (Thermo Fisher Scientific, 15140122). Normal human astrocytes (NHA, Clonetics, CC-2565) were cultured in the Astrocyte Basal Medium (Lonza, CC-3187) supplemented with Astrocyte Growth Medium BulletKit (Lonza, CC-4123). Human Neural Progenitor Cells (NHNPs, Lonza, PT-2599) were cultured in the Neural Progenitor Maintenance Medium (Lonza, CC-3209). Patient-derived IDH-WT GSCs, GSC83, GSC46, GSC1478, and GSC1485 were previously characterized (1-3) and cultured in GSC medium, which includes DMEM/F12 medium (Thermo Fisher Scientific, 11320-033), 2% B27 supplement (Thermo Fisher Scientific, 17504-044), 1 × antibiotic-antimycotic (Thermo Fisher Scientific, 15240062), 5 mg/mL heparin (Sigma-Aldrich, 9041-08-1), 20 ng/mL EGF (Peprotech, 100-15R), and 20 ng/mL bFGF (Peprotech, 100-18B). Patient-derived IDH-mut GSCs, SF10602 and TS603, kindly provided by Dr. Joseph F. Costello at University of California, San Francisco, San Francisco, California, USA (4) and Dr. Chunzhang Yang at National Cancer Institute, Bethesda, Maryland, USA (5) respectively, were cultured in N2B27 medium (50% DMEM/F12 and 50% Neurobasal-A medium supplemented with GlutaMAX, 1 × N-2 supplement, 1 × B-27 supplement, 1 × antibiotic-antimycotic, 150 μM ascorbic acid) plus 5 mg/mL heparin (Sigma-Aldrich, 9041-08-1), 20 ng/mL EGF (Peprotech, 100-15R), and 20 ng/mL bFGF (Peprotech, 100-18B). The pathological and genetic information of GSCs were included in Supplemental Table 9. All the glioma cell lines were authenticated by short tandem repeat analysis at IDEXX BioAnalytics, Texas Tech

University Health Sciences Center (Lubbock, TX), or Northwestern University NUSeq core facility. All cell lines were tested negative for Mycoplasma using VenorGeM Mycoplasma Detection Kit (Sigma-Aldrich, MP0025). The latest authentication and Mycoplasma testing were in December 2022. All cell lines were cultured less than 20 passages prior to use.

Human iPS cells with the background of iPSC12 were obtained as recently reported (6, 7). iPS cells were cultured on plates coated with Matrigel hESC-Qualified Matrix (Corning) in mTeSR Plus medium (Stemcell Technologies). iPSC-derived NPCs were cultured on matrigel-coated plates in N2B27 medium (50% DMEM/F12 and 50% Neurobasal-A medium supplemented with GlutaMAX, 1 × N-2 supplement, 1 × B-27 supplement, 1 × antibiotic-antimycotic, 150 μM ascorbic acid) plus 3 μM CHIR99021 (DNSK International) and 1 μM SAG (DNSK International).

Glioma and Normal Brain Tissue Specimens

Human Subjects Research protocols were approved by the Institutional Review Board at Northwestern University in accordance with guidelines by Declaration of Helsinki, NIH, and institutional Ethics Committee. Fresh and snap-frozen tissue fragments were obtained from de-identified surgical samples of glioma patients with surgical resections at Northwestern Memorial Hospital through the Northwestern Nervous System Tumor Bank (NSTB). All patients provided informed written consent. In addition, normal brain tissues were obtained from the NeuroBioBank at NIH (https://neurobiobank.nih.gov/).

Plasmids

LentiCRISPRv2GFP was a gift from Dr. David Feldser (Addgene plasmid # 82416) (8). Tet-pLKO-puro was a gift from Dmitri Wiederschain (Addgene plasmid # 21915) (9). Tet-pLKO-puro-Scrambled was a gift from Charles Rudin (Addgene plasmid # 47541) (10). pCMV-BE3 was a gift from David Liu (Addgene plasmid # 73021) (11). pLKO5.sgRNA.EFS.GFP was a gift from Benjamin Ebert (Addgene plasmid # 57822) (12). pSpCas9(BB)-2A-Puro (PX459) V2.0 was a gift from Feng Zhang (Addgene plasmid # 62988) (13). pLDPuro-hsnSR100N was a gift from Benjamin Blencowe (Addgene plasmid # 35172) (14). The coding sequence of CERS5 and MPZL1 isoforms, RBFOX1, and CELF4 were amplified by RT-PCR and ligated into a CD530-IRES-GFP vector (System Bioscience) with a fused Flag tag at C-terminal. pLV-EF1a-EGFRvIII-IRES-Hyg vector was obtained as recently reported (6).

Differentiation of hiPSCs to Neural Progenitor Cells (NPCs)

Generation of small molecule neural progenitor cells (smNPCs) from iPSCs was adapted from previous studies (6, 15). In detail, human iPSCs at 70–80% confluency were dissociated and resuspended at 1×10^6 cells/mL in N2B27 medium (50% DMEM/F12 and 50% Neurobasal medium with GlutaMAX, 1 × N-2 supplement, 1 × B-27 supplement, 1 × antibiotic-antimycotic, and 150 µM ascorbic acid) supplemented with 100 nM LDN-193189 (DNSK International), 10 µM SB431542 (DNSK International), 3 µM CHIR99021, 1 µM SAG and 10 µM Y-26732. Three million cells were transferred into one well of an uncoated six-well tissue culture plate and incubated at 37°C, 5% CO_2 on a shaker at 90 rpm. Uniform small embryoid bodies (EB) formed within 24 hr and increased in size over the following days. After 48 hr, a full medium change was performed with N2B27 medium supplemented with LDN-193189, SB431542, CHIR99021, and SAG, and half medium were refreshed every day for the flowing three days. On day 6, a full

medium change with NPC maintenance medium (N2B27 medium supplemented with 3 μM CHIR99021 and 1 μM SAG) was performed. At this stage, neuroepithelial folds were clearly visible in all Ebs. On day 8, Ebs were triturated by pipetting and plated onto matrigel-coated 10 cm plates. From then, smNPC cells were cultured in NPC maintenance medium and passaged at a 1:6 to 1:10 ratio with accutase (Innovative Cell Technologies) every two to five days. Total RNA was extracted from the differentiated smNPCs and qRT-PCR was performed to detect the expression of iPSC markers, *NANOG* and *POU5F1* (*OCT4*), and NPC markers, *NES* and *PAX6*.

3D Organoid Model of iPSC Glioma Avatars

The 3D organoid culture of iPSC glioma avatars was adapted from previous studies (16, 17). Briefly, smNPCs was dissociated and suspended in 80% Matrigel at 1×10^7 cells/mL. 20 μl matrigel-cell suspension is added in pressed parafilm molds to form pearl-shaped structures. These structures polymerize at 37°C for 1 hr, after which they were unmolded and cultured in NPC maintenance medium without shaking. After 24 hr, the nascent organoids were placed on an orbital shaker in a 37°C incubator for the remainder of culturing time. After another two days, medium was replaced with N2B27 medium plus 10% FBS to induce spontaneous differentiation of NPCs. Half medium was refreshed every day for the remainder of culturing time. After another 14 days, the organoids were harvested and subjected to further experiments.

RNA Isolation, Reverse transcription (RT)-PCR, and Quantitative (q)RT-PCR

Total RNA was isolated using a Qiagen RNeasy Mini Kit and was reverse transcribed using the iScript cDNA Synthesis Kit (Bio-Rad) according to the manufacturer’s instructions. To detect AS variants by RT-PCR, primers were designed flanking the alternative spliced exons or introns.

PCR products were separated by 1-1.5 % agarose gel, and the DNA product was quantified using the Image J software (NIH, US, version 1.8.0). The ratio of each variant was normalized to the sum of the different isoforms. Quantitative RT-PCR was performed using the EvaGreen 2 x qPCR MasterMix (Bullseye) on an Applied Biosystems StepOne Plus Real-Time Thermal Cycling Block with two to three replicates per group. Relative gene expression was determined by normalizing the expression of each target gene to ACTB. Results were analyzed using the $2^{-(\Delta\Delta Ct)}$ method. Primers are listed in Supplemental Table 8.

Immunoblotting (IB) and Immunoprecipitation (IP) Assay

Cells were lysed in modified NP-40 cell lysis buffer (50 mmol/L Tris-HCl, 150 mmol/L NaCl, 2 mmol/L EDTA, 1% NP-40, pH 7.4) containing 1x protease inhibitor cocktail and 1x phosphatase inhibitor cocktail (Sigma-Aldrich and Roche). To extract protein from tumor and normal brain tissues, the flow-through from the RNeasy spin column during RNA isolation was preserved and the proteins were precipitated using cold acetone according to the manufacturer's instructions. Protein samples were separated by SDS-PAGE and then transferred onto PVDF membranes. After blocking with 5% non-fat milk in TBS-T for 1 hr, membranes were incubated with indicated antibodies at desired dilutions overnight at 4℃. Following washing with TBS-T, the blot was incubated with corresponding HRP conjugated secondary antibodies. Blots were developed with enhanced chemiluminescence (ECL, Amersham Bioscience) reaction according to the manufacturer's instructions. GAPDH, β-actin, or α/β tubulin was used loading controls.

In IP experiment, cells were lysed in RIPA buffer containing 1x protease inhibitor cocktail and 1x phosphatase inhibitor cocktail (Sigma-Aldrich and Roche) on ice for 15 min and cleared by

centrifugation at 16,000 × g at 4℃ for 15 min. Cell lysate was subjected to immunoprecipitation (IP) with the anti-Flag M2-agarose affinity gel (Sigma-Aldrich, A2220) according to manufacturer's instructions.

CRISPR-mediated Gene Knockout

The target sequences of gRNA were designed using the SYNTHGO CRISPR Design Tool for knockouts (https://design.synthego.com) and cloned into the lentiCRISPRv2GFP plasmid (target sequence listed in Supplemental Table 7). Sequence-verified clones were used to produce lentiviral particles, which were used to infect GSC cells for 4 to 6 hr in the incubator. After four to six days, GFP positive cells were sorted with FACSMelody 3-Laser Sorter (BD Biosciences). Heterogeneous cell population were used for downstream experiment after validation of gene knockout by sanger sequencing and IB.

Doxycycline-inducible shRNA-mediated Gene Knockdown

RBP-targeting shRNAs were designed from the shRNA libraries on Broad Institute Genetic Perturbation Platform (https://portals.broadinstitute.org/gpp/public/) and cloned into Tet-pLKO-puro plasmid (target sequence listed in Supplemental Table 7). Sequence-verified clones were used to produce lentiviral particles, which were used to infect GSC cells for 4-6 hr in the incubator. After two to three days, cells were selected in 1 µg/mL puromycin for 3 days and maintained in 0.5 µg/mL puromycin for the following experiments. To induce shRNA expression, doxycycline at 1 µg/mL working dose was added to the medium. After three to five days, cell population were used for downstream experiment after validation of gene knockdown by IB. To induce neuronal differentiation in *PTBP1*-KD cells, GSC1478 cells infected with Tet-pLKO-puro-*PTBP1*-shRNA

or scramble control were plated on matrigel-coated plate and cultured for 10 days in neuronal differentiation medium, which includes N2B27 medium, 1 μM retinoic acid, 1 μM SAG, 5 μM DAPT (DNSK International), and 4 μM SU5402 (DNSK International), plus 1 μg/mL doxycycline.

Immunofluorescent (IF) and immunochemistry (IHC) Assay

For IF analyses, frozen brain tissue sections with xenografts were dried at room temperature for 20 min, and then fixed in PBS with 4% paraformaldehyde for 15 min. After 3 times wash in PBS, sections were blocked with AquaBlock (East Coast Bio, North Berwick, ME) for one hour and then incubated with appropriate primary antibodies at 4℃ for overnight, followed by staining with Alexa 488 or 594 labeled secondary antibodies (1:200) and DAPI-containing mounting solution Vectashield (Vector Laboratories). The images were acquired using a Nikon inverted microscope Eclipse Ti-U equipped with a digital camera. For IHC analyses, frozen subcutaneous tumor sections were dried at room temperature for 20 min, and then fixed in cold acetone at -20 ℃ for 10 min. After two times wash in PBS, sections were incubated for 10 min at room temperature in methano/peroxidase (3% H_2O_2 in methanol). After another two times wash in PBS, sections were blocked with AquaBlock (East Coast Bio, North Berwick, ME) for one hr and then incubated with appropriate primary antibodies at 4℃ for overnight, followed by staining with HRP-conjugated secondary antibodies and positive staining was developed using the Dako Liquid DAB+ Substrate Chromogen System (Dako, #K3467). The images were acquired using an Olympus BX53 microscope. Antibodies are listed in Supplemental Table 10.

Flow Cytometric Detection of EGFR/EGFRvIII Expression

Single cell suspensions from edited NPCs were washed in cold PBS and then incubated with an anti-EGFR antibody (1:50 dilution, Invitrogen, #MA5-13269), which recognizes both EGFR-WT and vIII isoform, at 4°C for 3 hr, followed by incubation with Alexa Fluor 488 anti-mouse IgG for another 1 hr. Cells were measured with the flow cytometer LSRII (BD Biosciences) and analyzed with FlowJo software (Tree Star Inc.). As a control, a nonspecific mouse IgG isotype control (Santa Cruz, sc-2025) was used to determine nonspecific binding.

RNA-sequencing in iPSC Models

Libraries for Illumina sequencing were prepared using NEBNext Poly(A) mRNA Magnetic Isolation Module, Ultra II RNA Library Prep Kit, and Multiplex Oligos (NEB, Cat #E7490, E7775, E6609) according to the manufacturer’s instruction. Sequencing was performed on the Illumina NovaSeq platform using 150 bp paired-end run with 70-100 million paired reads per sample at the Northwestern University NUSeq core facility. AS and gene expression analyses were performed as previously described (1). For the iPSC avatar xenografted samples, the Disambiguate (18) tool was used to separate sequence reads originating from human iPSC avatar cells and mouse stromal component after alignment. The reads assigned to the human were used for downstream gene expression and AS analysis.

In vitro Cell Proliferation Assays

Dissociated cells were seeded in 96-well plates at density of 1,000 or 2000 cells per well, 3 to 4 replicates per group. Cell viability was measured using CellTiter-Glo Luminescent Cell Viability Assay Kit (Promega) at day 0, 2, 4, and 6 according to the manufacturer's instructions.

Luminescence was measured using SpectraMax M3 Multi-Mode Microplate Reader (Molecular Devices) and normalized to their levels at day zero.

In vitro Limiting Dilution Assays

GSCs or edited iPSC-derived NPCs were dissociated into single-cell suspensions and seeded into 96-well plates at density of 1, 2, 4, 8, 16, or 32 cells per well. Cells were incubated at 37℃ for 1 to 2 weeks. At the time of quantification, each well was examined for formation of tumor spheres. Stem cell frequency was calculated using extreme limiting dilution analysis online tool (http://bioinf.wehi.edu.au/software/elda/) (19).

Survival Analysis in Glioma Patients

The overall survival data of patients with glioma were downloaded from GDC Data Portal (https://portal.gdc.cancer.gov) and CGGA website (http://cgga.org.cn). Survival of patients was estimated and analyzed using the Kaplan-Meier (KM) analyses. Log-rank tests were used to compare KM curves between groups. Multiple-predictor models were fit using the Cox Proportional Hazards model. Hazard ratios (HR) and 95% CIs were reported. The analyses were conducted in the R Statistical Environment (v 4.0.1) using the “survival” and “survminer” packages.

Genomic and Transcriptomic Analysis from Glioma Datasets

The gene mutation and copy number alteration information of TCGA samples were acquired from cBioPortal online tools (www.cbioportal.org). The *TERT* promoter, *IDH1,* and 1p/19q status for TCGA samples were downloaded from GlioVis online tools, http://gliovis.bioinfo.cnio.es. The

normalized FPKM values of TCGA samples were downloaded from GDC Data Portal, https://portal.gdc.cancer.gov. The normalized RSEM values from Chinese Glioma Genome Atlas (CGGA) RNA-seq datasets were downloaded from their website, http://cgga.org.cn. Gene expression analysis were performed in NU samples using HTSeq (20) and DESeq2 (21). A list of neural lineage marker genes was created based on website https://www.abcam.com/neuroscience/neural-markers-guide. A list of 276 human splicing-regulating RBP genes was created from the overlay of Gene Ontology annotation GO:0000398, "mRNA splicing", and GO:0003723, "RNA binding" on the website, http://amigo.geneontology.org.

AS and Gene Expression Analysis in other Bulk RNA-seq Datasets

Publicly available RNA-seq data from GSE90553 (22), GSE76476 (23), and GSE130501 (1) were downloaded from European Nucleotide Archive (www.ebi.ac.uk/ena/). RNA-seq data from ENCSR415DJT (24) were downloaded from the ENCODE Portal (www.encodeproject.org). RNA-seq data of normal adult and fetal brains from EGAD00001005131 (25) were downloaded from European Genome-phenome Archive. AS and gene expression analyses were performed as previously described (1).

Splice Site Strength Estimation

MaxEntScan online tool (26) was utilized to determine the splice site strength for specific exons. For 3'SS, 23 bases with 20 bases in intron and 3 bases in exon were used to calculate the splice site strength; while for 5'SS, 9 bases with 3 bases in exon and 6 bases in intron were used to calculate the splice site strength. The maximum entropy model (MAXENT), first-order Markov

model (MM), and weight matrix model (WMM) were utilized for scoring. P-value was calculated through unpaired Wilcoxon test between two groups of exons.

GC Content Estimation around Splice Site

A region from -64 bp to -3 bp and 1-64 bp surrounding exonic boundaries was used to determine the GC content around the splice site. GC content was examined in an 8 bp sliding window at both the 3'SS and 5'SS of exons through R package Biostrings (v2.64.1). Distance refers to how far the examining site was away from the examining starting point. For 3'SS, distance increases as closer to the exonic boundary; while for 5'SS, distance increases as further away from the exonic boundary. P-value was calculated through two-way ANOVA.

Motif Discovery Analysis at Splicing Sites

The MEME motif discovery (http://meme-suite.org/tools/meme) was performed in each of the three regions around the cassette exons of 145 SEs plus 19 pairs of MXEs among the 200-AS event list: the last 300 nt of the upstream intron, the whole exon region, and the first 300 nt of the downstream intron. The cassette exons of all annotated SE events were defined as background. Identification of significant motifs was achieved by running the MEME analysis with the following parameters: Motif discovery mode–Differential Enrichment mode; Site distribution–any number of repetitions; Motif width: 6-10 nt; search the given strand only; E-value cutoff: 0.05; other parameters set as default. The enriched motifs were used as input to search potential interacted RBPs on the ATtRACT database (https://attract.cnic.es/searchmotif) based on the following parameters: organism - home sapiens; Experiment - RNA immunoprecipitation or RNA

pulldown; Qscore (Quality score that detects the binding affinity between RBPs and binding sites, range 0-1) = 1.

Analysis of Lipidome and Proteome Data from CPTAC-GBM Dataset

The analyzed lipid abundance data from CPTAC-GBM dataset were downloaded from the supplementary data of previous publication (27). Two-tailed unpaired student's t-test was used to compare the ceramide abundance between normal brain and GBM samples. The raw proteome data from the CPTAC GBM Discovery Study (PDC000204) were downloaded from PDC portal, https://pdc.cancer.gov/pdc. The data processing of the raw data involved using the Proteome Discoverer (v3.0.0.757; ThermoFisher Scientific) data analysis pipeline. The Sequest HT search engine was used for configuration, and a 1% FDR cutoff was applied to filter the data, which was estimated by Target Decoy PSM Validator. The precursor mass tolerance was set at 10 ppm, and the fragment mass tolerance was 0.6 Da. The maximum mass cleavages were set to 3, and the minimum and maximum peptide lengths were 6 and 144, respectively. Dynamic modification of oxidation (+15.995 Da on M) was used, while static modifications of TMT label (+229.163 Da on N-terminus and K) and Carbamidomethyl (+57.021 Da on C) were applied. The database contained CPTAC isoform 1 (iso1) and isoform 2 (iso2) sequences, and a co-isolation threshold of 50% was set to minimize the influence of co-isolated peptides on quantification. To determine the abundance of each peptide uniquely mapped to CERS5, the ratio of sample abundance to reference abundance was calculated. A TMT 126 reagent labeled the pooled reference sample, enabling the comparison of relative protein abundances across different TMT-11 plexus (27). The mean abundance of peptides that were specific to iso 1, iso 2, or the common

region of CERS5, were calculated respectively. We used these values to compare the levels of CERS5 isoforms between normal brain and GBM tissues.

Ceramide extraction and liquid-chromatography mass spectrometry analysis

GSC1478 and GSC83 cells with manipulated CERS5 expression were seeded at a density of 1×10^5 cells/mL in GSC medium. After 48 hr, the cells were harvested for lipidomic profiling. GSC1478 cells infected with Tet-pLKO-puro-*PTBP1*-shRNA or scramble control were plated on matrigel-coated plate and cultured for 10 days in neuronal differentiation medium with medium changes every other day prior to their ceramide profiling. For lipid extraction, an equal number of cells were centrifuged at 600 g for 5 min at room temperature and the medium was aspirated off. Then cells were washed with PBS and the metabolism was quenched by adding 0.2 mL of dry ice-cold methanol. The extraction procedure was adapted from a previous publication by Matyash et al (28). Samples were stored at -80°C until the analysis. On the day of the analysis, samples were thawed on ice and vortex. Then 1 mL of ice-cold methyl tert butyl ether was added, sonicated in ice cold water bath for 3 min and shaken for 5 min at 2000 rpm and 4°C. To induce the phase separation, 0.2 mL of LC-MS grade water was added and shaken for 3 min at 2000 rpm and 4°C. Samples were centrifuged at 20,000g and 4°C for 20 min. 0.9 mL of upper phases were collected and dried down using the Genevac EZ-2.4 elite evaporator. The dried-down samples were re-suspended in 50 µL of 3/2/1 Isopropanol/ acetonitrile/water before LC-MS analysis.

Ceramide separation and detection were performed using Thermo Scientific Vanquish Horizon UHPLC system coupled (Accucore C30 column,2.1x150 mm, 2.6 µM; part # 27826152130) to

Orbitrap IQ-X Tribrid mass spectrometer with an H-ESI probe operating in either positive or negative mode. The mobile phase A (MPA) was 60/40 acetonitrile/water containing 10 mM ammonium formate+0.1% formic acid and MPB was 90/10 isopropanol/acetonitrile+10 mM ammonium formate+0.1% formic acid. The column temperature, injection volume, and flow rate were 45°C, 5 µL or 10 µL, and 0.260 mL/min, respectively. The chromatographic gradient was 0 min: 30% B, 2 min:43% B, 2.1 min 55% B,12.00 min: 65% B, 18.00 min: 85% B, 20.00 min:100% B, 25 min: 100% B, 25.1 min: 30% B and 30.00 min: 30% B. MS parameters were as follows: spray voltage: 3500 V for positive ionization and 2500 V for negative ionization modes, sheath gas: 40, auxiliary gas: 10, sweep gas: 1, ion transfer tube temperature: 300°C, vaporizer temperature: 350°C, MS1 resolution: 120K, acquisition range: 200-1700, RF lens:45, automatic gain control (AGC) target: 100%, and a maximum injection time of 246 milliseconds (ms). For the positive mode, parallel MS2 was performed in orbitrap at 15K resolution. The precursor ions were isolated by quadrupole at 1.4 m/z width and fragmented by higher-energy collision dissociation (HCD) at 25,30 stepped normalized collision energy, normalized AGC target:100%, and max injection time of 22 ms. For the negative ionization mode, ceramides species targeted MS2 were performed using similar parameters as the positive mode. The ceramide species identification was done by confirming the presence of fragment ions at m/z of 264.2686,252.2686,282.2791 for d18:1, 262.2529,250.2529,280.2635 for d:18:2, 266.2842,254.2842,284.2948 for d18:0 in the positive ionization mode. LipidSearch (v5.0, ThermoFisher Scientific) was used to confirm the identification in the positive and negative ionization mode data. Data analysis was performed using Tracefinder (v5.1, ThermoFisher Scientific).

Synthesis and in vitro transfection of antisense oligonucleotides (ASOs)

ASOs were synthesized by Integrated DNA Technologies. All ASOs were modified with phosphorothioate backbone. An ASO targeting GFP was used as a control. The sequence of ASOs were list in Supplemental Table 7. GSC83, GSC1478, and GSC1485 cells were transfected with ASOs at 50-100 nmol/L by electroporation using a Neon Transfection System (Invitrogen, #MPK5000) according to the manufacturer's instructions. U87, NHNPs, NHAs, and edited iPSC-derived NPCs were transfected with ASOs at 50-100 nmol/L using Lipofectamine 2000 (Thermo Fisher Scientific) according to the manufacturer's instructions. The transfected cells were then used for experiments conducted at 36 to 48 hr after transfection.

REFERENCES

1. Song X, Wan X, Huang T, Zeng C, Sastry N, Wu B, et al. SRSF3-Regulated RNA Alternative Splicing Promotes Glioblastoma Tumorigenicity by Affecting Multiple Cellular Processes. *Cancer Res.* 2019;79(20):5288-301.

2. Mao P, Joshi K, Li J, Kim SH, Li P, Santana-Santos L, et al. Mesenchymal glioma stem cells are maintained by activated glycolytic metabolism involving aldehyde dehydrogenase 1A3. *Proc Natl Acad Sci U S A.* 2013;110(21):8644-9.

3. Srikanth M, Das S, Berns EJ, Kim J, Stupp SI, and Kessler JA. Nanofiber-mediated inhibition of focal adhesion kinase sensitizes glioma stemlike cells to epidermal growth factor receptor inhibition. *Neuro Oncol.* 2013;15(3):319-29.

4. Jones LE, Hilz S, Grimmer MR, Mazor T, Najac C, Mukherjee J, et al. Patient-derived cells from recurrent tumors that model the evolution of IDH-mutant glioma. *Neurooncol Adv.* 2020;2(1):vdaa088.

5. Yu D, Liu Y, Zhou Y, Ruiz-Rodado V, Larion M, Xu G, and Yang C. Triptolide suppresses IDH1-mutated malignancy via Nrf2-driven glutathione metabolism. *Proc Natl Acad Sci U S A.* 2020;117(18):9964-72.

6. Koga T, Chaim IA, Benitez JA, Markmiller S, Parisian AD, Hevner RF, et al. Longitudinal assessment of tumor development using cancer avatars derived from genetically engineered pluripotent stem cells. *Nat Commun.* 2020;11(1):550.

7. Miki S, Koga T, McKinney AM, Parisian AD, Tadokoro T, Vadla R, et al. TERT promoter C228T mutation in neural progenitors confers growth advantage following telomere shortening in vivo. *Neuro Oncol.* 2022.

8. Walter DM, Venancio OS, Buza EL, Tobias JW, Deshpande C, Gudiel AA, et al. Systematic In Vivo Inactivation of Chromatin-Regulating Enzymes Identifies Setd2 as a Potent Tumor Suppressor in Lung Adenocarcinoma. *Cancer Res.* 2017;77(7):1719-29.

9. Wiederschain D, Wee S, Chen L, Loo A, Yang G, Huang A, et al. Single-vector inducible lentiviral RNAi system for oncology target validation. *Cell Cycle.* 2009;8(3):498-504.

10. Rudin CM, Durinck S, Stawiski EW, Poirier JT, Modrusan Z, Shames DS, et al. Comprehensive genomic analysis identifies SOX2 as a frequently amplified gene in small-cell lung cancer. *Nat Genet.* 2012;44(10):1111-6.

11. Komor AC, Kim YB, Packer MS, Zuris JA, and Liu DR. Programmable editing of a target base in genomic DNA without double-stranded DNA cleavage. *Nature.* 2016;533(7603):420-4.

12. Heckl D, Kowalczyk MS, Yudovich D, Belizaire R, Puram RV, McConkey ME, et al. Generation of mouse models of myeloid malignancy with combinatorial genetic lesions using CRISPR-Cas9 genome editing. *Nat Biotechnol.* 2014;32(9):941-6.

13. Ran FA, Hsu PD, Wright J, Agarwala V, Scott DA, and Zhang F. Genome engineering using the CRISPR-Cas9 system. *Nat Protoc.* 2013;8(11):2281-308.

14. Raj B, O'Hanlon D, Vessey JP, Pan Q, Ray D, Buckley NJ, et al. Cross-regulation between an alternative splicing activator and a transcription repressor controls neurogenesis. *Mol Cell.* 2011;43(5):843-50.

15. Reinhardt P, Glatza M, Hemmer K, Tsytsyura Y, Thiel CS, Hoing S, et al. Derivation and expansion using only small molecules of human neural progenitors for neurodegenerative disease modeling. *PLoS One.* 2013;8(3):e59252.

16. Hubert CG, Rivera M, Spangler LC, Wu Q, Mack SC, Prager BC, et al. A Three-Dimensional Organoid Culture System Derived from Human Glioblastomas Recapitulates the Hypoxic Gradients and Cancer Stem Cell Heterogeneity of Tumors Found In Vivo. *Cancer Res.* 2016;76(8):2465-77.

17. Sundar SJ, Shakya S, Barnett A, Wallace LC, Jeon H, Sloan A, et al. Three-dimensional organoid culture unveils resistance to clinical therapies in adult and pediatric glioblastoma. *Transl Oncol.* 2022;15(1):101251.

18. Ahdesmaki MJ, Gray SR, Johnson JH, and Lai Z. Disambiguate: An open-source application for disambiguating two species in next generation sequencing data from grafted samples. *F1000Res.* 2016;5:2741.

19. Hu Y, and Smyth GK. ELDA: extreme limiting dilution analysis for comparing depleted and enriched populations in stem cell and other assays. *J Immunol Methods.* 2009;347(1-2):70-8.

20. Putri GH, Anders S, Pyl PT, Pimanda JE, and Zanini F. Analysing high-throughput sequencing data in Python with HTSeq 2.0. *Bioinformatics.* 2022;38(10):2943-5.

21. Anders S, and Huber W. Differential expression analysis for sequence count data. *Genome Biol.* 2010;11(10):R106.

22. Ziller MJ, Ortega JA, Quinlan KA, Santos DP, Gu H, Martin EJ, et al. Dissecting the Functional Consequences of De Novo DNA Methylation Dynamics in Human Motor Neuron Differentiation and Physiology. *Cell Stem Cell.* 2018;22(4):559-74 e9.

23. Damianov A, Ying Y, Lin CH, Lee JA, Tran D, Vashisht AA, et al. Rbfox Proteins Regulate Splicing as Part of a Large Multiprotein Complex LASR. *Cell.* 2016;165(3):606-19.

24. Consortium EP. An integrated encyclopedia of DNA elements in the human genome. *Nature.* 2012;489(7414):57-74.

25. Jessa S, Blanchet-Cohen A, Krug B, Vladoiu M, Coutelier M, Faury D, et al. Stalled developmental programs at the root of pediatric brain tumors. *Nat Genet.* 2019;51(12):1702-13.

26. Yeo G, and Burge CB. Maximum entropy modeling of short sequence motifs with applications to RNA splicing signals. *J Comput Biol.* 2004;11(2-3):377-94.

27. Wang LB, Karpova A, Gritsenko MA, Kyle JE, Cao S, Li Y, et al. Proteogenomic and metabolomic characterization of human glioblastoma. *Cancer Cell.* 2021;39(4):509-28 e20.

28. Matyash V, Liebisch G, Kurzchalia TV, Shevchenko A, and Schwudke D. Lipid extraction by methyl-tert-butyl ether for high-throughput lipidomics. *J Lipid Res.* 2008;49(5):1137-46.
