# Supplementary material for: RNA splicing analysis deciphers developmental hierarchies and reveals therapeutic targets in adult glioma
Source: J Clin Invest. 2024 Apr 25;134(11):e173789. doi: 10.1172/JCI173789 (PMC11142752; doi:10.1172/JCI173789)

Full unedited gel for Figure 2I

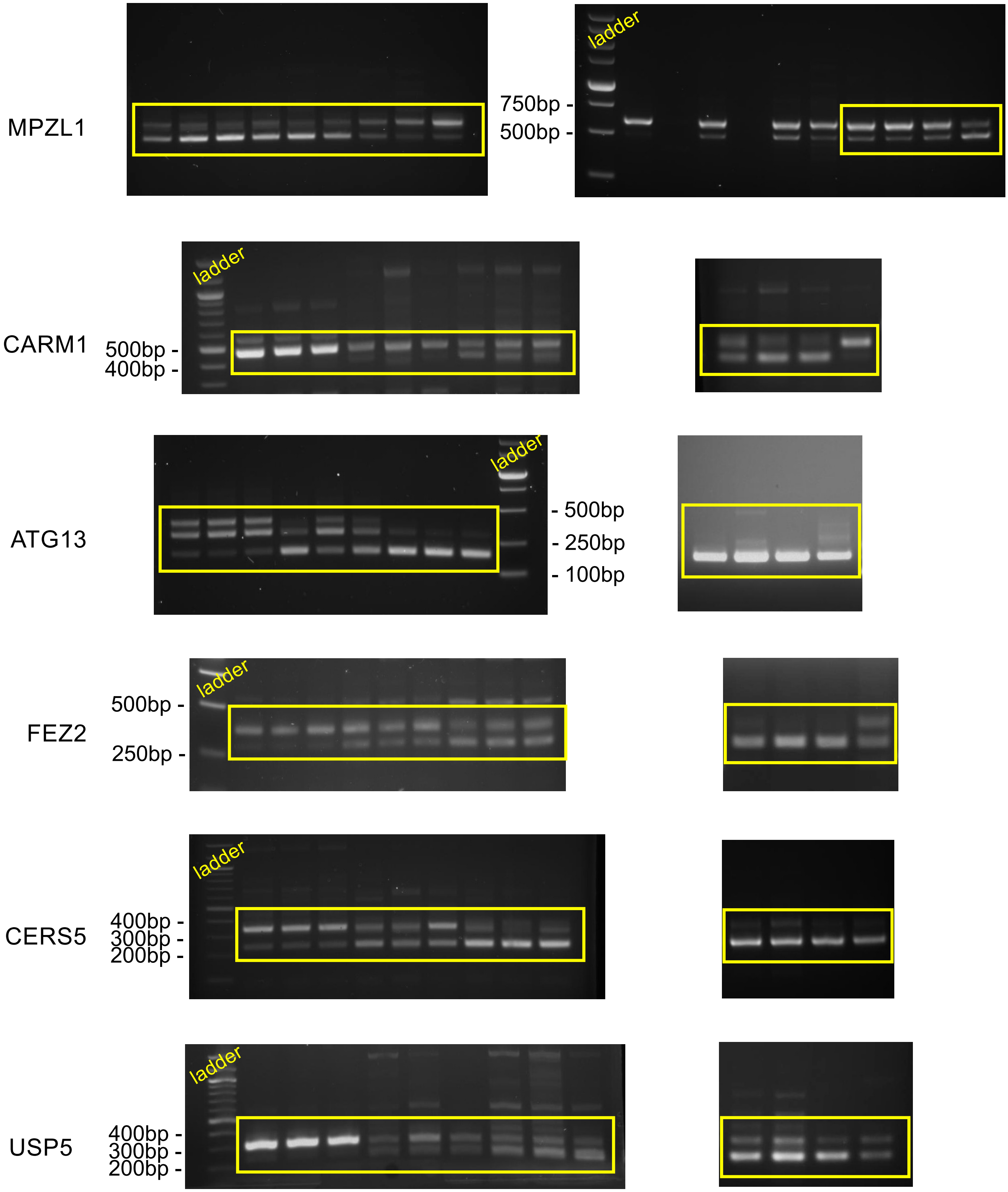

Full unedited gel for Figure 2I

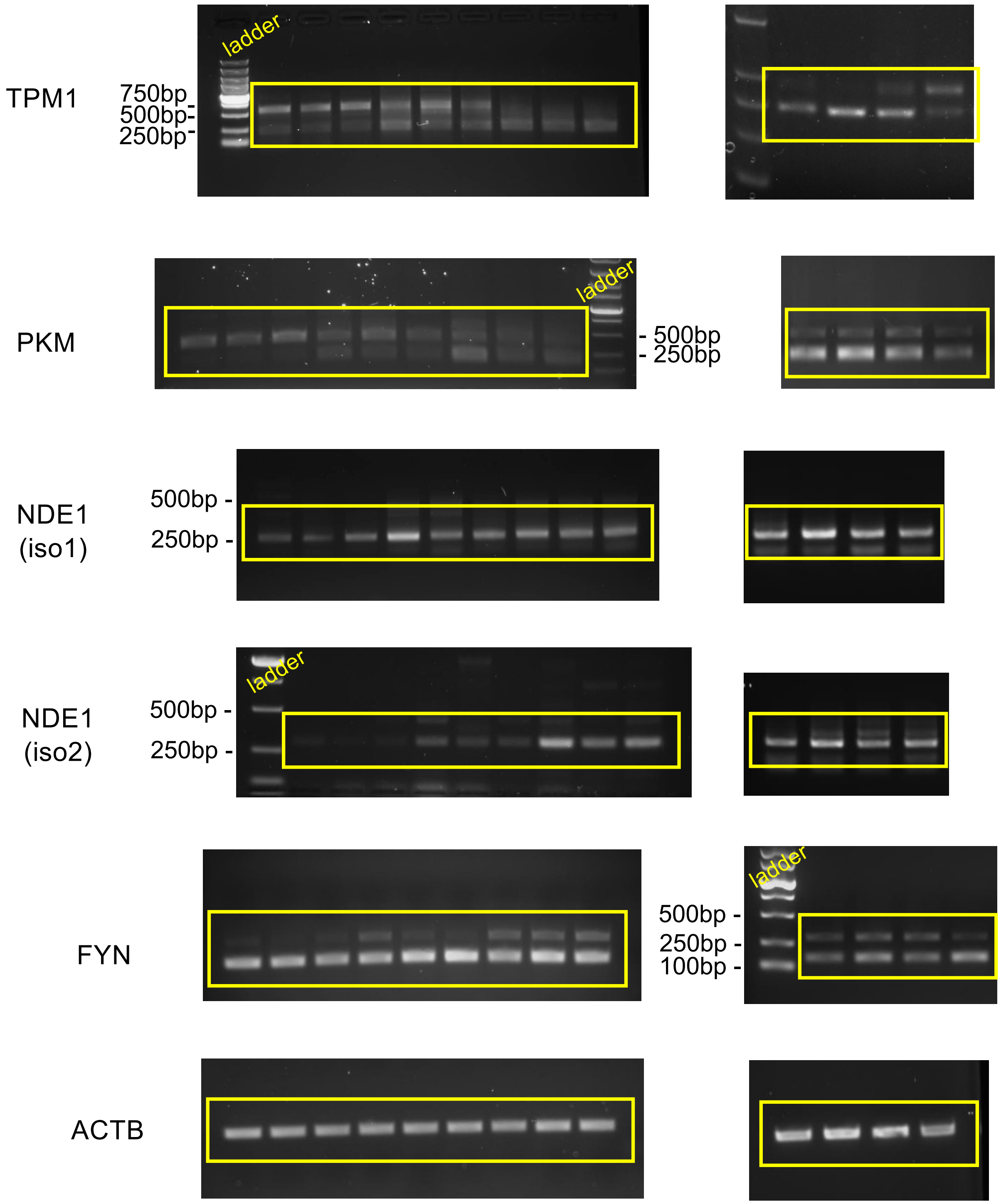

Full unedited gel for Figure 4C

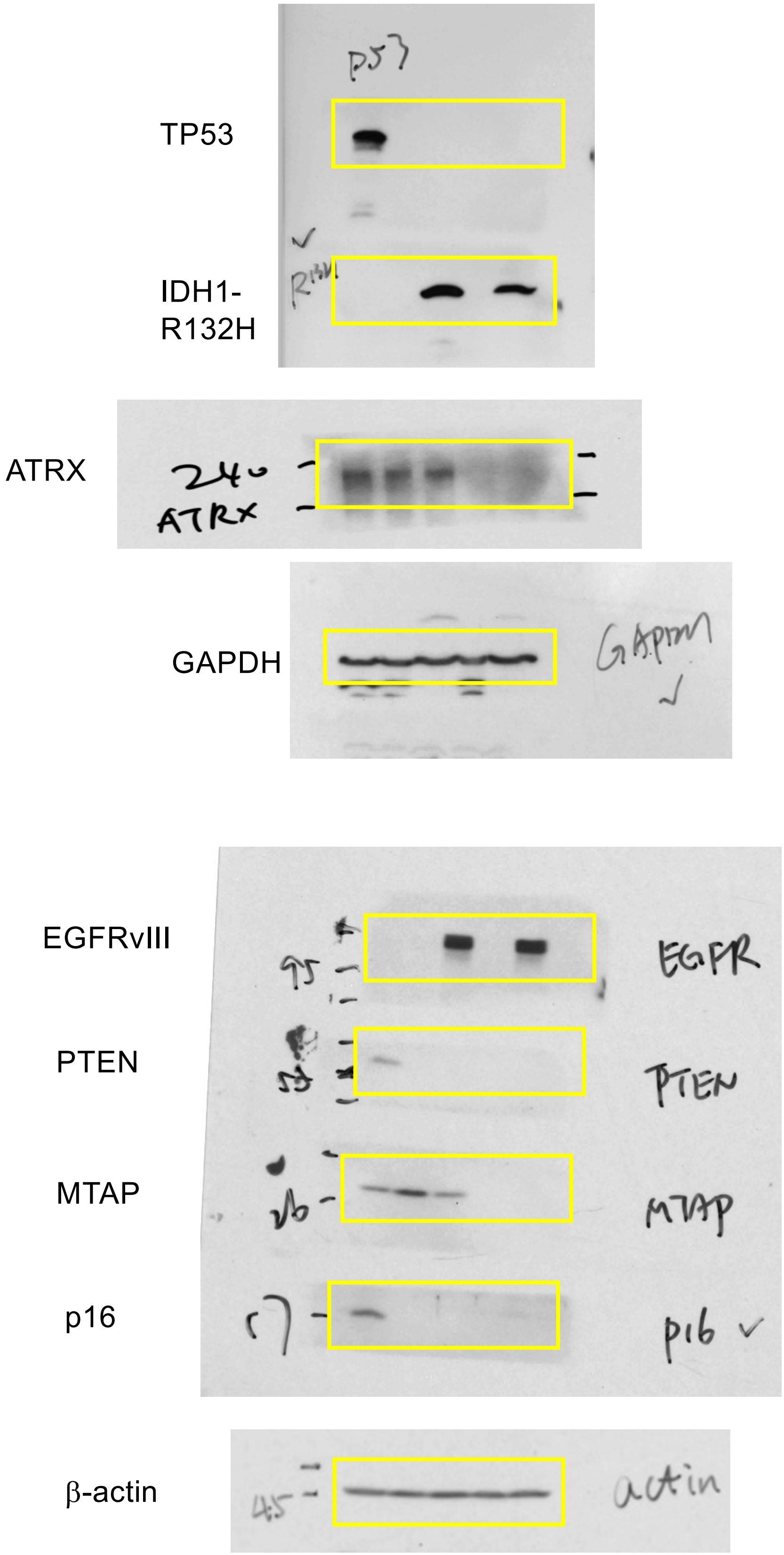

Full unedited gel for Figure 5F

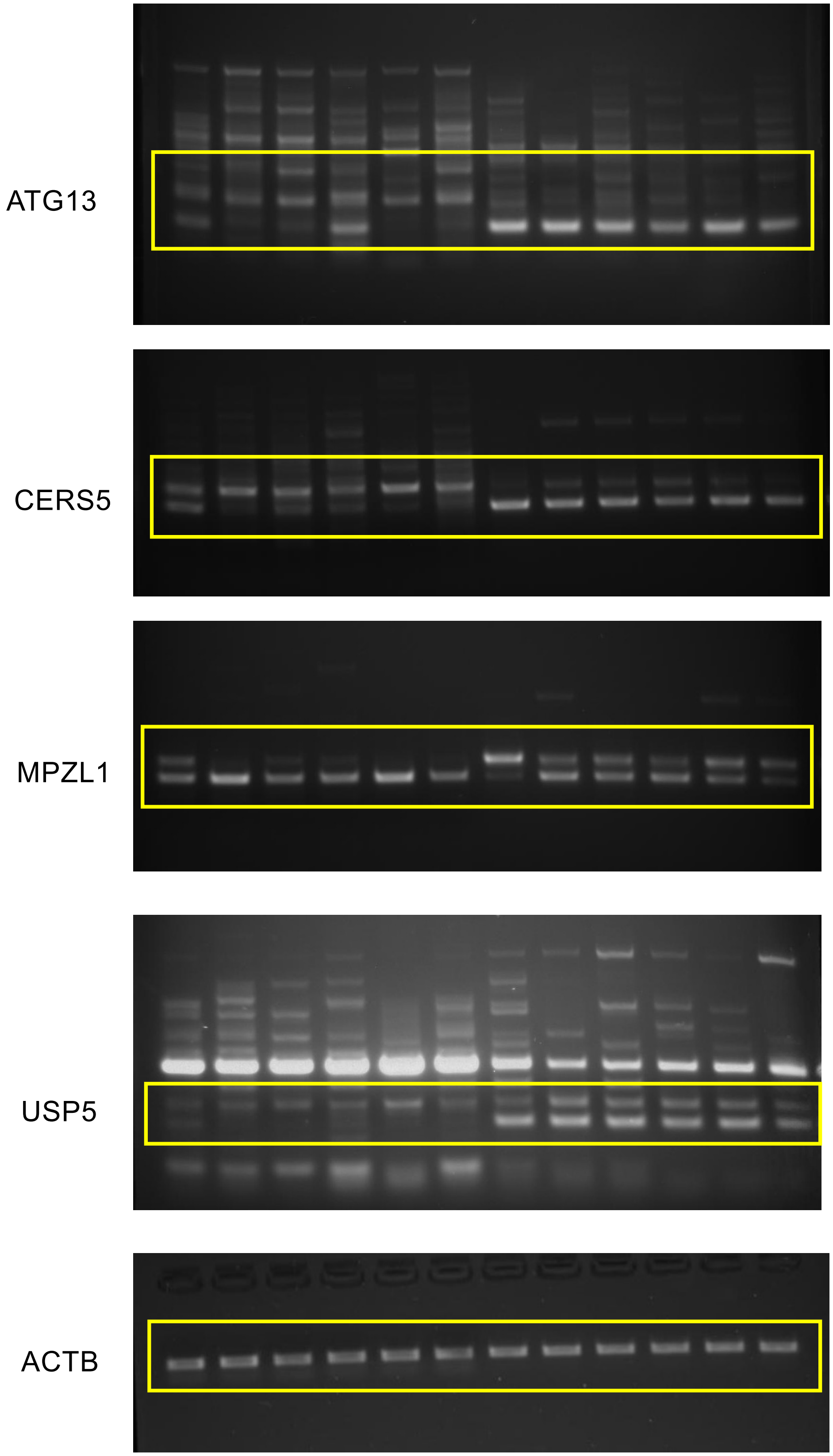

Full unedited gel for Figure 6G

p-Ser/Thr

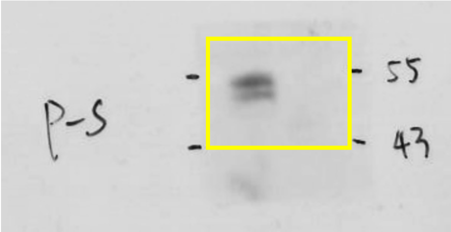

FLAG

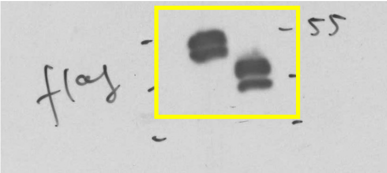

Full unedited gel for Figure 7B

TPM1

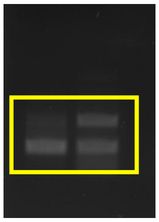

MPZL1

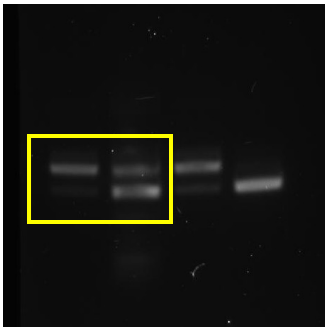

CSNK1D

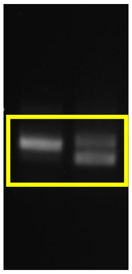

PTPRF

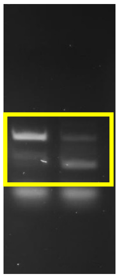

MARK3

500bp -
250bp -

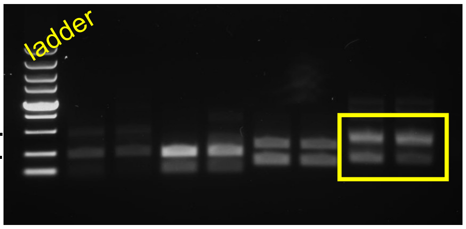

TJP2

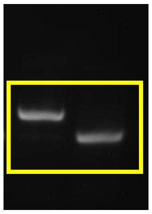

Full unedited gel for Figure 7C

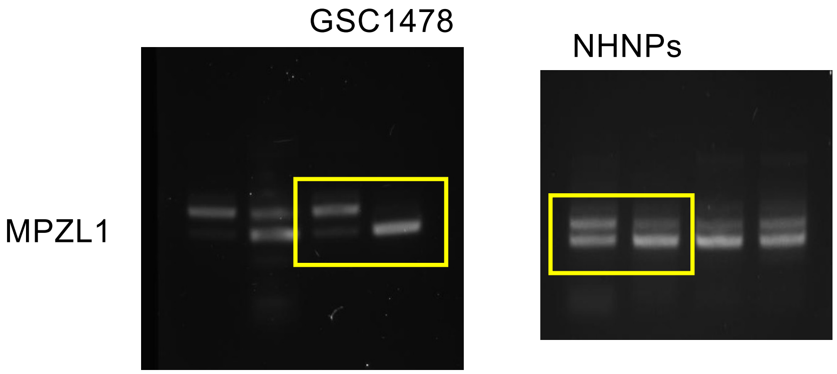

Full unedited gel for Figure 7G

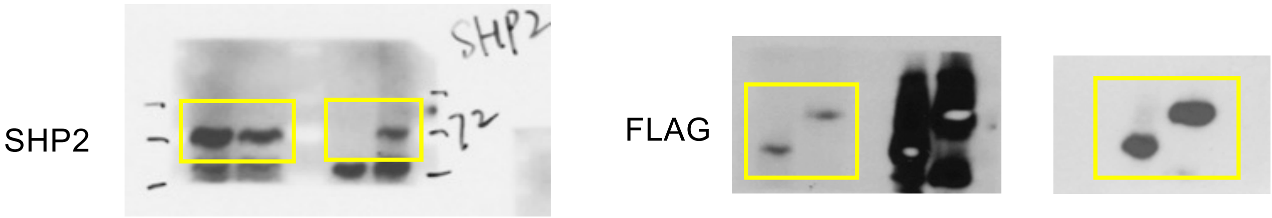

Full unedited gel for Figure 7H

p-AKT

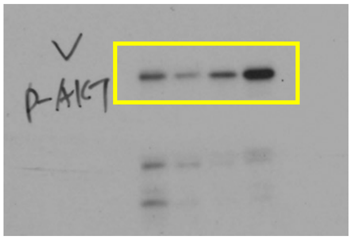

p-ERK

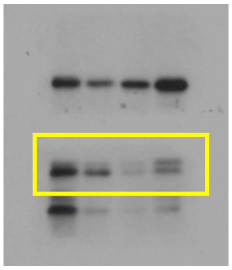

MPZL1

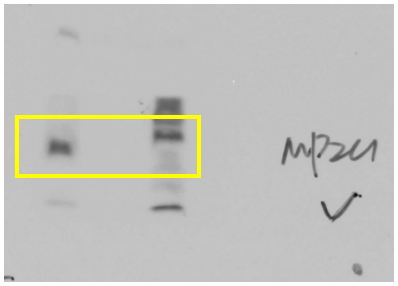

GAPDH

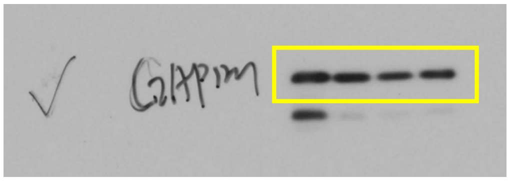

FLAG

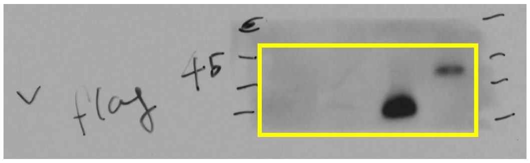

Full unedited gel for Figure 8C

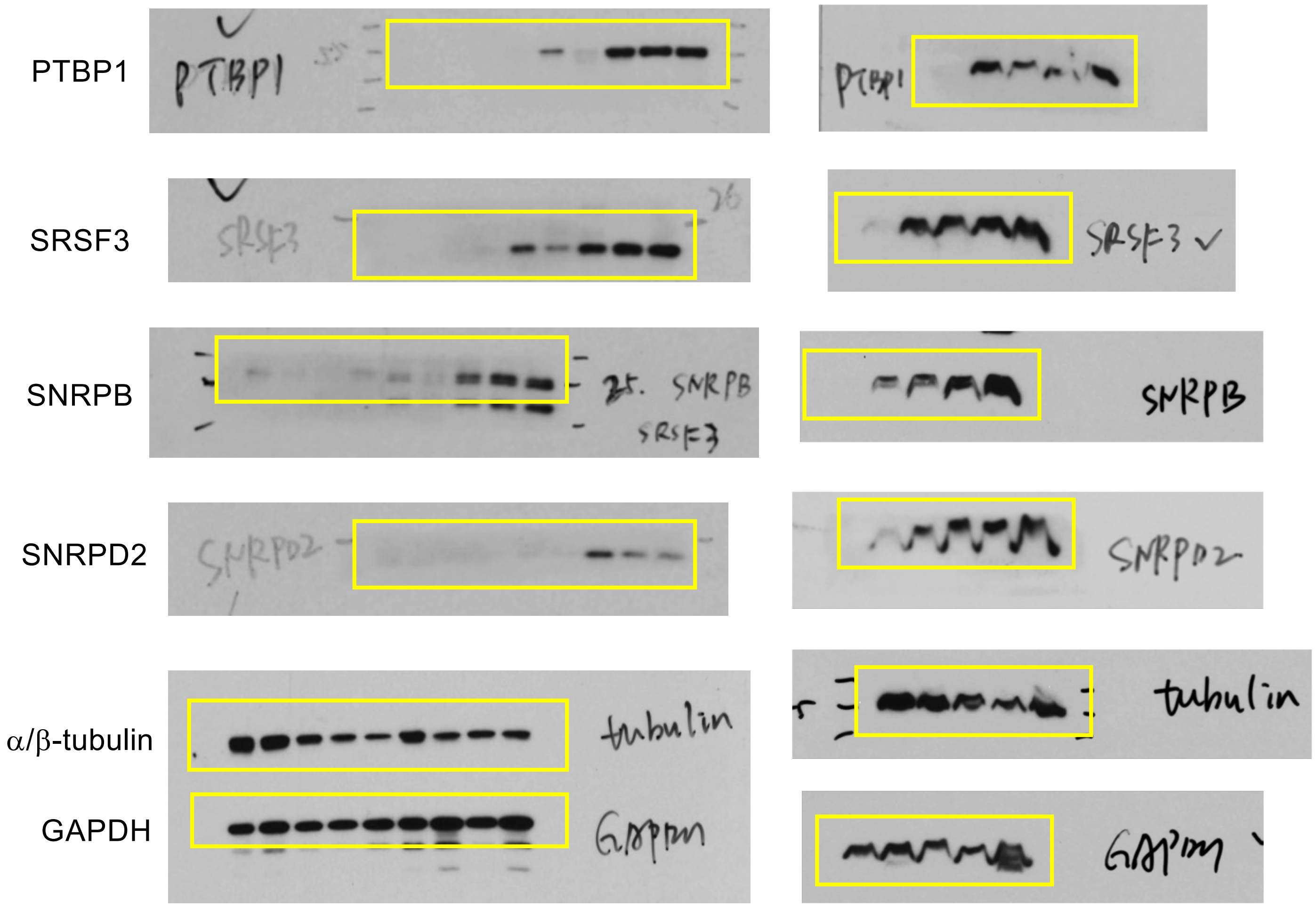

Full unedited gel for Figure 8E

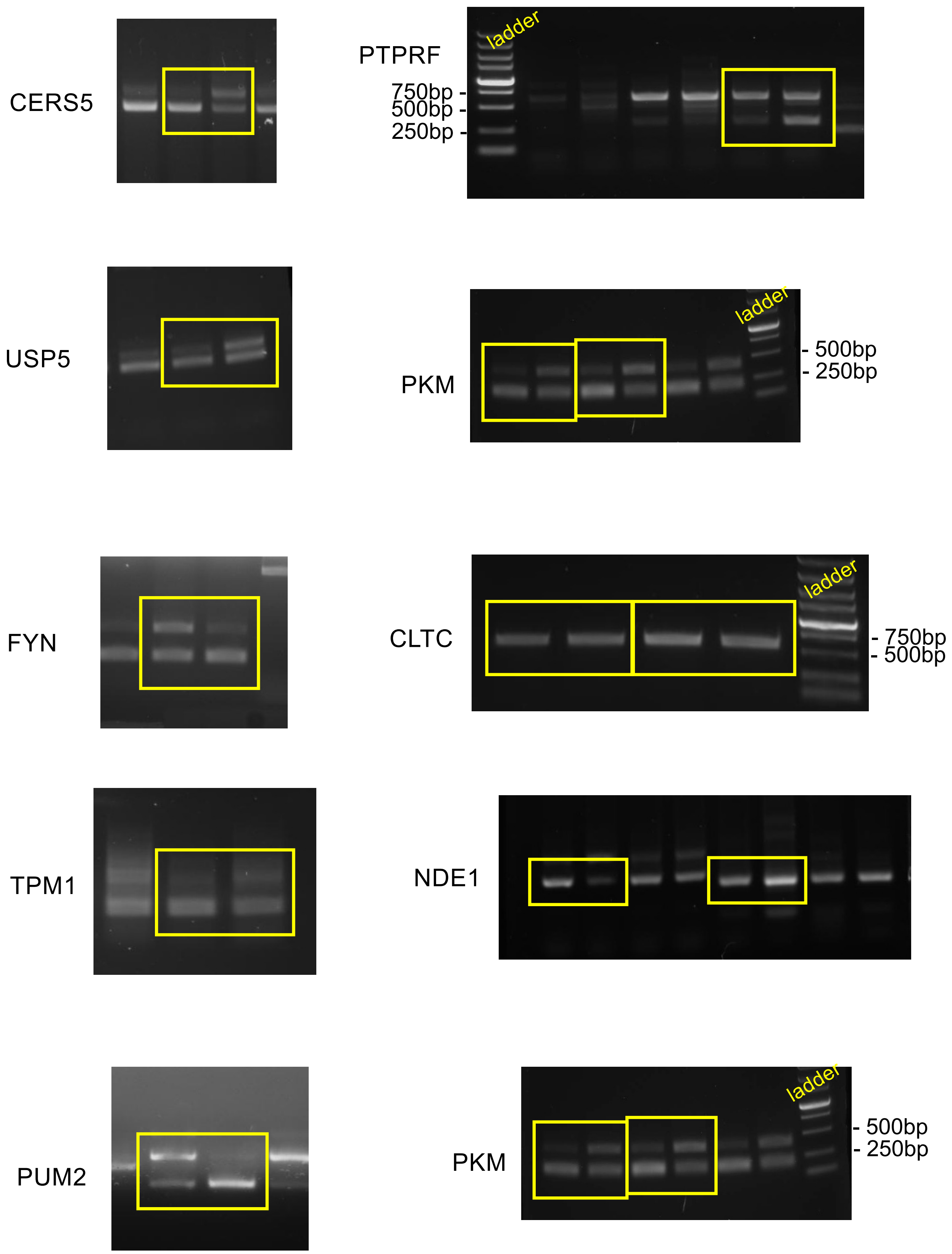

Full unedited gel for Figure 9A

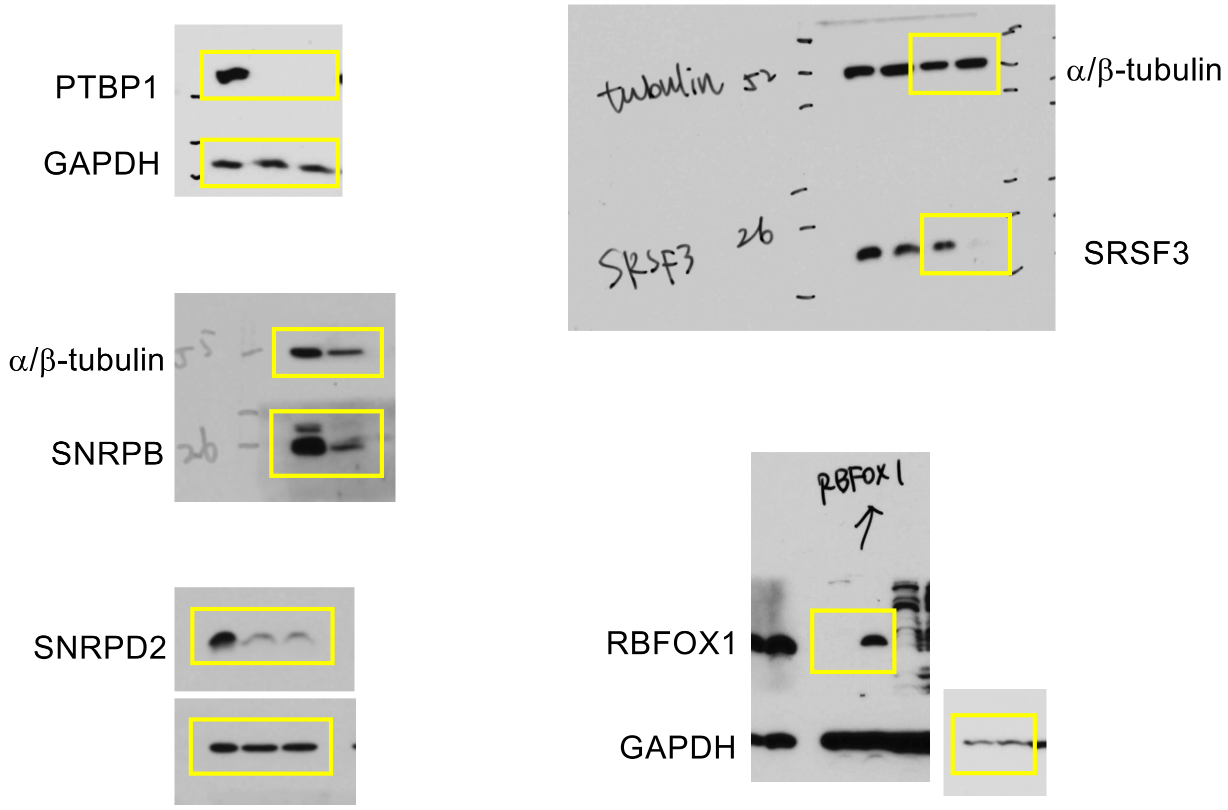

Full unedited gel for Figure 9C, right panel

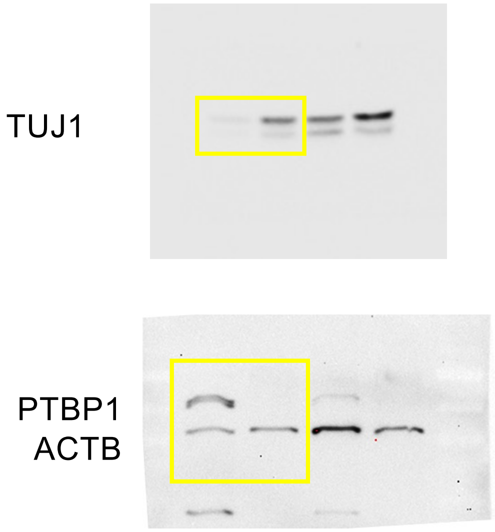

Full unedited gel for Figure 9D

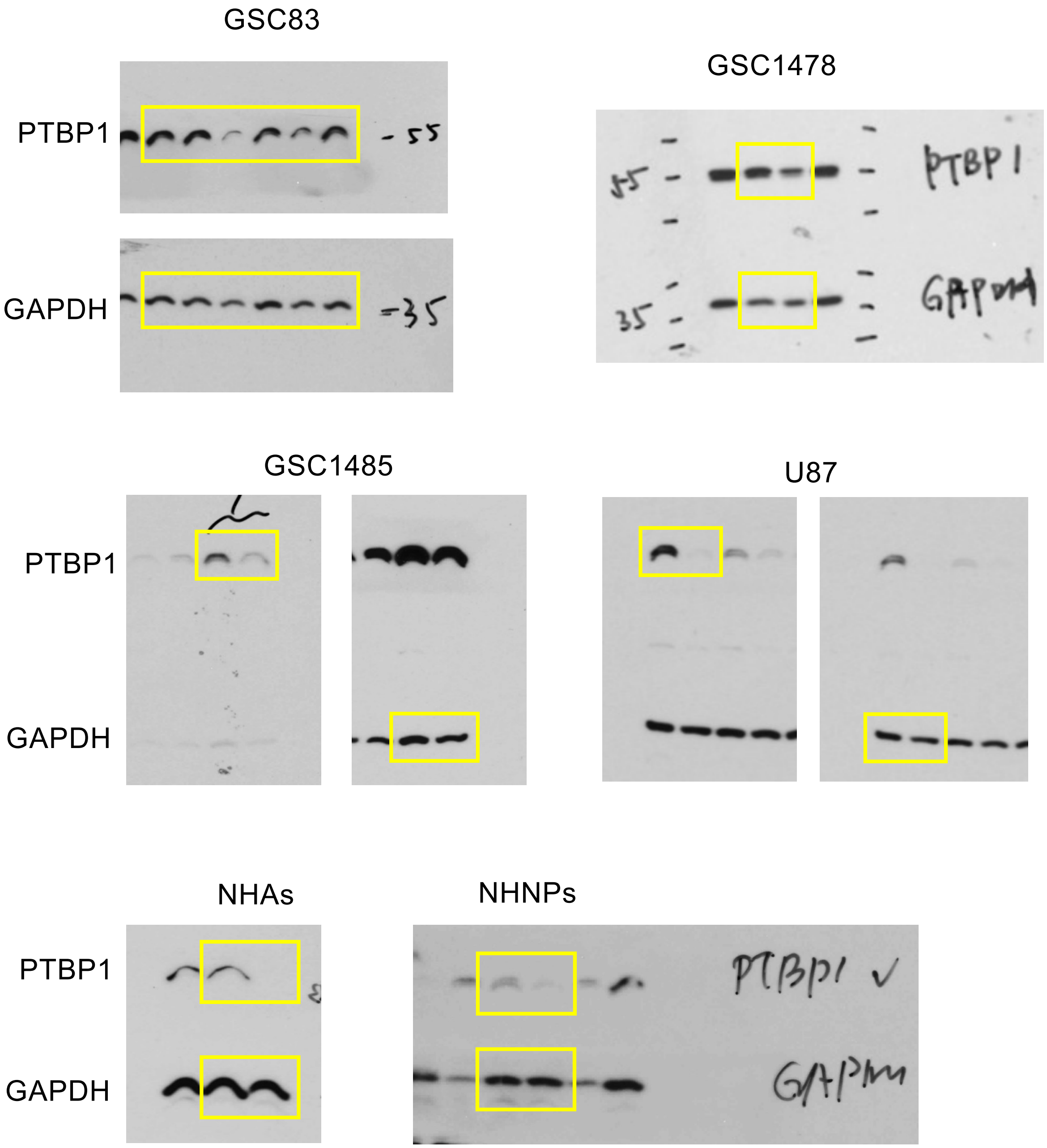

Full unedited gel for Figure 9E

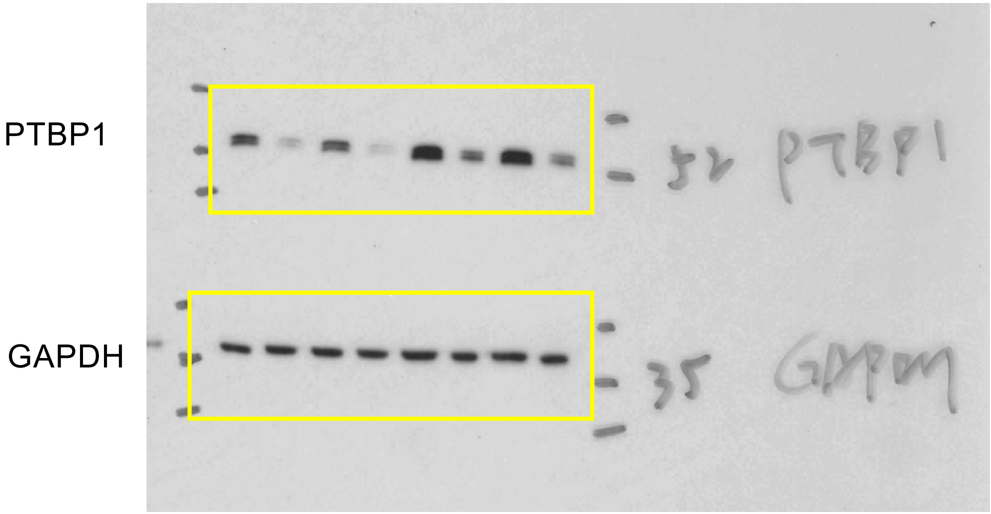

Full unedited gel for Supplemental Figure 4D

PTEN

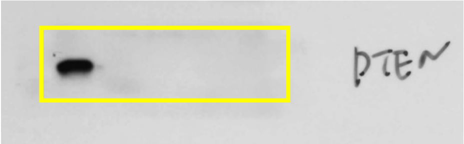

p16

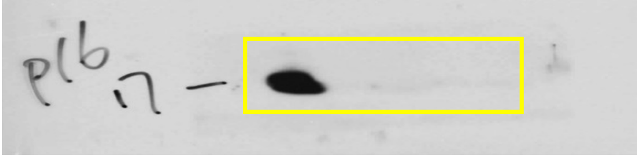

EGFRvIII

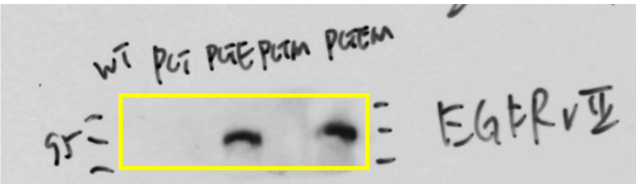

MTAP

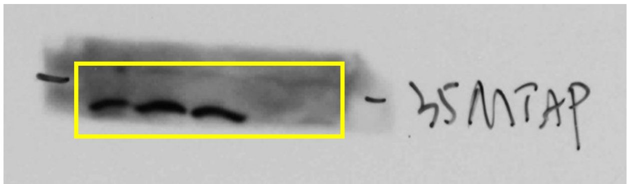

GAPDH

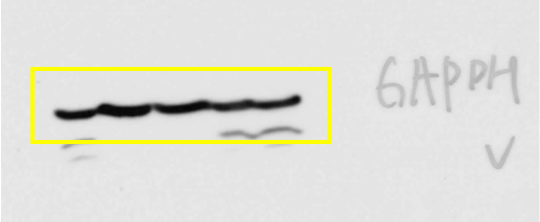

Full unedited gel for Supplemental Figure 4G

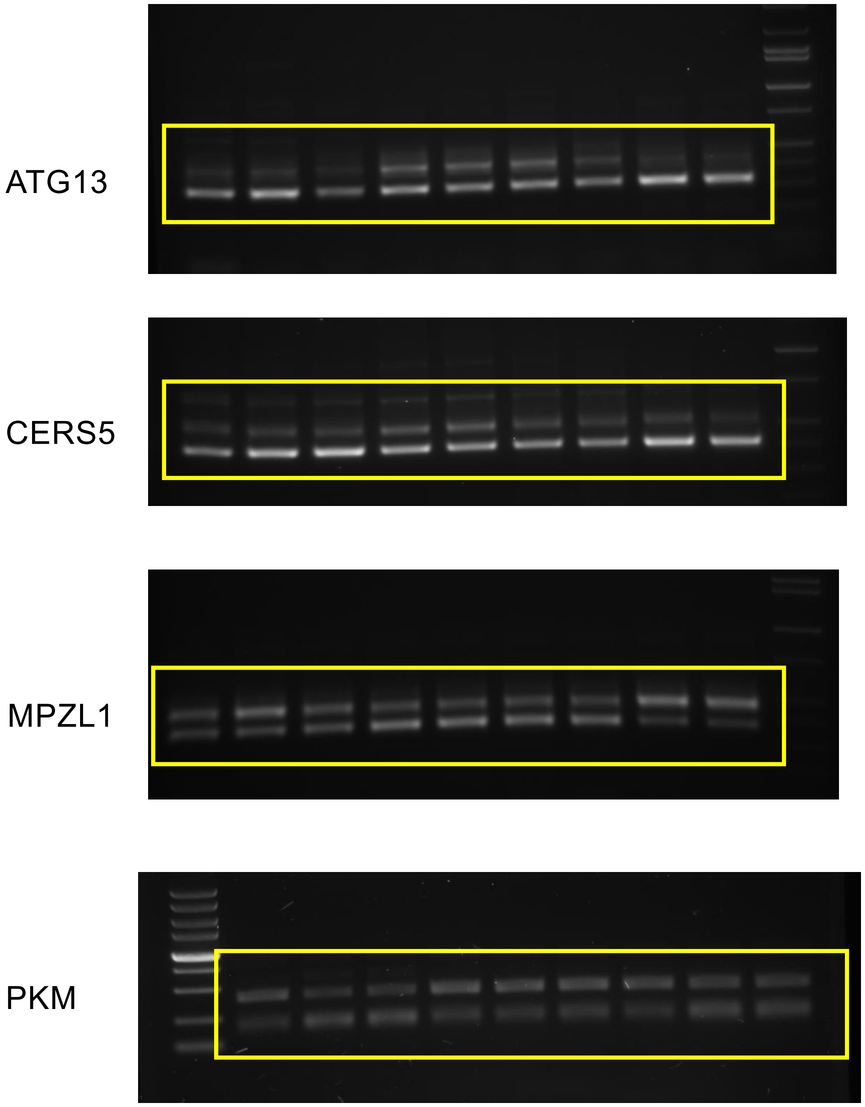

Full unedited gel for Supplemental Figure 4J

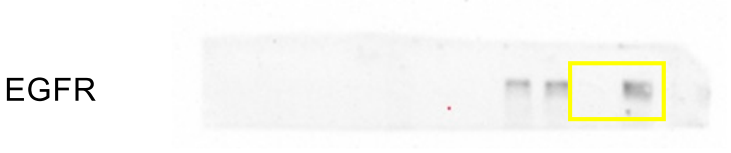

Full unedited gel for Supplemental Figure 4K

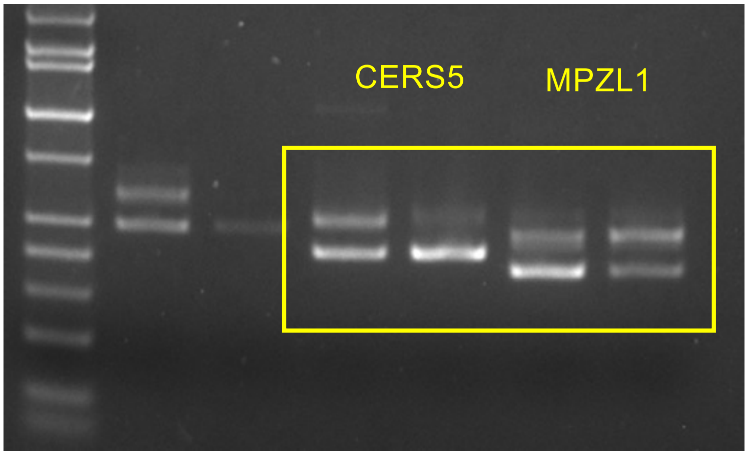

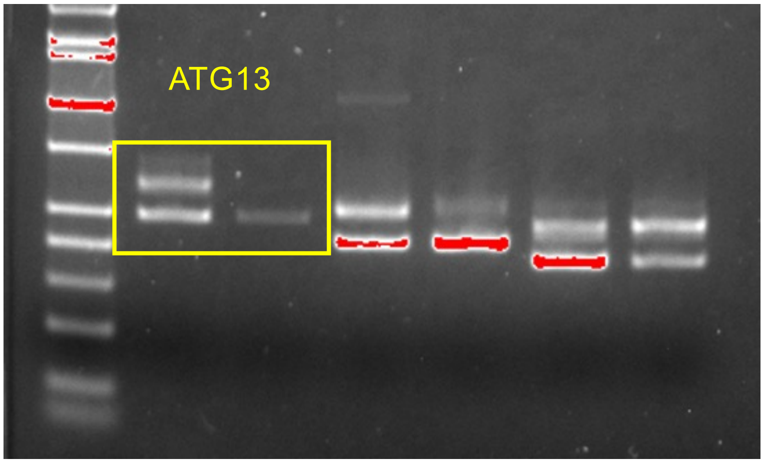

Full unedited gel for Supplemental Figure 4L

IDH1-R132H

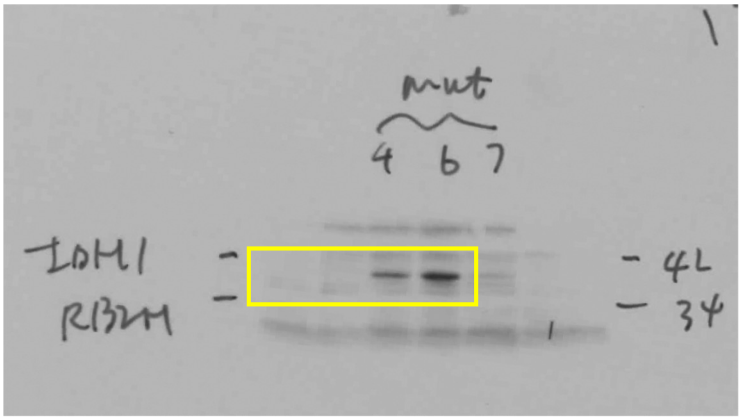

GAPDH

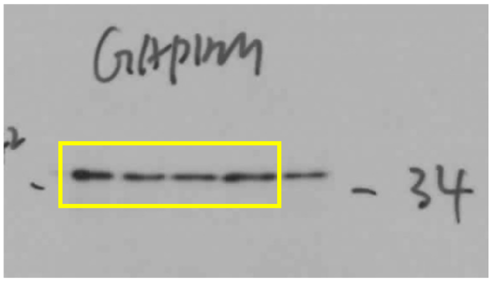

Full unedited gel for Supplemental Figure 4O

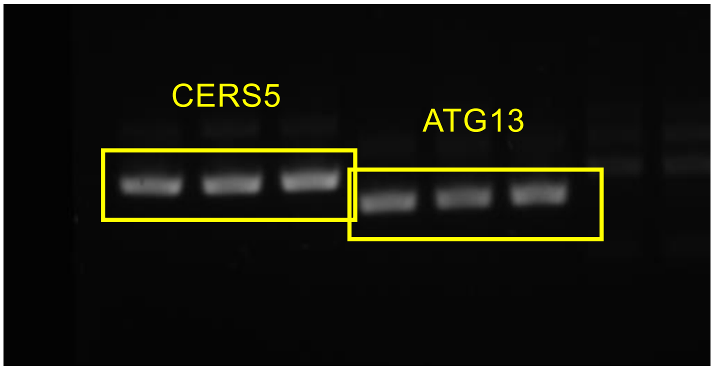

Full unedited gel for Supplemental Figure 5E

FLAG

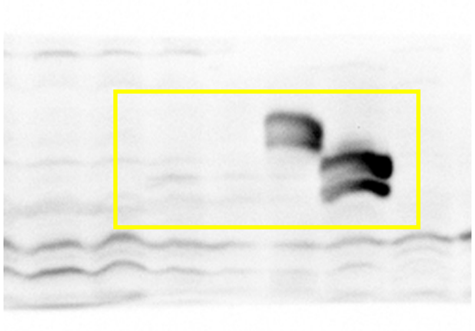

CERS5

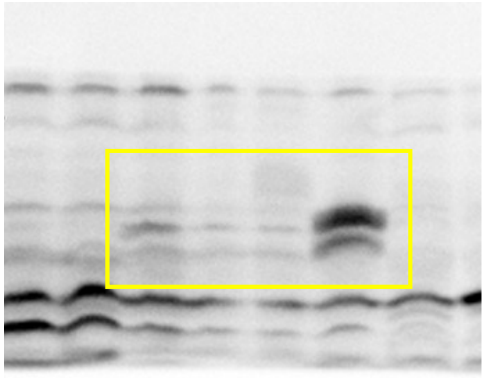

Full unedited gel for Supplemental Figure 5L

MPZL1

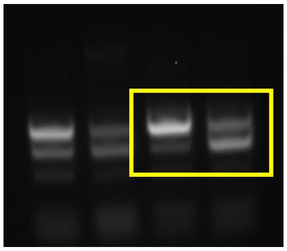

Full unedited gel for Supplemental Figure 5M

SHP2

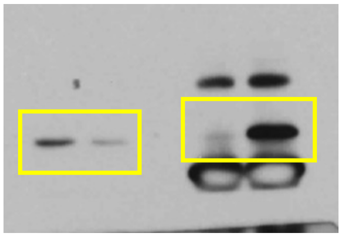

FLAG

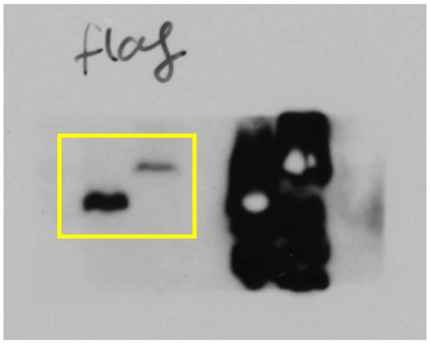

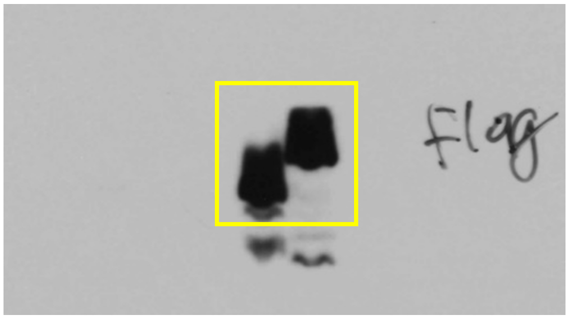

Full unedited gel for Supplemental Figure 7A

CELF4

GAPDH

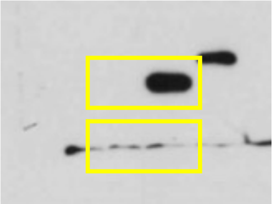

SRRM4

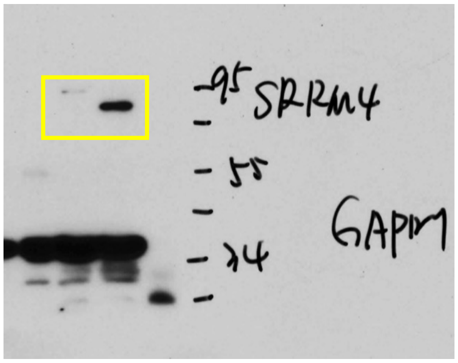

GAPDH

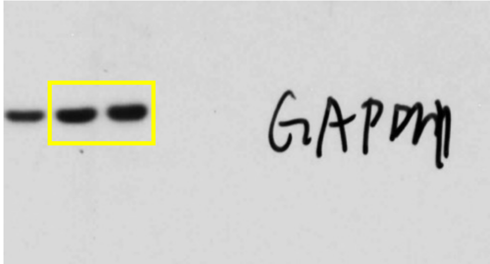

Full unedited gel for Supplemental Figure 7B, left panel

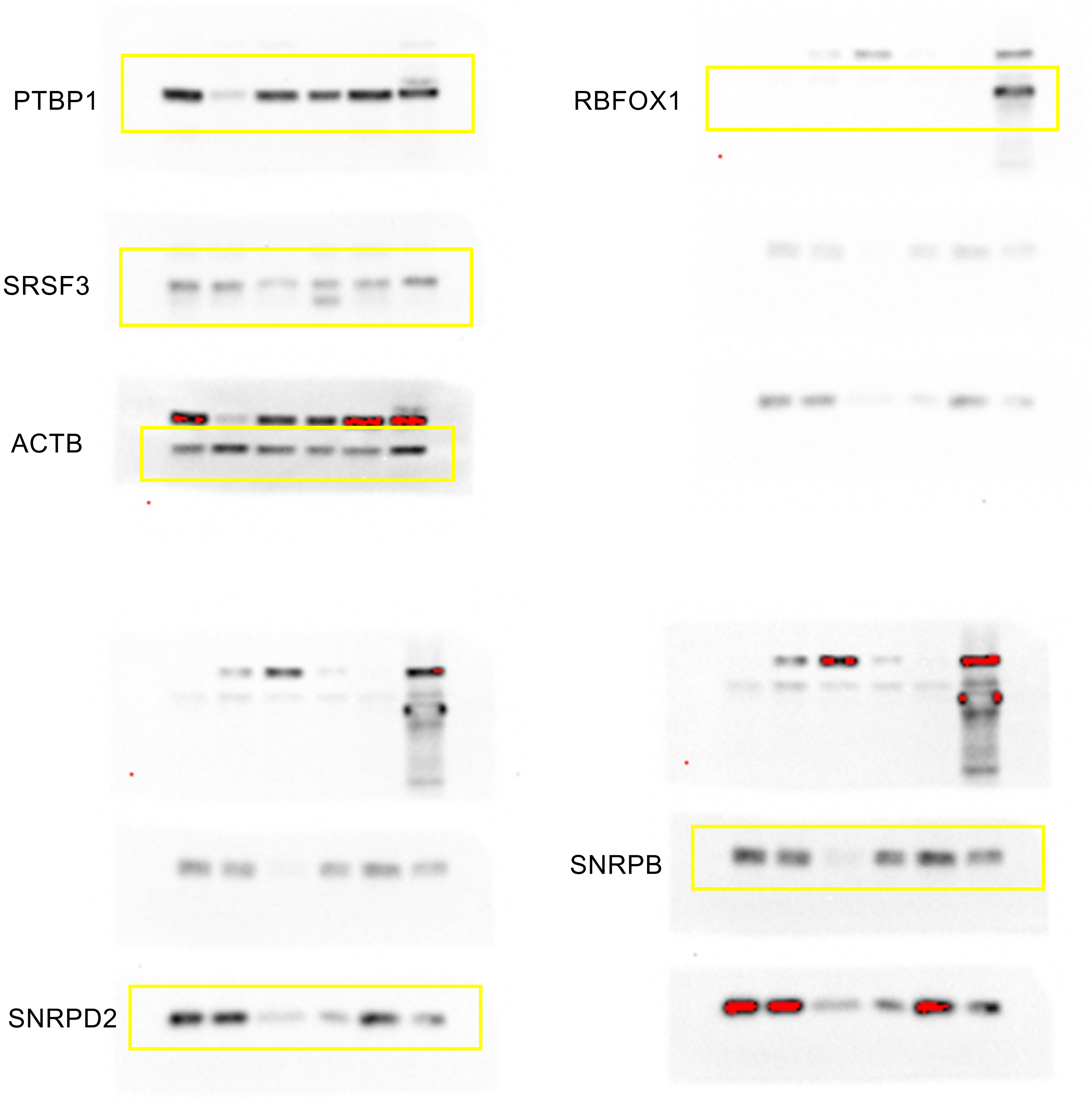

Full unedited gel for Supplemental Figure 7B, right panel

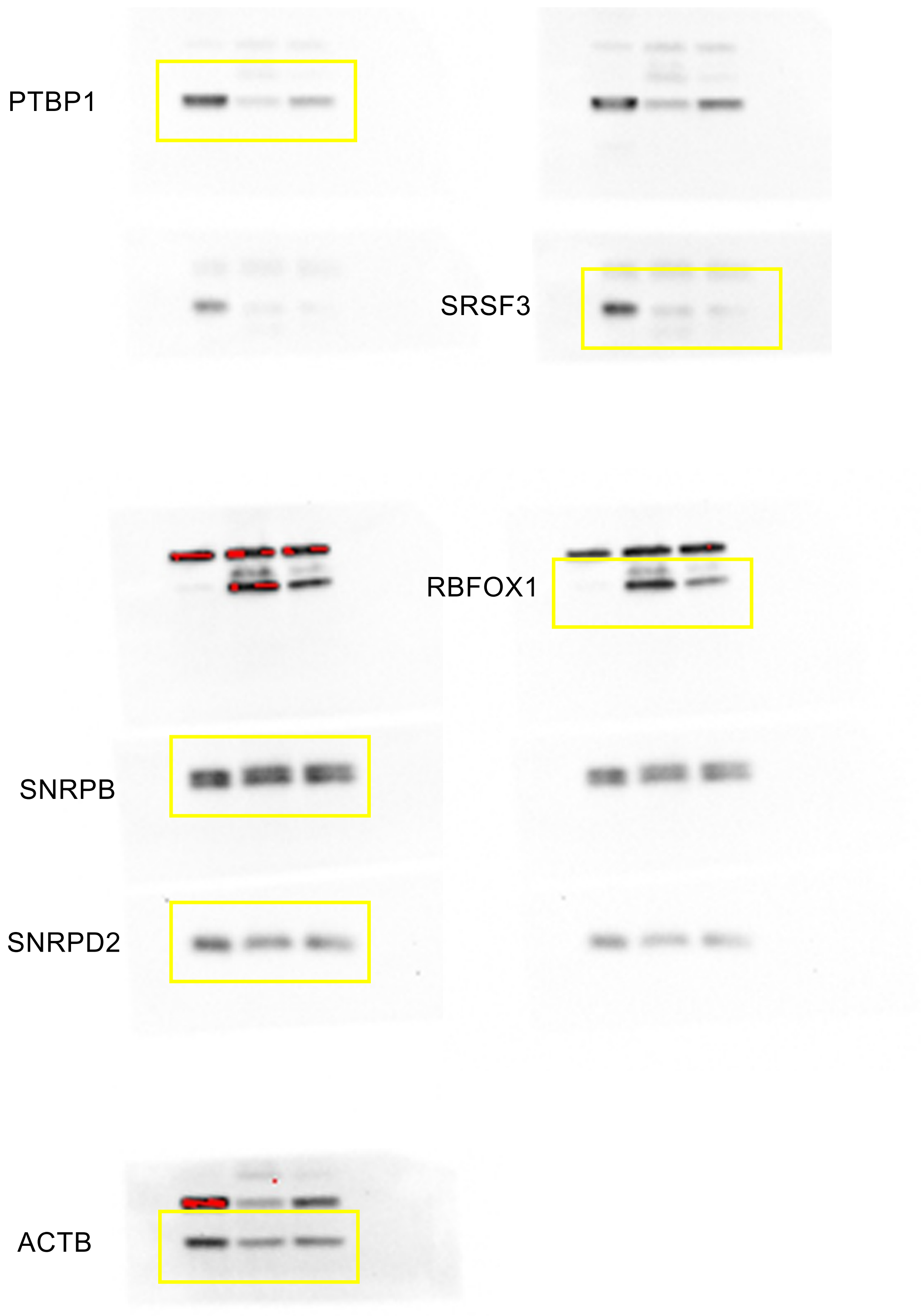

Full unedited gel for Supplemental Figure 7F

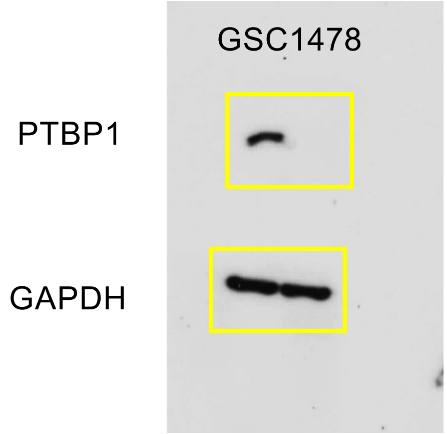

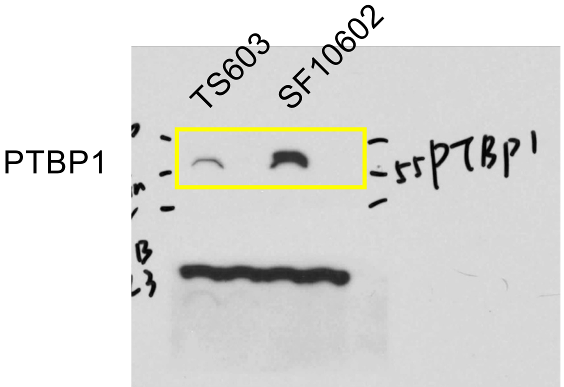

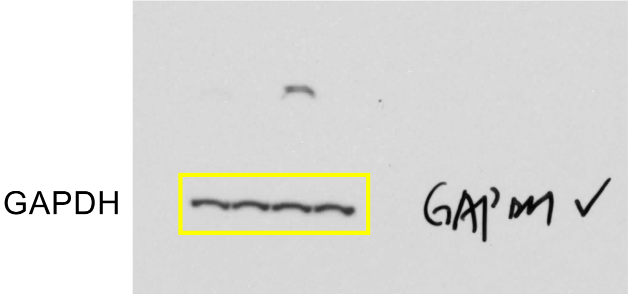

Supplement: Unedited blot and gel images [file jci-134-173789-s101.pdf]
